# Synthesis and Biological Evaluation of 4‑(4-Nitrophenyl)‑1*H*‑1,2,3-triazole Derivatives as Antitrypanosomal Agents

**DOI:** 10.1021/acsomega.4c11645

**Published:** 2025-05-13

**Authors:** Filipe Canto Oliveira, Luís Otávio Bunhotto Zamoner, Marcelo Dias Baruffi, Beatriz Silveira Augusto, Miguel de Menezes Vaidergorn, Maria Cristina Nonato, Thainá Silva Bologna, Marcos Vinicius da Silva, Ivone Carvalho

**Affiliations:** † Department of Pharmaceutical Sciences, School of Pharmaceutical Sciences of Ribeirão Preto, 28133 University of São Paulo, Av. Café s/n, Ribeirão Preto, SP 14040-903, Brazil; ‡ Department of Clinical, Toxicological and Bromatological Analysis, School of Pharmaceutical Sciences of Ribeirão Preto, University of São Paulo, Av. Café s/n, Ribeirão Preto, SP 14040-903, Brazil; § Center for the Research and Advancement in Fragments and molecular Targets (CRAFT), School of Pharmaceutical Sciences at Ribeirao Preto, University of São Paulo, Ribeirão Preto, SP 14040-903, Brazil; ∥ Protein Crystallography Laboratory, Department of Biomolecular Sciences, School of Pharmaceutical Sciences at Ribeirao Preto, University of São Paulo, Ribeirão Preto, SP 14040-903, Brazil; ⊥ Department of Microbiology, Immunology, and Parasitology, 74395 Federal University of Triangulo Mineiro, Av. Frei Paulino 30, Uberaba, MG 38025-180, Brazil

## Abstract

Chagas disease is endemic to 21 countries across the Americas, with an estimated 6–7 million people infected worldwide. Currently, only two drugs—benznidazole (BZN) and nifurtimox—are approved for treatment. While effective during the acute phase and in preventing mother-to-child transmission, these medications exhibit minimal to no efficacy in the chronic phase, require prolonged treatment courses, and are associated with significant side effects. Building on our previous work, we identified hit **1** (IC_50_ = 7 μM) as demonstrating superior in vitro activity against trypomastigotes forms of Trypanosoma cruzi Tulahuen strain compared to BZN (IC_50_ = 34 μM), along with a high selectivity index (SI = 114). To further investigate its potential, we synthesized 17 analogs, preserving the essential 4-(4-nitrophenyl)-1*H*-1,2,3-triazole scaffold crucial for antitrypanosomal activity, while modifying the *N*-benzylacetamide moiety. Preliminary screening for antitrypanosomatid activity and cytotoxicity against the T. cruzi Tulahuen strain identified compound **16** as the most promising candidate. This compound features a peracetylated galactopyranosyl unit in place of the *N*-benzylacetamide moiety, enhancing its potential for further development. It demonstrated potent activity against T. cruzi (IC_50_ 6 ± 1 μM, Tulahuen CL2 β-galactosidase strain) with no detectable cytotoxicity in mammalian cell lines. Compound **16** and its deprotected derivative, compound **19**, were further evaluated for their activity against T. cruzi intracellular amastigotes in infected LLC-MK2 (epithelial) and C2C12 (myoblast) cells. Noninfected control cells were exposed to the same treatment conditions to assess compound cytotoxicity (CC_50_). Notably, compound **16** (IC_50_: LLC-MK2, 0.16 ± 0.02 μM; C2C12, 0.13 ± 0.01 μM) and compound **19** (IC_50_: LLC-MK2, 0.10 ± 0.04 μM; C2C12, 0.11 ± 0.02 μM) exhibited higher antiamastigote activity with selectivity indices exceeding 400. In comparison, BZN, a first-line drug against T. cruzi, required significantly higher concentrations to achieve antiamastigote activity (IC_50_: LLC-MK2, 1.4 ± 0.1 μM; C2C12, 6 ± 1 μM) and exhibited lower selectivity indices, ranging from 14.4 to 32.8. The exceptional efficacy of compounds **16** and **19** was 33- to 36-fold greater than their activity against trypomastigotes, underscoring their strong therapeutic potential during the chronic phase. Additionally, pre-exposure of LLC-MK2 and C2C12 cell lines to these compounds significantly reduced infection rates in both models, demonstrating potent residual antiamastigote activity. The ability of compounds **16** and **19** to lower the infection index in LLC-MK2 and C2C12 cells was further assessed in combination with BZN. All combinations outperformed BZN alone, yielding significantly reduced post-treatment parasite burdens even at the lowest concentrations (0.06 μM) of compounds **16** and **19**. As derivatives of benznidazole, both compounds were evaluated as substrates for T. cruzi nitroreductase (TcNTR) and displayed only residual activity. These results suggest that compounds **16** and **19** operate through mechanisms distinct from BZN, with their combined inhibitory activity likely arising from synergic effects. Finally, both compounds were tested against *Recombinant trans-sialidase from* T. cruzi (TcTS), considering the role of galactose units in parasite mucins as acceptors of host sialic acid during the TcTS-catalyzed transfer reaction. The deprotected galactosyl derivative **19** was identified as a weak TcTS inhibitor, with an IC_50_ of 1.1 ± 0.1 mM. We presented novel galactosyl-4-(4-nitrophenyl)-1*H*-1,2,3-triazole derivatives that demonstrate high efficacy and selectivity, showing potential for treating both acute and chronic phases of Chagas disease. These compounds represent promising leads for the development of new drug candidates to address this neglected disease.

## Graphical abstract

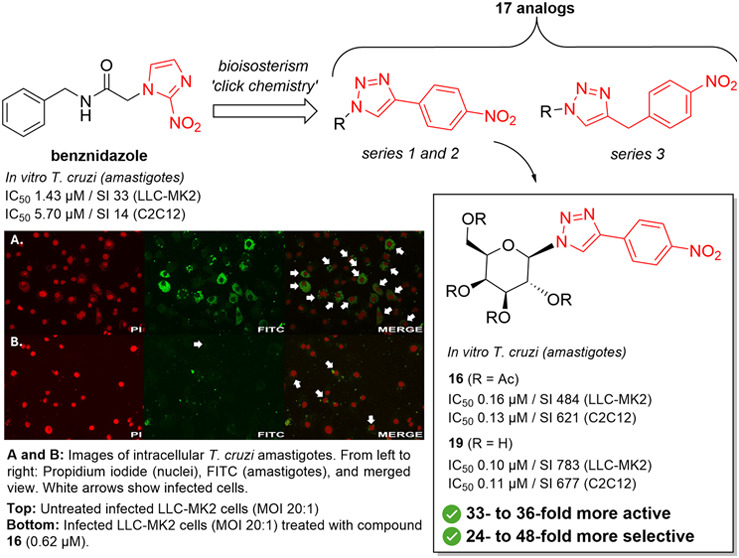

## 1 Introduction

Chagas disease (CD), also known as American trypanosomiasis, is caused by the parasitic protozoan Trypanosoma cruzi (T. cruzi) and is classified as a neglected tropical disease (NTD) by World Health Organization (WHO).^1^ The disease is endemic in 21 countries throughout the Americas; however, due to migration of infected individuals it has spread to several countries, resulting in an estimation of 6–7 million people infected worldwide.^2^ Despite its significant global burden, CD has historically attracted minimal commercial interest from the pharmaceutical industry for the development of new treatments,^3^ essential to eradicate the parasite in the acute phase and also reduce the rate of parasympathetic nervous system impairment, which may eventually result in megaesophagus, megacolon and accelerated dilated cardiomyopathy in the chronic phase.^4^

Currently, only two drugs—benznidazole (BZN) and nifurtimox—are approved for treating CD. Though effective in the acute phase and in preventing mother-to-child transmission, these medications show limited to no efficacy in the chronic phase, necessitate prolonged treatment durations, and are associated with significant side effects.^5^ Although, efforts led by organizations such as the Drugs for Neglected Diseases Initiative (DNDi) and WHO have resulted in some progress, such as the development of a safer pediatric formulation of BZN,^6^ clinical trials of potential new therapies have, to date, produced unsatisfactory outcomes. This includes drug repurposing attempts for antifungal azoles such as posaconazole, itraconazole, and ravuconazole; allopurinol, typically used to decrease high blood uric acid levels; and, most recently, the promising antimicrobial fexinidazole.^7^ Despite demonstrating an acceptable safety profile and a significant reduction in parasite load following treatment, most patients experienced relapse starting 10 weeks after treatment, leading to the discontinuation of fexinidazole’s development as a monotherapy for T. cruzi infection. Conversely, innovative oxaborole derivatives like AN15368, a more effective analog of the previously reported AN4169, have emerged as promising new candidates. AN15368 demonstrated efficacy both in vitro and in vivo against various T. cruzi lineages, achieving consistent curative results in nonhuman primates with long-term infections. Furthermore, the treatment caused no acute toxicity or long-term health or reproductive issues in these animals, positioning it as a validated candidate for future clinical trials.^8^ Additionally, GNF6702, a specific inhibitor of the kinetoplastid proteasome with an oxazole core, has shown effectiveness in treating rats in the chronic phase of Chagas disease and is now undergoing preclinical trials for toxicity evaluation.^9^ Therefore, there remains an urgent need for finding drugs with superior efficacy to BZN, safe (no genotoxicity, teratogenicity), with minimal drug interactions, and available in an oral formulation,^10^ that can address the diverse phases and manifestations of the disease.

The early events of parasite signaling, attachment, and entry into host cells involve a range of mechanisms, including membrane-mediated pathways and the formation of parasitophorous vacuoles. These processes are facilitated by various parasite cell surface components such as extracellular matrix elements, vesicles, surface mucins, oligopeptidase B, *trans*-sialidase, cruzipain, phospholipases, and Ca^2+^ release. Host cell surface receptors, including galectin-3 and Toll-like receptors, also play roles in these interactions. Notably, the GPI-anchored glycoprotein *trans*-sialidase of T. cruzi­(TcTS) is crucial for adhesion, invasion, and immune evasion, particularly during the infectious trypomastigote stage, by actively interacting with extracellular proteins such as laminin, fibronectin, collagen, vimentin, and cytokeratin.^11^ In addition to its primary role in catalyzing a unique transglycosylation reaction, where sialic acid is transferred from host glycoconjugates to T. cruzi galactosyl-glycoproteins through TcRab11, TcTS also plays crucial roles in escaping from vacuoles, evading both innate and acquired immunity, and influencing parasite tissue tropism.^12,13^ Therefore, identifying new inhibitors targeting T. cruzi cell surface components could be critical for developing selective therapies against the parasite.

Motivated by this lack of therapeutic alternatives, our research group published a previous work^14^ presenting the synthesis and testing of 27 analogs of *N*-benzyl-2-(2-nitro-1*H*-imidazol-1-yl)­acetamide (benznidazole or BZN) for their activity against T. cruzi. Remarkably, one of these analogs, hit **1**, exhibited superior in vitro activity (IC_50_ 7 ± 2 μM) compared to BZN against trypomastigotes forms of T. cruzi Tulahuen strain (*LacZ*) (IC_50_ 34 μM) and displayed a high selectivity index ( SI 114) (Figure 1). It differs from BNZ by the bioisosteric replacements of the 1*H*-imidazolyl moiety with a 1,4-disubstituted 1,2,3-triazolyl group and the nitro substituent of nitroimidazolyl with a nitrophenyl group accomplished via copper­(I)-catalyzed azide–alkyne cycloaddition (CuAAC). However, challenges related to the pharmacokinetic properties of hit **1**, particularly its low solubility in water, hindered the achievement of conclusive in vivo activity results. Nevertheless, in light of the promising results attained by hit **1** and its privileged chemical structure for combating T. cruzi, we decided to investigate alternatives to overcome these limitations and continue to study this class of compounds.

Therefore, in this study, we present the synthesis and biological evaluation of 17 analogs of hit **1**. The screening of the compounds against T. cruzi Tulahuen strain (trypomastigotes, photometric method) showed analog **16**, a galactopyranosyl 4-(4-nitrophenyl)-1*H*-1,2,3-triazole derivative, with exceptional anti-trypomastigote activity with an IC_50_ of 6 ± 1 μM against T. cruzi, while showing no cytotoxic effects in mammalian cells. This prompted further investigation into its *anti-* T. cruzi intracellular amastigotes activity using a more comprehensive assay, along with exploring its potential role as an inhibitor of T. cruzi’*s* cell surface *trans*-sialidase. The antiamastigote activities of analog **16 (**IC_50_: LLC-MK2, 0.16 ± 0.02 μM; C2C12, 0.13 ± 0.01 μM) and its deacetylated form, **19 (**IC_50_: LLC-MK2, 0.10 ± 0.04 μM; C2C12, 0.11 ± 0.02 μM) were confirmed, as well as high selectivity index (superior to 400) and the relative affinity of the analog **19** for *trans*-sialidase, suggesting a potential relationship with its mechanism of action. Furthermore, computational predictions indicate that analogs **16** and **19** exhibit improved pharmacokinetic parameters compared to hit **1**, potentially addressing the solubility issues previously encountered.

## 2 Results and Discussion

### 2.1 Molecular Design

Following a meticulous examination of the chemical structure of hit **1** and considering our group’s prior findings,^14^ it became evident that the 4-(4-nitrophenyl)-1*H*-1,2,3-triazole scaffold was essential for its antitrypanosomal activity (Figure 1). This conclusion arose from observations that modifications that were made involving the chemical reduction, removal, or replacement of the nitro group led to a significant decrease or complete loss of activity (Figure S1). Additionally, alterations in the position of the nitro group from *para-* to *ortho-* or *meta-* resulted in activity loss, as did modifications shifting the 1,2,3-triazole from 1,4 to 1,5-disubstitution. For this reason, the structural modifications in hit **1** were designed to keep the *para-* position of the NO_2_ aromatic group and the 1,4 disubstitution of the 1*H*-1,2,3-triazole.

Thus, three main series of analogs were designed (Scheme 1), ranging from conservative to more significant alterations in hit **1**. The first series intended to introduce new functional groups exclusively into the *N*-benzylacetamide portion of the molecule, while largely retaining its original structure. The second series also maintained intact the 4-(4-nitrophenyl)-1*H*-1,2,3-triazole scaffold, but underwent significant alterations in the *N*-benzylacetamide moiety. Finally, in the third series, a CH_2_ linker was introduced between the 1*H*-1,2,3-triazole and the *p*-nitrophenyl (while retaining the 1,4 disubstitution), accompanied by a complete modification to the *N*-benzylacetamide moiety.

The design of the analogs was also guided by *in silico* predictions,^15^ with the aim of identifying compounds possessing optimal pharmacokinetic properties and high leadlikeness (Table S1). The majority of the designed analogs adhered to Lipinski’s rule of five, exhibited high predicted gastrointestinal (GI) absorption, and none were predicted to be blood-brain barrier (BBB) permeant, key characteristics desired for an oral antitrypanosomal drug to treat Chagas disease.^10^ Moreover, most of the analogs had predicted Log *P* values lower than or comparable to Hit **1** and were classified as either moderately soluble or soluble in water. An important exception was analog **16**, which did not fully adhere to Lipinski’s rule of five and had low predicted GI absorption. However, it exhibited a desirable Log *P* value of 0.70, comparable to BZN’s 0.49, and was classified as water-soluble. Therefore, we also include it for synthesis as an analog of hit **1**.

Of the 17 analogs, the synthesis of 8 (analogs **15**, **16**, **17**, **19**, **21**, **23**, **24**, and **26**) has been previously described in the literature;^16–29^ however, to the best of our knowledge, none have been tested for or associated with antitrypanosomal activity. For instance, compound **16** appears in two studies^30,31^ focused on the development of new catalysts for triazole formation, with no mention of biological activity. Conversely, compound **19** has been tested as an inhibitor of human lysosomal α-glucosidase^32^ and as a glycogen phosphorylase inhibitor.^33^ Despite this, it was not among the most active compounds in either study, and no further investigation was pursued.

**Figure 1:**
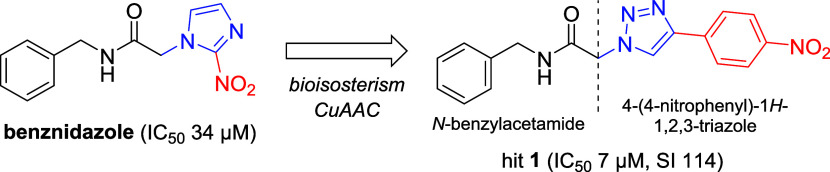
Modifications to BZN chemical structure that led to hit **1**, using bioisosterism concepts and CuAAC. IC_50_ and SI values against trypomastigotes forms of T. cruzi Tulahuen strain (LacZ).

### 2.2 Synthesis

In general, the synthesis followed a similar route to that for the preparation of hit **1** (Scheme 1a).^14^ For series 1, chloroacetamides **2–5** were prepared via condensation of their corresponding primary amine with 2-chloroacetyl chloride (32–95%), followed by treatment with sodium azide to give azidoacetamide intermediates (**6–9**) (49–95%). Finally, a CuAAC was conducted between azidoacetamide intermediates **6–9** and the commercially available terminal alkyne, 1-ethynyl-4-nitrobenzene, resulting in the target analogs **10**-**13** (13–97%).

The formation of the desired 1,4-disubstituted triazoles was confirmed by proton nuclear magnetic resonance spectroscopy (^1^H NMR) for compounds previously described in the literature, while newly synthesized compounds were characterized using ^1^H NMR, carbon-13 nuclear magnetic resonance spectroscopy (^13^C NMR), and high-resolution mass spectrometry (HRMS) (see Supporting Data). The use of a Cu­(I) catalyst exclusively yields the 1,4-disubstituted triazole, preventing the formation of 1,5-regioisomers.^34^ Its formation was confirmed by the chemical shift of the C–H triazole attached to the p-nitrobenzene ring, observed in the range of 8.94 to 8.62 ppm. An exception is compound **16**, which may experience an anisotropic effect due to the proximity of the carbonyl group from the O-acetyl protective group, resulting in a shift to 8.23 ppm. As previously reported,^14^ 1,5-disubstituted regioisomers with a p-nitrobenzene substituent exhibit the C–H triazole signal at a more shielded field, around 8.08 ppm.

As depicted in Scheme 1b, the condensation of methylene-azides comprising a methyl ester, benzonitrile or 2,3,4,6-tetra-*O*-acetyl-β-d-galactopyranosyl units with the 1-ethynyl-4-nitrobenzene, under CuAAC reaction, afforded series 2, compounds **14–16** (30–86%). Subsequently, hydrolysis of compounds **14**, **15** and **16** under distinct basic conditions led to the synthesis of the corresponding carboxylic acids **17** (75%), **18** (90%) and the deacetylated glycoside **19** (91%) through the well-known Zemplen procedure (sodium methoxide in methanol).^35^

Finally, series 3 was synthesized using the general procedure outlined in Scheme 1c. First, azide intermediate **20** was prepared by treatment of 1-(bromomethyl)-4-nitrobenzene with NaN_3_ (82%). Next, azide intermediate **20** underwent coupling with various commercially available terminal alkynes through a CuAAC reaction, resulting in analogs **21–28** (23–75%).

### 2.3 Screening for Antitrypomastigote Activity

A preliminary assay was conducted to evaluate the analogs’ in vitro cytotoxicity and antitrypanosomatid activity against T. cruzi Tulahuen strain (Table 1). Among all synthesized analogs, compound **16**, a peracetylated galactopyranosyl 4-(4-nitrophenyl)-1*H*-1,2,3-triazole derivative, emerged as the most promising, demonstrating consistent efficacy against T. cruzi (IC_50_ 6 ± 1 μM) with no observed cytotoxicity in mammalian cells. The second most active analog, compound **22**, showed significant antitrypanosomal activity, IC_50_ of 4.17 μM, but it was detrimental for MRC-5 cells owing to the comparable cytotoxic (CC_50_ 5.06 μM), leading to a lack of selectivity. However, the promising results of analog **16** encouraged us to conduct more in-depth studies on this compound.

**Scheme 1:**
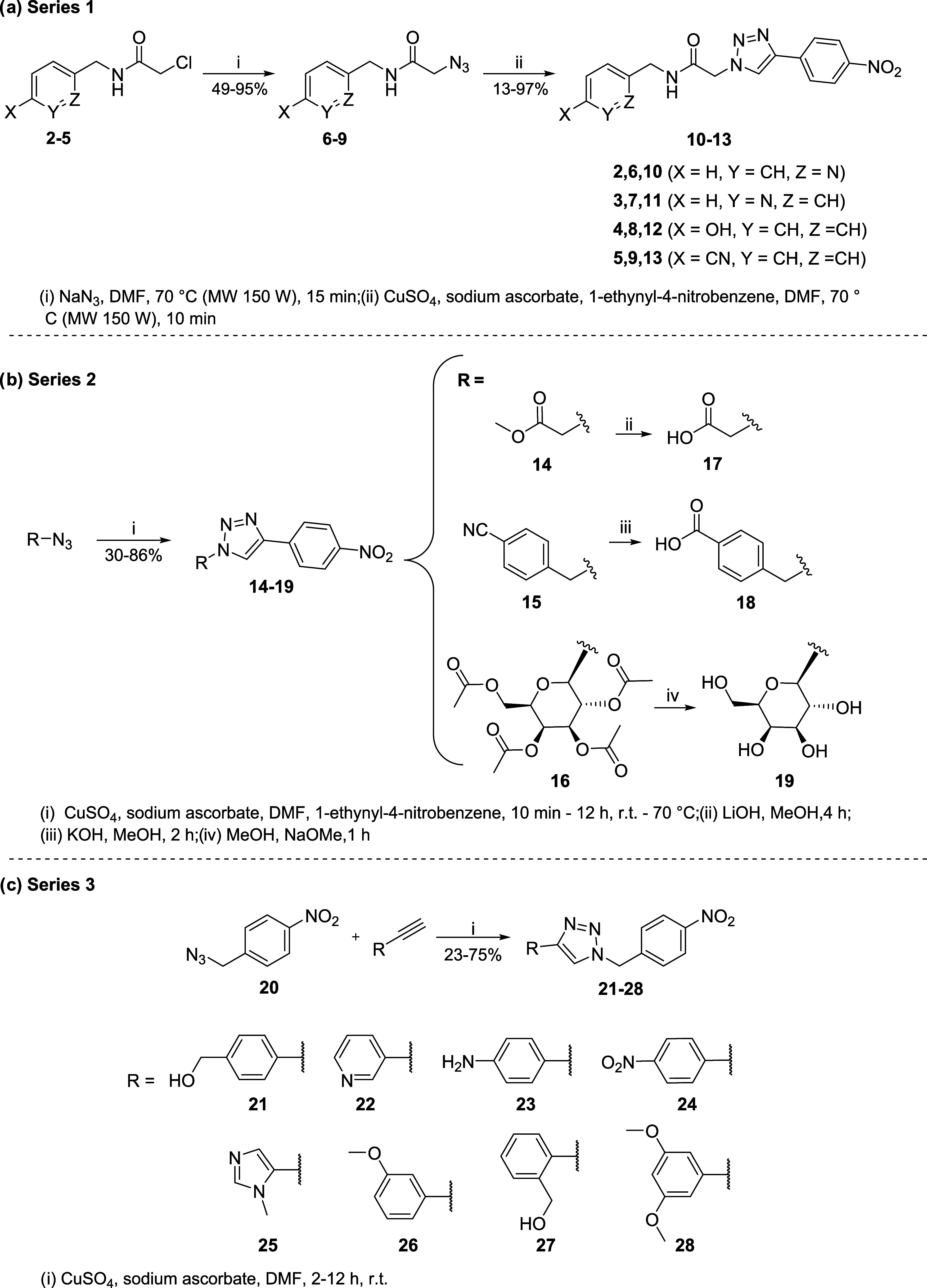
Synthesis of the Three Series of Analogs of Hit **1** ^a^

a Overall yield of each analog is provided in Table S2.

**Table 1:** In Vitro *Anti-* T. cruzi (Trypomastigote) Activity and Cytotoxicity Assessment of Hit **1** Analogs^a^

Cpd.	MRC-5 CC_50_ (μM)	PMM CC_50_ (μM)	T. cruzi IC_50_ (μM)
Series 1			
**10**	*>50.00*	*>50.00*	*>50.00*
**11**	*>50.00*	*>50.00*	*>50.00*
**12**	>50.00	>50.00	(31 ± 5)
**13**	*>50.00*	35.36	*>50.00*
Series 2			
**15**	*>50.00*	*>50.00*	*>50.00*
**16**	>50.00	>50.00	(6 ± 1)
**17**	*>50.00*	*>50.00*	*>50.00*
**18**	*>50.00*	*>50.00*	*>50.00*
Series 3			
**21**	*>50.00*	*>50.00*	*>50.00*
**22**	5.06	*>50.00*	4.17
**23**	*>50.00*	*>50.00*	*>50.00*
**24**	*>50.00*	*>50.00*	*>50.00*
**25**	*>50.00*	*>50.00*	*>50.00*
**26**	*>50.00*	*>50.00*	*>50.00*
**27**	*>50.00*	*>50.00*	*>50.00*
**28**	*>50.00*	*>50.00*	*>50.00*

a IC_50_: inhibitory concentration for 50% of parasites. CC_50_: cytotoxic concentration for 50% of mammalian cells. Cytotoxicity was measured against the human MRC-5 cell line and primary mouse macrophages (PMM). Values in italics represent the outcome of a single experiment. Values in parentheses represent the mean ± SEM from two independent experiments (original + follow-up experiment). Reference validation (IC_50_): tamoxifen 11.39 μM (MRC-5) and benznidazol 1.64 μM (T. cruzi).

### 2.4 Intracellular Amastigotes Activity

Considering the dynamics of T. cruzi infection in humans, characterized by an increase in parasitic load through intracellular replication of amastigote forms in various cell types, we evaluated the action of the compounds in LLC-MK2 (epithelial cells) and C2C12 (myoblasts) using an in vitro infection model. Both cell lines were plated at a concentration of 1 × 10^4^ cells/mL and infected with cell-derived trypomastigotes (MOI 20:1, Colombian strain) overnight. After this incubation, extracellular parasites were removed, and the cells were maintained for 48 h at 37 °C with 5% CO_2_ to allow differentiation and multiplication of amastigotes.

The cells were subsequently treated with serially decreasing concentrations of compounds **16** and **19** (ranging from 80 to 0.08 μM) or BZN (ranging from 66.2 to 0.12 μM). The antiamastigote activity was assessed by calculating the infection index, defined as the percentage of infected cells multiplied by the mean number of amastigotes per cell. Noninfected control cells were subjected to identical treatment conditions to evaluate the cytotoxicity of the compounds (CC_50_). This comprehensive approach allowed for the simultaneous assessment of antiparasitic efficacy and compound-induced cytotoxicity, offering valuable insights into their therapeutic potential.

Both tested cell lines maintained high viability even at the highest concentrations of compounds **16** and **19** (CC_50_ > 80 μM). Notably, **16** (IC_50_: LLC-MK2, 0.16 ± 0.02 μM; C2C12, 0.13 ± 0.01 μM) and **19** (IC_50_: LLC-MK2, 0.10 ± 0.04 μM; C2C12, 0.11 ± 0.02 μM) demonstrated potent antiamastigote activities with selectivity indices exceeding 400 (Figure 2 and Table 2). In contrast, treatment with BZN, a first-line *anti*-T. cruzi drug, exhibited higher toxicity for both cell lines (CC_50_: LLC-MK2, 46.86 μM; C2C12, 74.39 μM), required higher concentrations for antiamastigote activity (IC_50_: LLC-MK2, 1.43 ± 0.1 μM; C2C12, 6 ± 1 μM), and showed lower selectivity indices (ranging from 14.4 to 32.8). This remarkable efficacy of compounds **16** and **19** was significantly greater—ranging from 33- to 36-fold—compared to their activity against trypomastigotes, underscoring their strong therapeutic potential at chronic phase by their high specificity for intracellular amastigotes.

**Figure 2:**
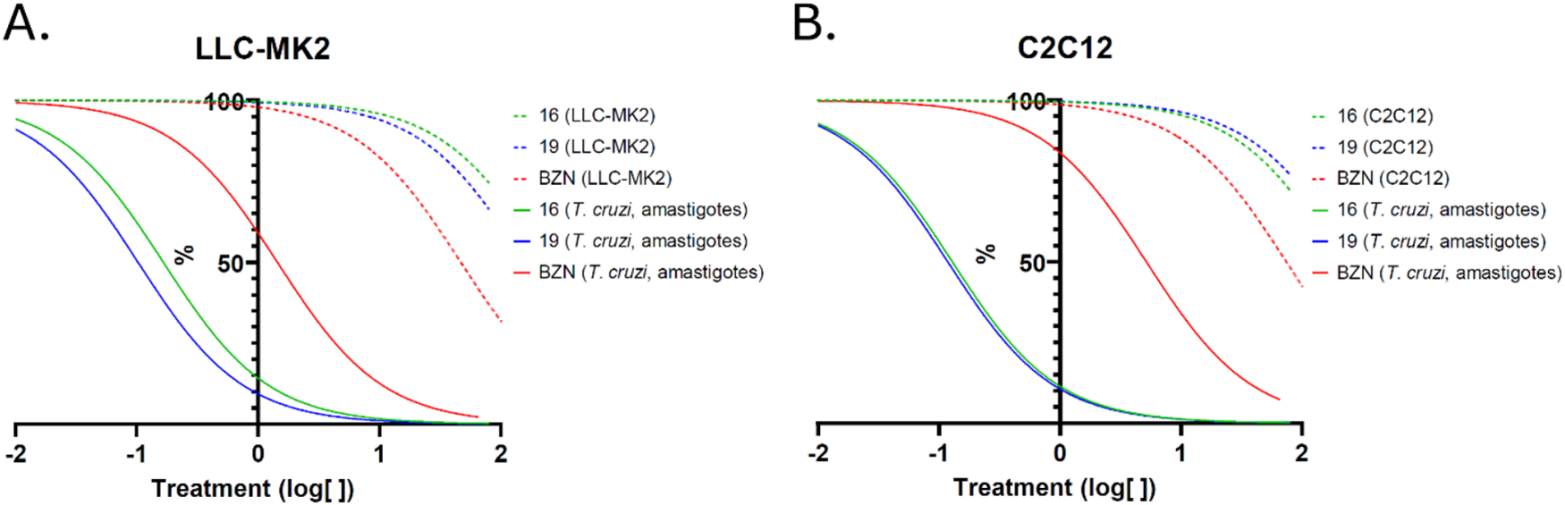
Evaluation of the antiamastigote activities of compounds **16** and **19**. (A, B): Nonlinear regression curves for CC_50_ and IC_50_ determination (log­[inhibitor] vs normalized response) in LLC-MK2 and C2C12 cell lines, respectively.

**Table 2:** In Vitro *Anti*-T. cruzi Amastigote Activity and Cytotoxicity Assessment in LLC-MK2 and C2C12 Cell Lines

	non infected (μM)		+T.cruzi		selective index	
cell treatment after infection	MK2	C2C12	MK2	C2C12	MK2	C2C12
Compound **16** (CC_50_/IC_50_ ^a^)	>80	>80	0.16 ± 0.02	0.13 ± 0.01	484.3	621.1
Compound **19** (CC_50_/IC_50_ ^a^)	>80	>80	0.10 ± 0.04	0.11 ± 0.02	782.8	677.4
BZN (CC_50_/IC_50_ ^a^)	46.86	74.39	1.4 ± 0.1	6 ± 1	32.8	14.4

a log­(inhibitor) vs normalized response.

The enhanced efficacy of the compounds against T. cruzi amastigote forms is particularly advantageous, as trypomastigotes dominate only during the acute phase of infection. In contrast, chronic infection is driven by the persistence of tissue-resident amastigotes, which play a critical role in sustaining the disease over time.^36,37^ This targeted activity aligns with therapeutic goals focused on mitigating the chronic phase and controlling parasite reservoirs.^37,38^ The superior efficacy of *anti*-T. cruzi compounds against amastigote forms may be attributed to the extended exposure duration (48 h), which likely facilitates intravacuolar accumulation of the compounds and amplifies their effects over time.^39,40^ This prolonged interaction, coupled with the high in vitro tolerability of the evaluated cell lines, likely enhances the compounds’ cumulative impact, optimizing their antiparasitic activity.

To assess whether pre-exposure of cells to the compounds could result in a reduced parasitic load postinfection, the LLC-MK2 and C2C12 cell lines were plated at a concentration of 1 × 10^4^ cells/mL and treated with serially decreasing concentrations of compounds **16** and **19** (ranging from 80 to 0.62 μM). After 24 h, the culture medium was removed and replaced with fresh medium containing cell-derived trypomastigotes (MOI 20:1, Colombian strain) overnight. Following this incubation, extracellular parasites were removed, and the cells were maintained for 48 h at 37 °C in a 5% CO_2_ atmosphere to allow differentiation and proliferation of amastigotes.

Pre-exposure of LLC-MK2 and C2C12 cell lines resulted in reduced infection indices in both models, demonstrating potent residual antiamastigote activity with selectivity indices ranging from 39.4 to 141.5 (Figure 3 and Table 3). The observed reduction in parasitic load, even when infection occurred post-treatment, might result from a combination of mechanisms previously related to triazole derivatives, including intracellular accumulation of the compounds, direct action on newly internalized trypomastigotes and subsequently on amastigotes,^41,42^ TcTS inhibition, phagosome formation, and cytosolic escape.^43,44^ Additionally, these effects could involve the induction of autophagic pathways and oxidative stress responses.^45^

**Figure 3:**
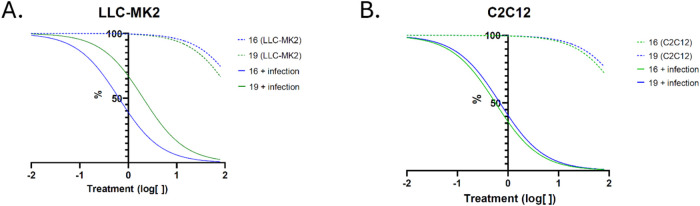
Evaluation of antiamastigote activity in pretreated LLC-MK2 and C2C12 cell lines. (A, B): Nonlinear regression curves for CC_50_ and IC_50_ determination (log­[inhibitor] vs normalized response).

**Table 3:** In Vitro *Anti*-T. cruzi Amastigote Activity of Compounds **16** and **19** in Preexposed LLC-MK2 and C2C12 Cell Lines

	non infected (μM)		+T. cruzi (μM)		selective index	
cell treatment previously to infection	MK2	C2C12	MK2	C2C12	MK2	C2C12
Compound **16** (CC_50_/IC_50_ ^a^)	>80	>80	0.6 ± 0.2	0.56 ± 0.09	124.9	141.5
Compound **19** (CC_50_/IC_50_ ^a^)	>80	>80	2.0 ± 0.2	0.7 ± 0.3	39.4	115.6

a log­(inhibitor) vs normalized response.

To delve deeper into these significant findings, we recognize that a crucial aspect of drug discovery for infectious diseases is identifying compounds that demonstrate effective action at minimal concentrations. This approach offers clear economic advantages and reduces the risks of drug resistance development and cumulative toxicity.^46,47^ Beyond establishing the effective doses of individual compounds, combining them with drugs already in clinical use can produce synergistic or additive effects, enhancing the desired therapeutic activity even at suboptimal doses of the individual agents.

In this context, we evaluated the efficacy of combining compounds **16** and **19** (at concentrations ranging from 0.25 to 0.06 μM) with BZN (3.8 μM) in reducing the infection index in LLC-MK2 and C2C12 cells. Our results showed that all combinations were more effective than BZN alone, significantly lowering the post-treatment parasite burden. Notably, even the lowest concentration (0.06 μM) of compounds **16** and **19** was effective, with LLC-MK2 cells demonstrating greater sensitivity to the combined treatment (Figure 4). This highlights the potential of combinatorial approaches to maximize therapeutic outcomes while minimizing dosage requirements. Isobolographic analysis of the combinations of BZN with compounds **16** or **19** in the MK2 and C2C12 cell lines indicates a significant synergistic interaction (Table 4). This synergy is evidenced by the effective doses (ED50) of the combinations, which show a reduction in the required doses to achieve the desired effect compared to individual IC_50_. These results suggest that the coadministration of BZN with compounds **16** and **19** can potentially enhance therapeutic efficacy due to the observed synergy and result in lower required doses for treatment, thereby minimizing potential adverse effects.

**Figure 4:**
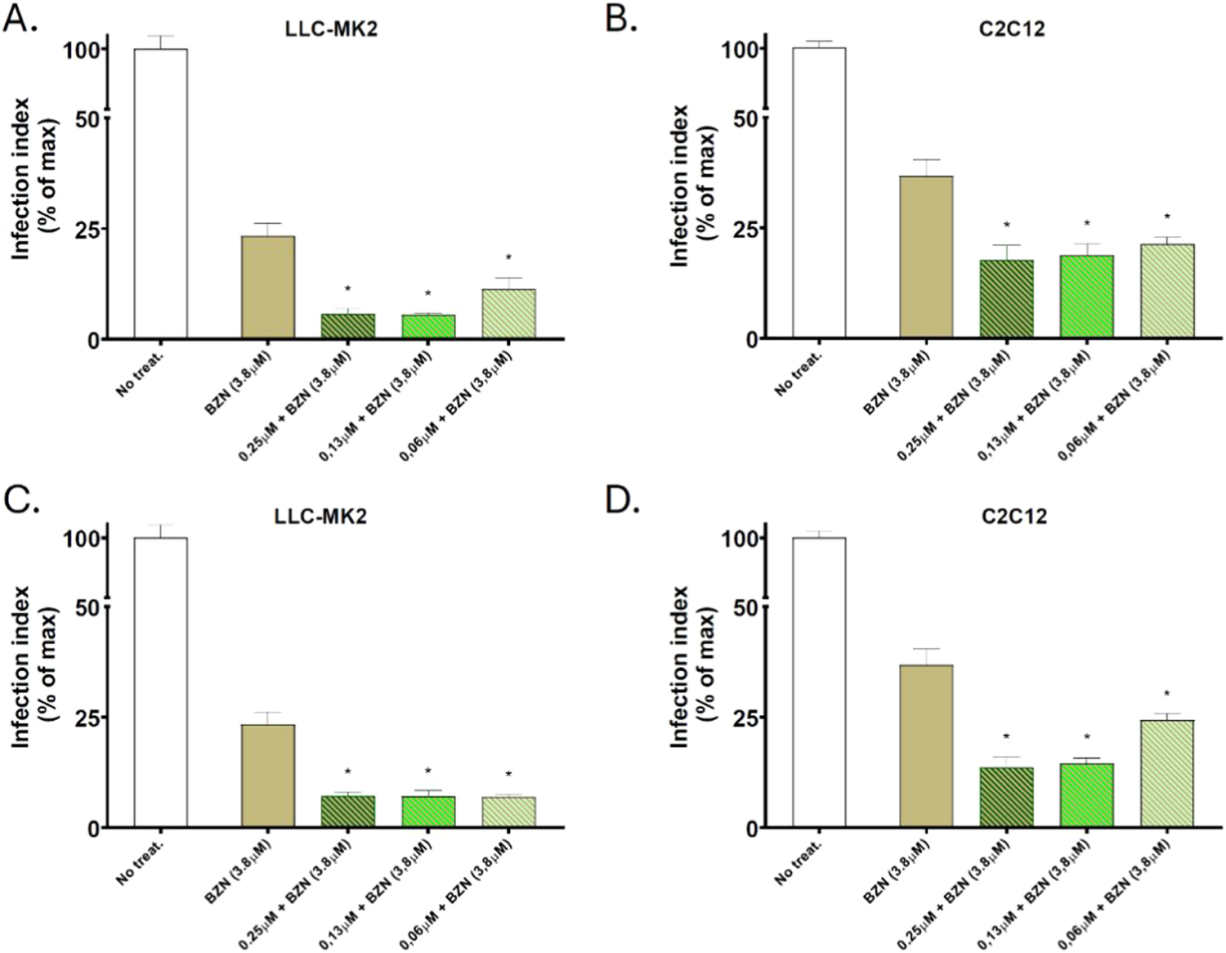
Evaluation of antiamastigote activity of compounds **16** and **19** combined with BZN. (A, B): Antiamastigote activity of compound **16** combined with BZN in LLC-MK2 and C2C12 cell lines. (C, D): Antiamastigote of compound **19** combined with BZN in LLC-MK2 and C2C12 cell lines. *p < 0.05; statistical analysis performed using one-way ANOVA with Dunnett’s post-test.

**Table 4:** Isobolographic Analysis of the Combination of Benznidazole (BZN) with Compounds **16** and **19** in MK2 and C2C12 Cell Lines^a^

cell line	drug/combination	ED_50_ (CI)	required BZN dose (μM)	required compound **16** dose (μM)	required compound **19** dose (μM)	interaction
MK2	BZN + compound **16**	0.23	0.12	0.013		synergy
MK2	BZN + compound **19**	0.28	0.09		0.006	synergy
C2C12	BZN + compound **16**	0.10	0.13	0.002		synergy
C2C12	BZN + compound **19**	0.08	0.11		0.002	synergy

a CI values indicate the type of interaction: synergy (CI 1). ED values represent the effective doses required to achieve 50% inhibition.

### 2.5 T. cruzi Nitroreductase Activity (TcNTR)

Benznidazole (BZN), a nitro-containing heterocyclic compound used for the treatment of Chagas disease, is metabolized by Type I nitroreductases, leading to the formation of highly reactive metabolites.^48^ Compounds **16** and **19**, which are nitro derivatives of BZN, were investigated for their potential activity in the cellular models studied here, exploring a mechanism similar to that observed for the prodrugs that inspired the synthesis of the tested molecules.

The NTR activity, measured in terms of *K*_obs_, was assessed for compounds **16** and **19**, using BZN as a control (Table 5).^49^ The results suggested residual activity for these compounds. Considering the cellular results, namely, the increased activity of benznidazole in the presence of compounds **16** and **19**, and the enzymatic assays showing low activity of compounds **16** and **19** as substrates, it is possible that the observed inhibitory activities arise from distinct mechanisms, with the results reflecting the synergic effects of two independent processes.

**Table 5:** TcNTR Activity of Compounds **16** and **19**, Using BZN as a Control^a^

compound	*K*_obs_ (s^–1^) (100 μM)	*K*_obs_ (s^–1^) (50 μM)	*K*_obs_ (s^–1^) (25 μM)	*K*_obs_ (s^–1^) (12.5 μM)
**16**	0.07 ± 0.01	0.07 ± 0.01	0.06 ± 0.01	0.02 ± 0.02
**19**	0.04 ± 0.01	0.04 ± 0.02	0.03 ± 0.01	0.03 ± 0.01
**BZN**	0.23 ± 0.07	0.32 ± 0.04	0.17 ± 0.05	0.13 ± 0.01

a Data represent an average of three replicates for each measurement of TcNTR activity for the tested compounds. The apparent Kobs (rate constant) is expressed as value in s^–1^, according to the equation *K*_obs_ = *V*_0_/[enzyme]. *V*_0_ (initial velocity) = [NADH oxidized]/time. BZN, benznidazole.

### 2.6 T. cruzi *trans*-Sialidase Inhibition (TcTS)

Regarding the chemical structure, compounds **16** and **19** belong to the second series of analogs, where the 4-(4-nitrophenyl)-1*H*-1,2,3-triazole scaffold was retained, but a galactopyranosyl unit replaced the *N*-benzylacetamide moiety. Considering the role played by galactose units on parasite mucins as acceptors of host sialic acid during the TcTS-transfer catalyzed reaction, we were intrigued to verify whether compounds **16** and **19** showed potential TcTS inhibition.^11–13^

Current TcTS inhibitors can interact with the acceptor site, the donor site, or both enzyme subsites.^50^ Some inhibitors, derived from the sialic acid substrate or the transition state analogue DANA, have shown limited potency.^51^ For example, Zanamivir and Oseltamivir, which are effective against viral sialidase neuraminidase, exhibit weak inhibition of *trans-*sialidase in the millimolar range.^52^ Modifications of the hydroxyl group of sialic acid at C-2 have led to irreversible inhibitors with weak activity, as demonstrated by temporary enzyme inhibition with compounds like 2,3-difluorosialic acid (*K*_i_ = 20 mM) and Neu5NAcFNP (*K*_i_ = 10 mM). Similarly, sialic acid analogs with a fluoro group at C-3 and modifications to the glycerol chain, such as umbelliferyl, 4-(phenyl carbamide)-butyramide, and benzamide, have also shown inhibitory activity in the millimolar range.^53^ On the other hand, inhibitors that interact with the acceptor site or both enzyme subsites tend to be more potent. For instance, lactitol, an acceptor analog molecule, has demonstrated IC_50_ values of 0.57 mM.^54^ However, it only weakly inhibits cell infection in in vitro assays, with 20–27% inhibition. Additionally, novel aryl α-aminophosphonates have been identified as potent inhibitors of TcTS, with an IC_50_ of 0.21 μM.^55^ These compounds were designed to target all three key binding motifs of *trans*-sialidase (sialic acid, carboxylate, and lactose) and have been shown to act in a noncompetitive manner according to kinetic analysis. However, their in vitro efficacy against T. cruzi remains to be investigated.

Although the main reaction catalyzed by TcTS involves the transfer of host sialic acids to β-galactose acceptors found in parasite surface mucins, a residual hydrolase activity is also present and was utilized to screen a small library of TcTS inhibitors. To this end, a classical continuous fluorimetric method was employed to measure the ability of TcTS to hydrolyze the 2-(4-methylumbelliferyl)-α-d-n-acetylneuraminic acid (MuNANA) substrate, releasing the fluorescent methylumbelliferone (Mu) in the presence of the most active antitrypanosomal compounds, galactosyl-4-(4-nitrophenyl)-1*H*-1,2,3-triazoles **16** and **19**.^56^

Preliminary screening of compound **16**, assayed in the 2.000 to 0.062 mM concentration range, yielded inconsistent results due to its low solubility in the medium, even in the presence of 0.5% DMSO. The noticeable precipitation of the peracetylated galactosyl derivative **16** in the wells, combined with the need to maintain enzyme activity, sensitive to higher DMSO concentrations, led us to focus the experiments on the unprotected product **19**, with comparable T. cruzi antiamastigote inhibition. The evaluation of **19** was performed in the presence of 0.5% DMSO and provided a reliable estimation of inhibition when compared to pyridoxal phosphate, a classical TcTS inhibitor. The rate of enzymatic activity was calculated by linear regression, and the slope values for each curve were standardized to inhibition percentage (Figure 5A). Further experiments, using eight different concentrations (ranging from 4.0 to 0.015625 mM) under the previously established conditions, revealed the deprotected galactosyl derivative **19** as a weak TcTS inhibitor with an IC_50_ of 1.1 ± 0.1 mM, based on three independent experiments performed on two different days (Figure 5B).

**Figure 5:**
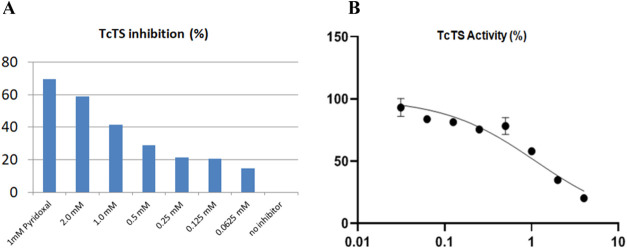
In vitro TcTS enzymatic assays in the presence of compound **19**. (A) TcTS inhibition percentage, using pyridoxal phosphate as the standard reference to compare inhibitory effects. (B) Activity percentage of TcTS. The data represent an average of three replicates for each measurement of TcTS activity for the tested compound **19**, repeated on two different days.

Although TcTS inhibition was not pronounced, the dose–response curve revealed weak to moderate activity compared to reported inhibitors. This suggests that compound **19** may influence parasite invasion and exhibit some antitrypanosomal activity, potentially acting as a sialic acid acceptor substrate in place of parasite mucins. However, the precise mechanism of action of these compounds is still under investigation, and it is possible that multiple mechanisms are at play simultaneously. For instance, the 1,2,3-triazole ring has also been associated with antiparasitic activity in various studies involving triazole derivatives.^57–60^

## 3 Conclusions

We successfully designed and synthesized 17 derivatives of hit **1**, with improved pharmacokinetics parameters and high leadlikeness. The compounds were divided into three main groups of modifications, aiming to preserve the pharmacophore features of hit **1**, as the critical 4-(4-nitrophenyl)-1*H*-1,2,3-triazole scaffold for antitrypanosomal activity while varying the *N*-benzylacetamide moiety.

The compounds were evaluated for their in vitro cytotoxicity and antiparasitic activity against T. cruzi. Notably, compound **16**, related to the galactopyranosyl derivative 4-(4-nitrophenyl)-1*H*-1,2,3-triazole, demonstrated consistent antitrypanosomatid activity against T. cruzi without exhibiting cytotoxicity in mammalian cells. To further investigate the efficacy of compound **16** and its corresponding unprotected derivative, compound **19**, intracellular antiamastigote activities were evaluated using two cell lines associated with Chagas disease progression: LLC-MK2 and C2C12. Both compounds showed remarkable inhibition, exceeding that of BZN—a first-line drug for the treatment of T. cruzi—by one to 2 orders of magnitude. Furthermore, the selectivity indices of the target compounds were significantly high, exceeding 400, compared with the selectivity indices of BZN, which ranged from 14.4 to 32.8. Comparable results were obtained when LLC-MK2 and C2C12 cell lines were pretreated with compounds **16** and **19**, showing reduced infection indices in both models. This underscores their potential to protect cells from infection while preserving antiamastigote activity. Additionally, to enhance therapeutic outcomes, combining BZN with compounds **16** or **19** demonstrated synergic effects, evidenced by reduced post-treatment parasite burdens and indicating the potential for lower drug dosages and fewer side effects. The lack of NTR activity in the presence of both compounds indicates distinct mechanisms compared to BNZ metabolism.

Considering the presence of a galactopyranosyl unit in compounds **16** and **19**, which may act as a TcTS inhibitor and interfere with the sialylation of T. cruzi mucins essential for parasite invasion, we evaluated the enzyme’s activity using a classical continuous fluorimetric method. Although the inhibition was modest (IC_50_ 1.1 ± 0.1 mM), it was significant when compared to reported data on other compounds, suggesting some influence on the mechanism of action. However, further studies are needed to fully understand their mechanisms and biological activity.

## 4 Experimental Section

### 4.1 General Methods

All acquired chemicals were used without additional purification. Thin-layer chromatography (TLC) was performed using silica gel coated aluminum plates (60 F254, Merck) with visualization in ultraviolet (UV) (λ = 254 nm) on a Spectroline CM-10 instrument. Manual column chromatography was performed using silica gel (40–63 μm, MERCK). A Biotage Isolera MPLC system with normal phase prepacked silica gel cartridges was used for flash chromatography. The microwave-assisted reactions were performed in sealed tubes on a CEM Discover Microwave System. IR spectra (KBr disks) were recorded on a Thermo FT-IR Nicolet Protege 460 spectroscope instrument. Only relevant absorption maxima, *v*_max_ (cm^–1^), are listed throughout as “s” (strong), “m” (medium) or “w” (weak). Ultraviolet–visible (UV–vis) and fluorescence spectra were recorded on a Molecular Devices SpectraMax-M2 instrument. Final products were purified via high-performance liquid chromatography (HPLC) employing a semipreparative reverse phase C18 column (Torrance CA, Luna C18, 5 μ, 250 × 10 mm^2^), in a Shimadzu instrument (Prominence, model LC-20A), equipped with detector DAD SPDM20A and controlled by LabSolutions 5.93 software. A uniform HPLC was applied for all target compounds. The mobile phase consisted of acetonitrile and water, initiating with 10% acetonitrile, and ramping up to 100% acetonitrile over a 30 min runtime. The chromatography was carried out at room temperature (25 °C) with a constant flow rate of 3 mL/min. High resolution mass spectra (HRMS) were obtained in a Bruker Daltonics micrOTOF-QII ESI-TOF instrument. NMR spectra were recorded in CDCl_3_ or DMSO-*d*_6_ at 25–30 °C on Bruker Avance DPX-300 MHz, Bruker Ultra Shield 300FT-NMR, DRX-400 MHz and DRX-500 MHz spectroscopes instruments operating at 300, 400, or 500 MHz and 75, 100, or 125 MHz for ^1^H and ^13^C nuclei respectively. All chemical shifts (δ values) are given in parts per million (ppm); all homocoupling patterns (*^n^J*_H_,_H_ values) are given in Hertz (Hz). No TMS was added: chemical shifts were referred against the solvent peak: CDCl_3_, δ_H_ (7.26 ppm) and δ_C_ (39.52 ppm); DMSO-*d*_6_, δ_H_ (2.54 ppm) and δ_C_ (77.36 ppm). ^1^H-multiplicities were abbreviated as singlet (s), broad singlet (bs), doublet (d), triplet (t), quartet (q), multiplet (m), doublet of doublets (dd), doublet of doublet of doublets (ddd), and doublet of triplets (dt).

### 4.2 Synthesis of the First Group of Analogs

#### 4.2.1 General Procedure for Preparation of Chloroacetamide Intermediates (**2–5**)

To a round-bottom flask, placed in an ice–water bath, were added chloroacetyl chloride (1.5 equiv), an appropriate organic solvent (DCM, DMF or acetonitrile), the corresponding primary amine, (1.0 equiv), a base (Et_3_N or K_2_CO_3_) (1.2 equiv) and a magnetic stir bar. The bottle was capped, and the reaction mixture was stirred for 24 h. Over the course of the reaction, the ice bath was allowed to melt and warm to rt. The solvent was then evaporated to dryness. The resulting mixture was diluted with water and extracted with EtOAc. The combined organic layers were washed with a saturated aqueous NaCl solution, dried over MgSO_4_, filtered, and evaporated to dryness.

##### 4.2.1.1 2-Chloro-*N*-(pyridin-2-ylmethyl)­acetamide (**2**)^61,62^

Solvent: DCM. Primary amine: 2-picolylamine. Base: K_2_CO_3_. Product: brown oil (355 mg, 95% yield). ^1^H NMR (300 MHz, CDCl_3_) δ 8.58 (d, *J* = 4.9 Hz, 1H), 7.88 (s, 1H), 7.69 (td, *J* = 7.7, 1.8 Hz, 1H), 7.31–7.18 (m, 2H), 4.61 (d, *J* = 5.0 Hz, 2H), 4.13 (s, 2H).

##### 4.2.1.2 2-Chloro-*N*-((pyridin-3-yl)­methyl)­acetamide (**3**)^61,62^

Solvent: acetonitrile. Primary amine: 3-picolylamine. Base: K_2_CO_3_. Product: brown oil (CAS 401640–80–0) (125.7 mg, 74% yield). ^1^H NMR (300 MHz, DMSO) δ 9.87 (s, 1H), 9.57 (s, 1H), 9.19 (s, 1H), 8.66 (d, *J* = 8.1 Hz, 1H), 8.14 (d, *J* = 7.4 Hz, 1H), 6.03 (s, 2H), 4.62 (s, 2H).

##### 4.2.1.3 *N*-(4-Hydroxybenzyl)-2-chloroacetamide (**4**)^14^

Solvent: DMF. Primary amine: 4-hydroxybenzylamine. Base: triethylamine. Product: red oil (CAS 52447–43–5). ^1^H NMR (CDCl_3_, 300 MHz) δ 7.26 (s, 1H), 7.17 (d, *J* = 8.5 Hz, 2H), 6.82 (d, *J* = 8.5 Hz, 2H), 4.42 (d, *J* = 5.8 Hz, 2H), 4.10 (s, 2H).

##### 4.2.1.4 2-Chloro-*N*-(4-cyanobenzyl)­acetamide (**5**)^63^

Solvent: DCM. Primary amine: 4-cyanobenzylamine. Base: triethylamine. Product: oil (CAS 340732–46–9) (50 mg, 32% yield). ^1^H NMR (300 MHz, DMSO) δ 8.87 (s, 1H), 7.80 (d, *J* = 8.1 Hz, 2H), 7.44 (d, *J* = 7.9 Hz, 2H), 4.38 (d, *J* = 6.0 Hz, 2H), 4.15 (s, 2H).

#### 4.2.2 General Procedure for Synthesis of Azidoacetamide Intermediates (**6–9**)^14^

To a round-bottom flask were added the corresponding chloroacetamide (**2–4**) (1 equiv), sodium azide (2.0 equiv), DMF and a magnetic stir bar. The reaction mixture was stirred for 15 min at 70 °C (open vessel system) under microwave irradiation (150 W). DMF was then coevaporation with toluene. The resulting solid was diluted with water and extracted with EtOAc. The combined organic layers were washed with a saturated aqueous NaCl solution, dried over MgSO_4_, filtered, and evaporated to dryness.

##### 4.2.2.1 2-Azido-*N*-(pyridin-2-ylmethyl)­acetamide (**6**)

Chloroacetamide: **2**. Product: dark orange oil (CAS 1367355–24–5) (228 mg, 62% yield). IR ν_max_/cm^–1^ 3350–3310 (NH), 2160–2120 (N3), 1680 (CO).

##### 4.2.2.2 2-Azido-*N*-((pyridin-3-yl)­methyl)­acetamide (**7**)

Chloroacetamide: **3**. Product: a brown oil (CAS 1544881–50–6) (51 mg, 49% yield). ^1^H NMR (300 MHz, CDCl_3_) δ 8.51 (s, 2H), 7.64 (d, *J* = 7.9 Hz, 1H), 7.33–7.23 (m, 1H), 7.13 (s, 1H), 4.48 (d, *J* = 6.0 Hz, 2H), 4.03 (s, 2H). ^13^C NMR (75 MHz, CDCl_3_) δ 166.92, 149.14, 149.02, 135.77, 133.41, 123.72, 52.56, 40.87.

##### 4.2.2.3 *N*-(4-Hydroxybenzyl)-2-azidoacetamide (**8**)

Chloroacetamide: **4**. Product: a brown oil (1.7148 g, 80% yield). ^1^H NMR (CDCl_3_, 300 MHz) δ 7.11 (d, *J* = 8.5 Hz, 2H), 6.80 (d, *J* = 8.5 Hz, 2H), 6.75 (s, 1H), 4.37 (d, *J* = 5.8 Hz, 2H), 4.02 (s, 2H).

#### 4.2.3 Synthesis of 2-Azido-*N*-(4-cyanobenzyl)­acetamide (**9**)^64^

A solution of 2-chloro-*N*-(4-cyanobenzyl)­acetamide (**5**) (10 mg, 0.043 mmol, 1.0 equiv) in 0.5 mL of DMF was cooled with an ice bath and then sodium azide (5.66 mg, 0.086 mmol, 2.0 equiv) was added. The bottle was capped, and the reaction mixture was stirred for 24 h. Over the course of the reaction, the ice bath was allowed to melt and warm to rt. The solvent was then evaporated to dryness. The resulting mixture was diluted with water and extracted three times with DCM. The combined organic layers were washed with a saturated aqueous NaCl solution, dried over MgSO_4_, filtered, and evaporated to dryness; resulting in compound **9** (inedited) (14.8 mg, 95% yield). ^1^H NMR (300 MHz, DMSO) δ 8.72 (s, 1H), 7.80 (d, *J* = 8.3 Hz, 2H), 7.46 (d, *J* = 8.3 Hz, 2H), 4.39 (d, *J* = 6.0 Hz, 2H), 3.93 (s, 2H).

#### 4.2.4 General Procedure for Microwave-Assisted CuAAC “Click Reaction”^14^

To a solution of the corresponding azide (1.0 equiv), 1-ethynyl-4-nitrobenzene (1.1 equiv) in DMF were added sodium ascorbate (0.1 equiv) and CuSO_4_ (1 mol L^–1^ aqueous solution) (0.03 equiv). The reaction mixture was stirred for 10 min at 70 °C (open vessel system) under microwave irradiation (150 W). DMF was then coevaporated with toluene. The resulting solid was diluted with water and extracted with EtOAc. The combined organic layers were dried over MgSO_4_, filtered, and evaporated to dryness.

##### 4.2.4.1 2-(4-(4-Nitrophenyl)-1*H*-1,2,3-triazol-1-yl)-*N*-(yridine-2-ylmethyl)­acetamide (**10**)

Azide: **6**. Product: solid (Inedited) (46.7 mg, 13% yield) ^1^H NMR (300 MHz, DMSO) δ 9.03 (s, 1H), 8.80 (s, 1H), 8.31 (d, *J* = 8.8 Hz, 2H), 8.13 (d, *J* = 8.8 Hz, 2H), 7.77 (q, *J* = 8.4 Hz, 1H), 7.32 (d, *J* = 14.3 Hz, 2H), 5.32 (s, 2H), 4.44 (d, *J* = 5.8 Hz, 2H). 13C NMR (151 MHz, DMSO) δ 166.1, 157.9, 149.3, 147.1, 144.8, 137.5, 137.3, 133.5, 128.8, 126.4, 125.5, 124.9, 52.3, 44.8. HRMS (ESI) *m*/*z* calculated for C_16_H_14_N_6_O_3_ + H^+^: 339.12001, found: 339.1206.

##### 4.2.4.2 2-(4-(4-Nitrophenyl)-1*H*-1,2,3-triazol-1-yl)-*N*-((pyridin-3-yl)­methyl)­acetamide (**11**)

Azide: **7**. Product: a brown solid (Inedited) (70 mg, 97% yield) ^1^H NMR (300 MHz, DMSO) δ 9.15 (t, *J* = 5.9 Hz, 1H), 8.86 (s, 1H), 8.50 (d, *J* = 16.0 Hz, 2H), 8.32 (d, *J* = 8.9 Hz, 2H), 8.15 (d, *J* = 8.9 Hz, 2H), 7.71 (d, *J* = 7.9 Hz, 1H), 7.38 (dd, *J* = 7.8, 4.7 Hz, 1H), 5.31 (s, 2H), 4.37 (d, *J* = 5.8 Hz, 2H). 13C NMR (75 MHz, DMSO) δ 166.0, 149.3, 148.7, 147.0, 144.7, 137.6, 135.7, 126.4, 125.6, 124.9, 124.0, 52.3, 40.8, 40.7, 40.5, 40.2, 39.9, 39.7, 39.4, 39.1. HRMS (ESI) *m*/*z* calculated for C_16_H_14_N_6_O_3_ + H^+^: 339.12001, found: 339.1200.

##### 4.2.4.3 *N*-(4-Hydroxybenzyl)-2-(4-(4-nitrophenyl)-1*H*-1,2,3-triazol-1-yl)­acetamide (**12**)

Azide: **8**. Product: an orange solid (inedited) (205 mg, 96% yield). ^1^H NMR (DMSO-*d*_6_, 300 MHz) δ 9.41 (s, 1H), 8.84 (s, 1H), 8.33 (d, *J* = 8.7 Hz, 2H), 8.16 (d, *J* = 8.8 Hz, 2H), 7.11 (d, *J* = 8.4 Hz, 2H), 6.74 (d, *J* = 8.4 Hz, 2H), 5.25 (s, 2H), 4.23 (d, *J* = 5.5 Hz, 2H). 13C NMR (75 MHz, DMSO) δ 165.0, 156.5, 146.6, 144.3, 137.2, 128.9, 128.8, 126.0, 125.2, 124.5, 115.1, 51.9, 42.1. HRMS (ESI) *m*/*z* calculated for C_17_H_15_N_5_O_4_ + H^+^: 354.11968, found: 354.1196.

##### 4.2.4.4 *N*-(4-Cyanobenzyl)-2-(4-(4-nitrophenyl)-1*H*-1,2,3-triazol-1-yl)­acetamide (**13**)

Azide: **9**. Product: a white solid (Inedited) (4.5 mg, 23% yield) ^1^H NMR (300 MHz, DMSO) δ 9.00 (s, 1H), 8.83 (s, 1H), 8.32 (d, *J* = 8.9 Hz, 2H), 8.15 (d, *J* = 9.0 Hz, 2H), 7.82 (d, *J* = 8.2 Hz, 2H), 7.50 (d, *J* = 8.1 Hz, 2H), 5.31 (s, 2H), 4.44 (d, *J* = 5.6 Hz, 2H). 13C NMR (151 MHz, DMSO) δ 165.9, 146.9, 144.8, 144.6, 137.2, 132.6, 128.3, 126.2, 125.3, 124.7, 119.1, 110.0, 52.1, 42.4. HRMS (ESI) *m*/*z* calculated for C_18_H_14_N_6_O_3_ + H^+^: 363.12001, found: 363.1205.

### 4.3 Synthesis of the Second Group of Analogs

#### 4.3.1 Synthesis of Precursor Methyl 2-Azidoacetate^65^

To a solution of methyl 2-chloroacetate (mg) in DMSO was added sodium azide (mg) and stirred for 20h in rt. Next, water was added to the reaction mixture and it was extracted 3× with DCM. The combined organic layers were washed with a saturated aqueous NaHCO_3_ solution followed by a saturated aqueous NaCl solution, dried over Na_2_SO_4_, filtered, and carefully evaporated to dryness to give the product methyl 2-azidoacetate as light yellow oil. The product was used in the next step without further purification due to its high volatility.

#### 4.3.2 Methyl 2-(4-(4-Nitrophenyl)-1*H*-1,2,3-triazol-1-yl)­acetate (**14**)

General procedure for CuAAC (4.2.4.). Azide: methyl 2-azidoacetate. Product: **14** (30% yield in 2 steps). ^1^H NMR (300 MHz, DMSO-*d*_6_) δ 8.83 (s, 1H), 8.33 (d, *J* = 9.0 Hz, 2H), 8.14 (d, *J* = 9.0 Hz, 2H), 5.53 (s, 2H), 3.74 (s, 3H).

#### 4.3.3 4-((4-(4-Nitrophenyl)-1*H*-1,2,3-triazol-1-yl)­methyl)­benzonitrile (**15**)

General procedure for CuAAC (4.2.4.). Azide: 4-(azidomethyl)-benzonitrile (100 mg, 0.6322 mmol). Product: solid (117 mg, 61% yield) ^1^H NMR (300 MHz, DMSO) δ 8.94 (s, 1H), 8.32 (d, *J* = 9.0 Hz, 2H), 8.12 (d, *J* = 9.0 Hz, 2H), 7.88 (d, *J* = 8.6 Hz, 2H), 7.52 (d, *J* = 8.6 Hz, 2H), 5.83 (s, 2H). IR ν_max_/cm^–1^ 2260–2222 (CN), 1550–1475 and 1360–1290 (NO_2_).

#### 4.3.4 (2*R*,3*S*,4*S*,5*R*,6*R*)-2-(Acetoxymethyl)-6-(4-(4-nitrophenyl)-1*H*-1,2,3-triazol-1-yl)­tetrahydro-2*H*-pyran-3,4,5-triyl Triacetate (**16**)^30^

General procedure for CuAAC (4.2.4.). Azide: 2,3,4,6-Tetra-*O*-acetyl-b-d-galactopyranosyl azide (50 mg, 0.134 mmol). Product: solid (60.1 mg, 86% yield) ^1^H NMR (300 MHz, CDCl3) δ 8.29 (d, *J* = 8.9 Hz, 2H), 8.23 (s, 1H), 8.04 (d, *J* = 8.9 Hz, 2H), 5.94 (d, *J* = 9.3 Hz, 1H), 5.60 (m, 2H), 5.31 (dd, *J* = 10.3, 3.21 Hz, 1H), 4.33–4.19 (m, 3H), 2.25 (s, 3H), 2.05 (s, 3H), 2.03 (s, 3H), δ1.92 (s, 3H). ^13^C RMN (151 MHz, DMSO) δ 20.31, 20.52, 20.67, 20.69, 61.21, 66.86, 67.92, 70.63, 74.26, 86.44, 119.58, 124.31 (2C), 126.45 (2C), 136.20, 146.24, 147.57, 169.32, 169.80, 169.93, 170.37. HRMS (ESI) *m*/*z* calculated for C_22_H_24_N_4_O_11_ + H^+^: 521,15143, found: 521,1516.

#### 4.3.5 2-(4-(4-Nitrophenyl)-1*H*-1,2,3-triazol-1-yl)­acetic Acid (**17**)

A solution of compound **14** (735 mg, 2.80 mmol) in 2 M lithium hydroxide (21 μL, 101 mg, 4,20 mmol, 1.5 equiv) and methanol (169 mL) was stirred at room temperature for 4h. The organic solvent was removed by rotary evaporation. After a dilute HCl solution (0.25 M, 150 mL) was added to the mixture, the precipitate formed was collected by suction filtration, washed with cold water, dried in vacuo, and then purified via Biotage system eluting with Hexane/EtOAc (gradient) to afford compound **17** (0.613 g, 75% yield) (CAS 95603–19–3). ^1^H NMR (300 MHz, DMSO) δ 8.63 (s, 1H), 8.24 (d, *J* = 9.1 Hz, 2H), 8.06 (d, *J* = 9.0 Hz, 2H), 5.24 (s, 2H). ^13^C NMR (75 MHz, DMSO) δ 168.9, 147.1, 144.9, 137.5, 126.4, 125.3, 124.9, 51.4.

#### 4.3.6 4-((4-(4-Nitrophenyl)-1*H*-1,2,3-triazol-1-yl)­methyl)­benzoic Acid (**18**)^66^

A solution of 4-((4-(4-nitrophenyl)-1*H*-1,2,3-triazol-1-yl)­methyl)­benzonitrile (**15**) (20 mg, 0.0655 mmol) in EtOH (1 mL) and KOH (7.35 mg, 1.131 mmol) was refluxed for 3h. The solvent was evaporated and the resulting solid was diluted with water and extracted 3x with EtOAc. The combined organic layers were dried over MgSO_4_, filtered, and evaporated to dryness, resulting in compound **18** (inedited) (19 mg, 90% yield) ^1^H NMR (300 MHz, DMSO) δ 8.92 (s, 1H), 8.31 (d, *J* = 9.0 Hz, 2H), 8.13 (d, *J* = 9.0 Hz, 2H), 7.88 (d, *J* = 8.4 Hz, 2H), 7.42 (d, *J* = 8.4 Hz, 3H), 5.75 (s, 2H). ^13^C NMR (151 MHz, DMSO) δ 168.1, 147.1, 145.3, 139.2, 137.4, 134.5, 128.5, 128.3, 126.5, 124.8, 124.2, 53.3. HRMS (ESI) *m*/*z* calculated for C_16_H_12_N_4_O_4_ – H^+^: 323.07858, found: 323.0781 IR ν_max_/cm^–1^ 3300–2500 (OH), 1720–1706 (CO), 1550–1475 and 1360–1290 (NO_2_).

#### 4.3.7 (2*R*,3*R*,4*S*,5*R*,6*R*)-2-(Hydroxymethyl)-6-(4-(4-nitrophenyl)-1*H*-1,2,3-triazol-1-yl)­tetrahydro-2*H*-pyran-3,4,5-triol (**19**)

The removal of the acetyl groups from compound **16** (31 mg, 0,06 mmol) was performed using sodium methoxide in methanol and addition of (1 mol/L^–1^) until pH 9.0 was reached. The reaction was monitored by TLC to track the complete removal of the acetyl groups from compound **16**. The mixture was neutralized with ion exchange DOWEX 50WX4–50 resin, filtered through a Celite pad, and concentrated in vacuo to obtain **19** (19 mg, 0,054 mmol, 91%). ^1^H NMR (300 MHz, CD_3_OD) δ 8.62 (s, 1H), 8.20 (d, *J* = 8.6 Hz, 2H), 7.89 (d, *J* = 8.6 Hz, 2H), 5.64 (d, *J* = 9.2 Hz, 1H), 4.14 (t, *J* = 9.4 Hz, 1H), 3.99 (d, *J* = 3.3 Hz, 1H), 3.92 (*t*_app_, *J* = 6.2 Hz, 1H), 3.79 (dd, *J* = 3.2 Hz, *J* = 9.7 Hz, 1H), 3.69 (m, 2H).

### 4.4 Synthesis of the Third Group of Analogs

#### 4.4.1 Synthesis of 1-(Azidomethyl)-4-nitrobenzene (**20**)

A solution of 1-(bromomethyl)-4-nitrobenzene (100 mg, 0.463 mmol, 1.0 equiv) in DMF (1,5 mL) was added dropwise in a solution of DMF (2 mL) and sodium azide (36.1 mg, 0.556 mmol, 1,2 equiv) in an ice bath. The mixture was stirred for 5 min. The reaction mixture changed its color to pale yellow. After completion, the solvent was removed under reduced pressure. The crude was partitioned between DCM and H_2_O and the aqueous phase was extracted 3× with DCM. The organic phase was dried over anhydrous MgSO_4_, filtered, and concentrated in vacuo. Product 1-(azidomethyl)-4-nitrobenzene (20) (673 mg; 3.778 mmol; 82% yield) was obtained as a yellow oil that required no further purification. ^1^H NMR (300 MHz, CDCl3) δ 8.24 (d, *J* = 6.9 Hz, 2H), 7.49 (d, *J* = 6.1 Hz, 2H), 4.50 (s, 2H).

#### 4.4.2 (4-(1-(4-Nitrobenzyl)-1*H*-1,2,3-triazol-4-yl)­phenyl)­methanol (**21**)

General procedure for CuAAC (4.2.4.). Azide: 1-(azidomethyl)-4-nitrobenzene (50 mg, 0.378 mmol). Product: solid (76 mg, 61% yield). ^1^H NMR (300 MHz, DMSO) δ 8.66 (d, *J* = 0.7 Hz, 1H), 8.31–8.20 (m, 2H), 7.85–7.76 (m, 2H), 7.62–7.52 (m, 2H), 7.38 (d, *J* = 8.0 Hz, 2H), 5.83 (s, 2H), 5.26 (t, *J* = 5.7 Hz, 1H), 4.52 (d, *J* = 5.7 Hz, 2H).

#### 4.4.3 3-(1-(4-Nitrobenzyl)-1*H*-1,2,3-triazol-4-yl)­pyridine (**22**)

General procedure for CuAAC (4.2.4.). Azide: 1-(azidomethyl)-4-nitrobenzene (50 mg, 0.378 mmol). Product: a white solid (71 mg, 67% yield). ^1^H NMR (300 MHz, DMSO) δ 9.04 (d, *J* = 2.0 Hz, 1H), 8.80 (s, 1H), 8.54 (dd, *J* = 4.7, 1.7 Hz, 1H), 8.29–8.17 (m, 3H), 7.58 (d, *J* = 8.8 Hz, 2H), 7.49 (dd, *J* = 8.5, 5.3 Hz, 1H), 5.86 (s, 2H).^13^C NMR (75 MHz, DMSO) δ 149.4, 147.7, 146.6, 144.4, 143.4, 133.2, 129.6, 124.4, 123.2, 52.7. HRMS (ESI) *m*/*z* calculated for C_13_H_12_N_6_O_2_ + H^+^: 282.09855, found: 282,0988.

#### 4.4.4 4-(1-(4-Nitrobenzyl)-1*H*-1,2,3-triazol-4-yl)­aniline (**23**)

General procedure for CuAAC (4.2.4.). Azide: 1-(azidomethyl)-4-nitrobenzene (50 mg, 0.378 mmol). Product: solid (70.5 mg, 61% yield). ^1^H NMR (300 MHz, DMSO) δ 8.38 (s, 1H), 8.24 (d, *J* = 8.8 Hz, 2H), 7.58 – 7.45 (m, 4H), 6.60 (d, *J* = 8.7 Hz, 2H), 5.78 (s, 2H), 5.26 (s, 1H). HRMS (ESI) *m*/*z* calculated for C_15_H_13_N_5_O_2_ + H^+^:296.11420, found: 296,1141.

#### 4.4.5 1-(4-Nitrobenzyl)-4-(4-nitrophenyl)-1*H*-1,2,3-triazole (**24**)

General procedure for CuAAC (4.2.4.). Azide: 1-(azidomethyl)-4-nitrobenzene (50 mg, 0.378 mmol). Product: solid (28.2 mg, 23% yield). ^1^H NMR (300 MHz, DMSO) δ 8.95 (s, 1H), 8.32 (d, *J* = 9.0 Hz, 2H), 8.26 (d, *J* = 8.8 Hz, 2H), 8.13 (d, *J* = 9.0 Hz, 2H), 7.59 (d, *J* = 8.8 Hz, 2H), 5.89 (s, 2H).

#### 4.4.6 4-(1-Methyl-1*H*-imidazol-5-yl)-1-(4-nitrobenzyl)-1*H*-1,2,3-triazole (**25**)

General procedure for CuAAC (4.2.4.). Azide: 1-(azidomethyl)-4-nitrobenzene (40 mg, 0.302 mmol). Product: solid (65 mg, 75% yield). ^1^H NMR (300 MHz, DMSO) δ 8.57 (s, 1H), 8.30–8.11 (m, 3H), 7.63–7.49 (m, 3H), 5.85 (s, 2H), 3.81 (s, 3H). ^13^C NMR (75 MHz, DMSO) δ 147.7, 143.6, 129.5, 124.4, 123.0, 52.5. HRMS (ESI) *m*/*z* calculated for C_13_H_12_N_6_O_2_ + H^+^: 285.10945, found: 285,1084.

#### 4.4.7 4-(3-Methoxyphenyl)-1-(4-nitrobenzyl)-1*H*-1,2,3-triazole (**26**)

General procedure for CuAAC (4.2.4.). Azide: 1-(azidomethyl)-4-nitrobenzene (50 mg, 0.378 mmol). Product: solid (67 mg, 57% yield). ^**1**^ **H NMR** (300 MHz, DMSO) δ 8.72 (s, 1H), 8.26 (d, 2H), 7.57 (d, 2H), 7.46–7.32 (m, 3H), 6.91 (ddd, *J* = 8.1, 2.6, 1.2 Hz, 1H), 5.84 (s, 2H), 3.80 (s, 3H).

#### 4.4.8 (2-(1-(4-Nitrobenzyl)-1*H*-1,2,3-triazol-4-yl)­phenyl)­methanol (**27**)

General procedure for CuAAC (4.2.4.). Azide: 1-(azidomethyl)-4-nitrobenzene (50 mg, 0.378 mmol). Product: solid (inedited) (59 mg, 47% yield). ^1^H NMR (300 MHz, DMSO) δ 8.57 (s, 1H), 8.26 (d, *J* = 8.7 Hz, 2H), 7.82–7.72 (m, 1H), 7.62–7.52 (m, 3H), 7.41–7.33 (m, 2H), 5.87 (s, 2H), 5.34 (t, *J* = 5.6 Hz, 1H), 4.61 (d, *J* = 5.3 Hz, 2H). ^13^C NMR (75 MHz, DMSO) δ 147.7, 146.0, 143.7, 139.4, 129.5, 128.8, 128.7, 128.6, 127.8, 124.5, 124.4, 62.0, 52.6. HRMS (ESI) *m*/*z* calculated for C_16_H_14_N_4_O_3_ + H^+^: 311.11387, found: 311.1132.

#### 4.4.9 4-(3,5-Dimethoxyphenyl)-1-(4-nitrobenzyl)-1*H*-1,2,3-triazole (**28**)

General procedure for CuAAC (4.2.4.). Azide: 1-(azidomethyl)-4-nitrobenzene (50 mg, 0.378 mmol). Product: solid (inedited) (77 mg, 57% yield). ^1^H NMR (300 MHz, DMSO) δ 8.72 (s, 1H), 8.26 (d, *J* = 8.7 Hz, 2H), 7.56 (d, *J* = 8.7 Hz, 2H), 7.02 (d, *J* = 2.3 Hz, 2H), 6.47 (t, *J* = 2.3 Hz, 1H), 5.83 (s, 2H), 3.78 (s, 6H). ^13^C NMR (75 MHz, DMSO) δ 161.3, 147.7, 147.2, 143.8, 132.8, 129.5, 124.4, 122.9, 103.6, 100.5, 55.7, 52.6. HRMS (ESI) *m*/*z* calculated for C_17_H_16_N_4_O_4_ + H^+^: 341.12443, found: 341,1245.

### 4.5 Biological Assays

#### 4.5.1 Antitrypanosomal Screening

Anti-parasitic assays were conducted following established protocols outlined in earlier literature.^67^ In brief, for assessing anti-T. cruzi activity, the Tulahuen CL2 β-galactosidase strain (nifurtimox-sensitive) was employed and maintained on MRC-5_SV2_ (human lung fibroblast). A total of 4 × 10^3^ cells were infected with 4 × 10^4^ parasites per well. The synthesized analogs were tested at concentrations ranging from 1.56 to 50.00 μM. Parasite burdens were evaluated after the addition of the substrate CPRG (chlorophenol red ß-d-galactopyranoside). The change in color was measured spectrophotometrically at 540 nm after 4 h of incubation at 37°C. In all assays, parasite growth was compared to untreated-infected controls (100% growth) and noninfected controls (0% growth). Results were expressed as the percentage of parasite reduction at different drug concentrations and used to calculate IC_50_ values from the dose–response curves.^68^

#### 4.5.2 Preliminary Cytotoxicity Assays

MRC-5_SV2_ cell cytotoxicity was assessed following the methodology outlined in earlier literature.^67^ Briefly, cells at a concentration of 1.5 × 10^5^ cells/mL were cultured with compound dilutions at 37 °C and 5% CO_2_. Cell growth was compared to untreated-control wells (100% cell growth) and medium-control wells (0% cell growth). After 3 days of incubation, cell viability was fluorometrically assessed following the addition of 50-μL resazurin per well. Fluorescence was measured after 4 h at 37 °C (λ_ex_ 550 nm, λ_em_ 590 nm). The results were expressed as the percentage reduction in cell growth/viability compared to control wells, and an IC_50_ value was determined.^68^

#### 4.5.3 Cells and Parasite Maintenance^37,40^

The LLC-MK2 (ATCC CCL-7) cell line of epithelial origin, and C2C12 (ATCC CRL-1772) myoblast cells, derived from murine myoblasts, were maintained in Roswell Park Memorial Institute-RPMI 1640 medium supplemented with l-glutamine, 25 mM HEPES (Corning), 10% (v/v) fetal bovine serum (FBS), and gentamicin (1 μL/mL). The culture medium was replaced every 2 days, and the cells were incubated at 37 °C in 5% CO_2_. Once the cells reached 80% confluence, were detached using Trypsin-EDTA and prepared in the subsequent experiments.

Trypomastigote forms of the T. cruzi Colombian strain were obtained from BALB/c mice and maintained in LLC-MK2 culture cells with Roswell Park Memorial Institute-RPMI 1640 medium with l-glutamine and 25 mM HEPES (Corning) supplemented with fetal bovine serum at 5% (v/v) and 1 μL/mL gentamicin The medium was refreshed every 2 days and the cultures were incubated at 37 °C in 5% CO_2_. Parasites were collected for experiments upon completion of replication cycle and host cell lysis.

#### 4.5.4 Cellular Viability^37,40^

The LLC-MK2 and C2C12 line cells were plated in 96-well culture plates at a concentration of 1 × 10^6^ cells/mL. Post cell adhesion, the supernatant was discarded to remove nonadherent cells, which were exposed to treatments using compound **16** and **19** or BZN in serial concentrations (**16** and **19** ranging from 80 to 0.08 μM; BZN ranging from 66.2 to 0.12 μM). Following 24 h of treatment, 10 μL of 5 mg/mL of MTT (3-(4,5-dimethylthiazol-2-yl)-2,5-diphenyltetrazolium bromide) was added per well, and absorbance was evaluated in the Eon Microplate Spectrophotometer Reader (BioTek) at 570–690 nm. CC_50_ were calculated by Nonlinear regression curves (log­[inhibitor] vs normalized response).

#### 4.5.5 Antiamastigote Activity^37,40^

For the antiamastigote activity evaluation, LLC-MK2 and C2C12 cells (1 × 10^4^ cells/mL) were plated in 96-well culture plates at a concentration of 1 × 10^4^ cells/mL and incubated during 4 h for cellular adhesion at 37 °C in 5% CO_2_. After incubation time, cells were infected with trypomastigotes forms (Colombian strain) using MOI 20:1 overnight. After this incubation, extracellular parasites were removed, and the cells were maintained for 48 h at 37 °C with 5% CO_2_ to allow differentiation and multiplication of amastigotes. After amastigotes growth, cells were exposed to treatments using compound **16** and **19** or BZN in serial concentrations (**16** and **19** ranging from 80 to 0.08 μM; BZN ranging from 66.2 to 0.12 μM) during 48 h of treatment. After the treatment, the plate was fixed with 50 μL/well of methanol for the immunofluorescence protocol.

For antiamastigote activity of pretreated cells LLC-MK2 and C2C12 cells were plated in 96-well culture plates at a concentration of 1 × 10^4^ cells/mL and incubated during 4 h for cellular adhesion at 37 °C in 5% CO_2_. After this incubation, cells were exposed to treatments using compound **16** and **19** or BZN in serial concentrations (**16** and **19** ranging from 80 to 0.08 μM; BZN ranging from 66.2 to 0.12 μM) during 48 h of treatment. After treatment time, cells were infected with trypomastigotes forms (Colombian strain) using MOI 20:1 overnight and the plate was fixed with 50 μL/well of methanol for the immunofluorescence protocol.

For interaction evaluation of the **16** and **19** compounds and BZN, LLC-MK2 and C2C12 cells (1 × 10^4^ cells/mL) were plated in 96-well culture plates at a concentration of 1 × 10^4^ cells/mL and incubated during 4 h for cellular adhesion at 37 °C in 5% CO_2_. After incubation time, cells were infected with trypomastigotes forms (Colombian strain) using MOI 20:1 overnight. After this incubation, extracellular parasites were removed, and the cells were maintained for 48 h at 37 °C with 5% CO_2_ to allow differentiation and multiplication of amastigotes. After amastigotes growth, cells were exposed to treatments using the combination of compound **16** and **19** (at concentrations ranging from 0.25 to 0.06 μM) with BZN (BZN, 3.8 μM) during 48 h of treatment. After the treatment, the plate was fixed with 50 μL/well of methanol for the immunofluorescence protocol. The interactions of compounds **16** and **19** with benznidazole (BZN) were evaluated using isobologram analysis with the CompuSyn software (CompuSyn, Inc.). The analyses were conducted with isolated concentrations of **16** and **19** ranging from 80 to 0.08 μM, and BZN ranging from 66.2 to 0.12 μM. The combinations utilized doses of **16** and **19** at 0.25, 0.12, and 0.06 μM, combined with a fixed dose of BZN at 3.8 μM. The results were expressed by presenting different effective doses and interpreting the interactions. The combination index (CI) values were interpreted as follows: CI < 1 indicates synergy, CI = 1 indicates additivity, and CI > 1 indicates antagonism.

#### 4.5.6 Immunofluorescence^37,40^

In the infection plate each well was added 50 μL of cold methanol for cell fixation. The plate was kept at −20 °C for 1 h. After this period, the wells were blocked with 50 μL of PBS 1X-Saponin 0.1 + 10% rabbit serum for 10 min at room temperature. Following, the wells were washed four times with PBS 1X-Saponin 0.1% and 50 μL of the same solution was added to each well, incubating for 20 min at room temperature. Subsequently, 50 μL of PBS 1X-Saponin 0.1% + *anti*-T. cruzi antibody (from serum of infected patients) at a 1:10 dilution was added, and the plate was incubated for 1 h at room temperature. The wells were then washed four times with PBS 1X-Saponin 0.1%, and 50 μL of PBS 1X-Saponin 0.1% + antihuman IgG-FITC at a 1:200 dilution was added. The plate was incubated wrapped in aluminum foil for 30 min. After incubation, the plate was washed four times with PBS 1X-Saponin 0.1%, and 50 μL of PBS 1X was added to all wells after the final wash. For nuclear staining, the plate, already stained with FITC, was washed three times with PBS 1X, and 200 μL of PBS 1X + 1 μL of iodide per well was added. After staining, the wells were washed again, and 100 μL of PBS 1X was added before imaging.

The plate was analyzed in the EVOS fluorescence microscope for image acquisition and fluorescence identification, in which the *anti*-IgG-FITC stains parasite′s membrane in green and iodide stains nuclei in red. On each condition of treatment, 200 cells of 3 wells were evaluated as to the number of infected cells and the number of parasites per cell for calculating the index of infection the IC_50_ were calculated by Nonlinear regression curves (log­[inhibitor] vs normalized response). Selective index was calculated by dividing CC_50_ per IC_50_.

#### 4.5.7 T. cruzi Nitroreductase Activity Assay

*Tc*NTR72 was expressed, purified, and its activity was tested following previously described protocol.^49^ Enzyme activity was measured by monitoring the oxidation of NADH at 340 nm (ε = 6.220 M^–1^ cm^–1^) in a 96-well UV-transparent plate over a period of 120 s using a SpectraMax Plus 384 Microplate Reader (Molecular Devices) at 25 ± 1 °C. The reaction mixture was prepared by combining freshly prepared solutions with final concentrations of 50 mM Tris pH 7.5, 0.1% Triton X-100, 50 μM NADH, and the compounds at various concentrations (100, 50, 25, and 12.5 μM). This mixture was incubated at room temperature for 5 min. The oxidation reaction was initiated by adding the reaction mixture to 10 μL of a 200 μg/mL solution of *Tc*NTR72 in 50 mM Tris pH 8.5 and 0.1% Triton X-100, reaching a final concentration of 376 nM (10 μg/mL). All assays were performed in triplicate for each measurement and for each concentration. Benznidazole was used as a control.

#### 4.5.8 Fluorimetric TcTS Inhibition

A truncated recombinant TcTs, containing the enzyme’s catalytic N-terminal domain, was used. The production and purification of TcTs were performed following previously described protocols.^69^ The continuous fluorimetric TcTS assay was conducted in 96-well plates containing phosphate buffer solution at pH 7.4 (25 μL), recombinant enzyme solution (25 μL) and inhibitor solution (25 μL).^56^ After incubation of the mixture for 10 min at 25 °C, in the presence of variable concentrations of the compound **19** (4 mM to 0.03125 mM) in a 0.5% of DMSO aqueous solution, the MuNANA substrate (*K*_m_ = 0.68 mM; 25 μL of a 0.4 mM solution) was added and the fluorescence of the methylumbelliferone was measured during 10 min, with excitation and emission wavelengths of 360 and 460 nm, respectively. The enzyme activity was measured by calculating the slope of the curve for each concentration of the test compound. Pyridoxal phosphate was used as the inhibition control at a concentration of 1 mM. The IC_50_ value was determined using the log­(inhibitor) vs normalized response—variable slope model in GraphPad Prism version 10.0.0 for Windows, GraphPad Software, Boston, Massachusetts (www.graphpad.com).

## Supplementary Material



## Data Availability

All data supporting the findings of this study are provided within the article and the Supporting Information. Further details are available upon request from the corresponding author.

## Notes

Structural representations, predicted physicochemical properties of hit **1** derivatives (Table S1); synthetic yields (Table S2); and detailed characterization data. Spectroscopic analyses such as ^1^H NMR, ^13^C NMR, HRMS, DEPT 135, COSY, HSQC, and infrared spectra for synthesized compounds are included, along with chromatographic purity assessments (PDF)
