# Supplementary material for: Synthesis and Biological Evaluation of 4‑(4-Nitrophenyl)‑1H‑1,2,3-triazole Derivatives as Antitrypanosomal Agents
Source: ACS Omega. 2025 May 13;10(20):20299–314. doi: 10.1021/acsomega.4c11645 (PMC12120661; doi:10.1021/acsomega.4c11645)

Synthesis and biological evaluation of 4-(4-nitrophenyl)-1H-1,2,3-triazole derivatives as antitrypanosomal agents

Filipe Canto Oliveira[a], Luís Otávio Bunhotto Zamoner[a], Marcelo Dias Baruffi[b], Beatriz Silveira Augusto[c,d], Miguel de Menezes Vaidergorn[c,d], Maria Cristina Nonato[c,d] Thainá Silva Bologna[e], Marcos Vinicius da Silva[e] and Ivone Carvalho[a*]

[a] *Department of Pharmaceutical Sciences, School of Pharmaceutical Sciences of Ribeirão Preto, University of São Paulo, Av. Café s/n, Ribeirão Preto, SP 14040-930, Brazil.*
[b] *Department of Clinical, Toxicological and Bromatological Analysis, School of Pharmaceutical Sciences of Ribeirão Preto, University of São Paulo, Av. Café s/n, Ribeirão Preto, SP 14040-930, Brazil.*
[c] *Center for the Research and Advancement in Fragments and molecular Targets (CRAFT), School of Pharmaceutical Sciences at Ribeirao Preto, University of São Paulo, Ribeirão Preto 14040-903, SP, Brazil*
[d] *Protein Crystallography Laboratory, Department of Biomolecular Sciences, School of Pharmaceutical Sciences at Ribeirao Preto, University of São Paulo, Ribeirão Preto 14040-903, SP, Brazil*
[e] *Department of Microbiology, Immunology, and Parasitology, Federal University of Triangulo Mineiro, Av. Frei Paulino 30, Uberaba, MG, 38025-180, Brazil.*
[*] *Corresponding author. Tel.: +55 16 3602 4709. E-mail address: carronal@usp.br (I. Carvalho)*

Supplementary data

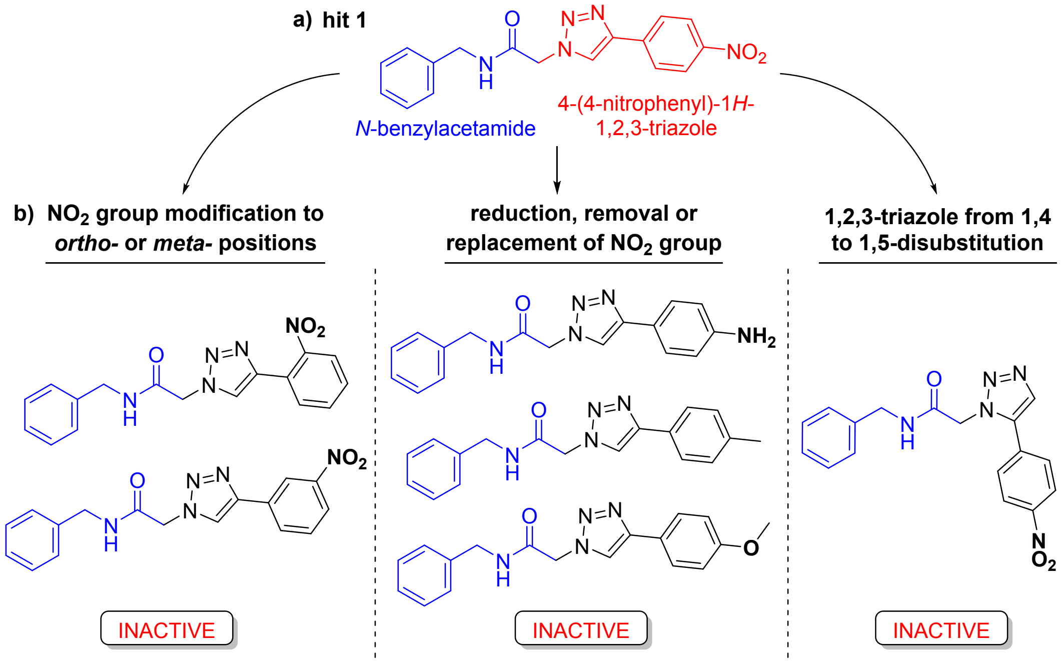

Fig. S1. ***a)*** *N-benzylacetamide and 4-(4-nitrophenyl)-1H-1,2,3-triazole scaffolds of prototype **1** **b)** Previous structural modifications made by our group in hit **1** that led to loss of trypanocidal activity.*

Table S1. Summary of the predicted key properties for hit **1** analogs*.

Cpd.	MW (g/mol)	Log $P_{o/w}$ [a]	Water Solubility [b]	GI absorption	BBB permeant	Lipinski[c]
**BZN**	260.25	0.49	soluble	high	no	yes
hit **1**	337.33	1.41	moderately soluble	high	no	yes
Series 1						
**10**	338.32	0.75	moderately soluble	high	no	yes
**11**	338.32	0.71	moderately soluble	high	no	yes
**12**	353.33	1.05	moderately soluble	high	no	yes
**13**	362.34	1.24	moderately soluble	high	no	yes
Series 2						
**15**	305.29	2.00	moderately soluble	high	no	yes
**16**	534.47	0.70	soluble	low	no	no
**17**	248.19	0.22	soluble	high	no	yes
**18**	324.29	1.66	moderately soluble	high	no	yes
**19**	352.30	-1.29	soluble	low	no	yes
Series 3						
**21**	310.31	1.62	moderately soluble	high	no	yes
**22**	281.27	1.51	moderately soluble	high	no	yes
**23**	295.30	1.51	moderately soluble	high	no	yes
**24**	325.28	1.50	moderately soluble	high	no	yes
**25**	284.27	0.89	soluble	high	no	yes
**26**	310.31	2.05	moderately soluble	high	no	yes
**27**	310.31	1.60	moderately soluble	high	no	yes
**28**	340.33	2.09	moderately soluble	high	no	yes

*Predictions generated by SwissADME website. [a] consensus Log $P_{o/w}$. [b] calculated by SILICOS-IT fragmental method. [c] Lipinski's rule of five.

Table S2. Yield of compounds synthesized.

Compound	Yield %	Compound	Yield %
**2**	95	**16**	86
**3**	74	**17**	75
**4**	85	**18**	90
**5**	32	**19**	91
**6**	62	**20**	82
**7**	49	**21**	61
**8**	80	**22**	67
**9**	95	**23**	60
**10**	13	**24**	23
**11**	97	**25**	75
**12**	96	**26**	57
**13**	23	**27**	47
**14**	30	**28**	57
**15**	61		

Characterization and purification of first group of analogs

Fig S2. ^{1}H NMR of compound **4** ($CDCl_3$, 300 MHz)

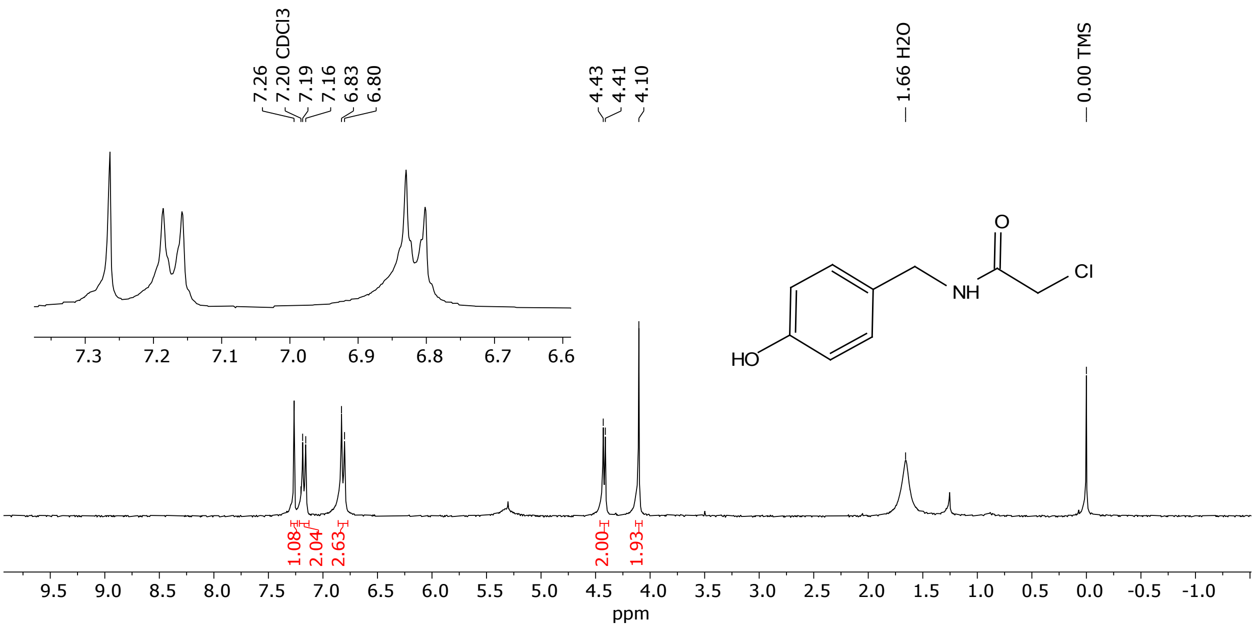

Fig S3. ^{1}H NMR of compound **8** ($CDCl_3$, 300 MHz)

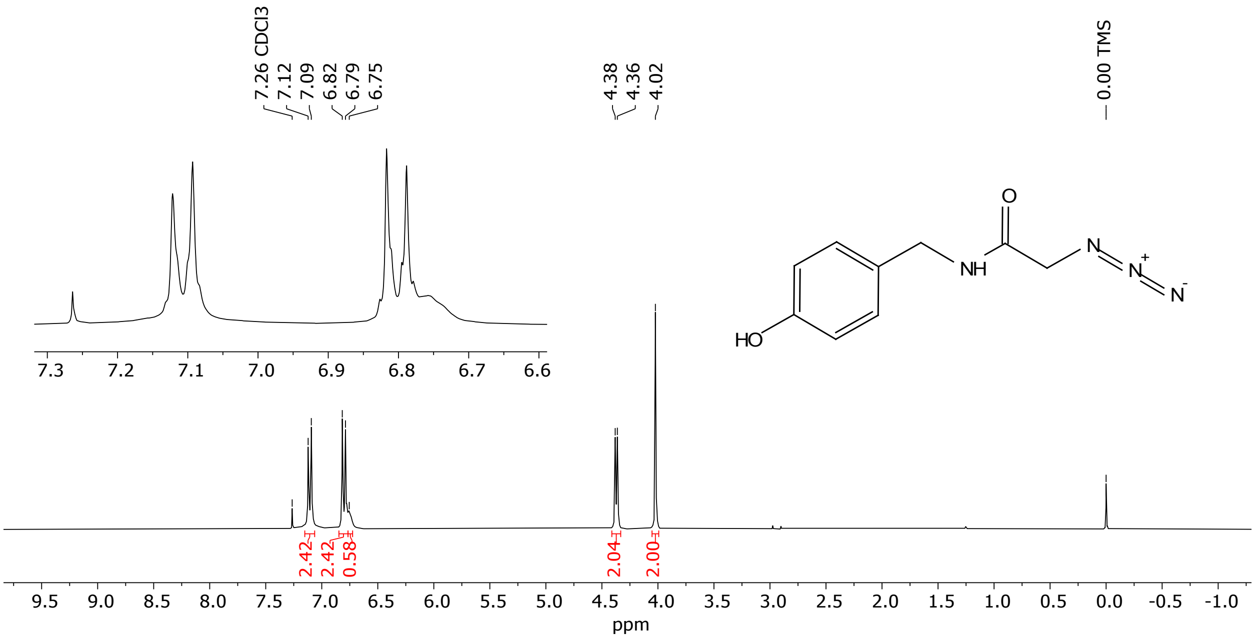

Fig S4. ^{1}H NMR of compound **12** (DMSO-d6, 300 MHz)

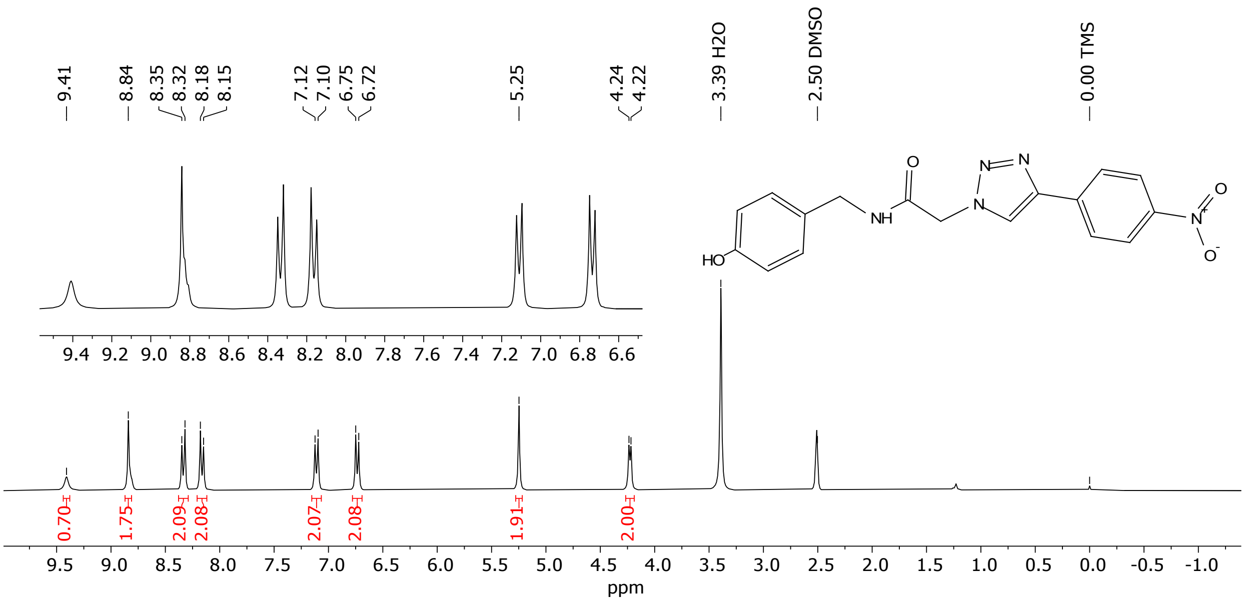

Fig S5. ^{13}C NMR of compound **12** (75 MHz, DMSO-d6)

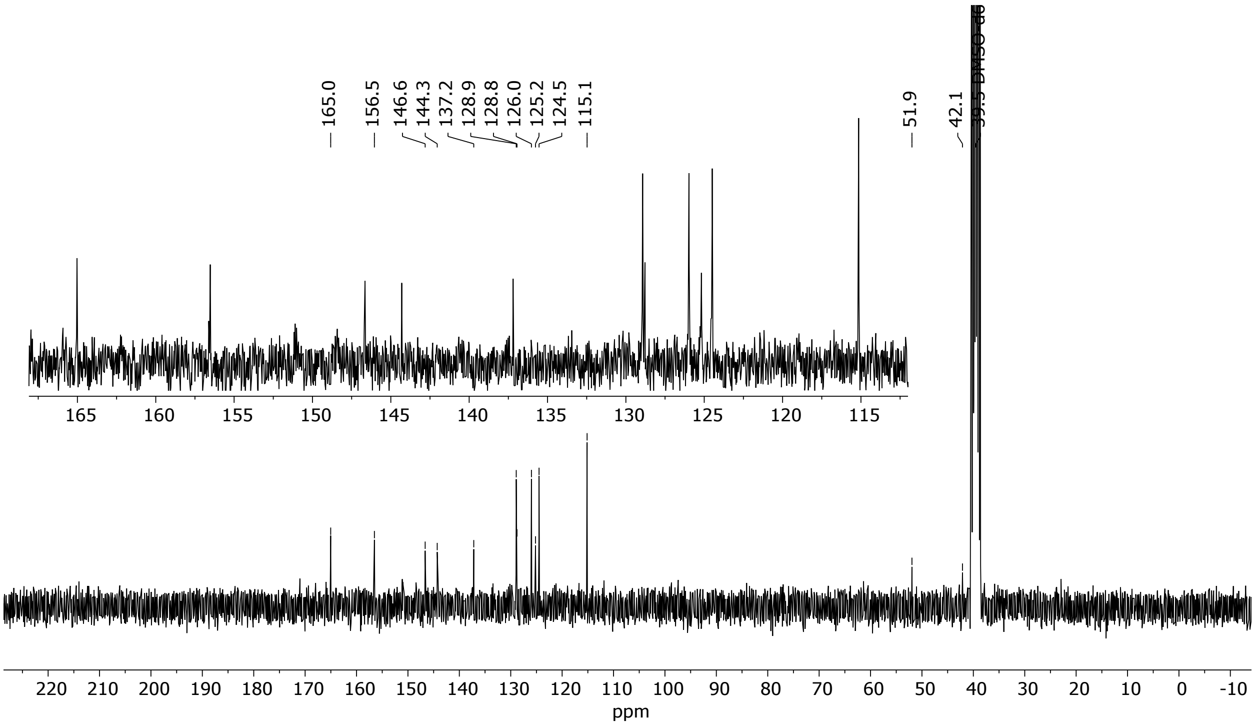

Fig S6. HRMS of compound **12**

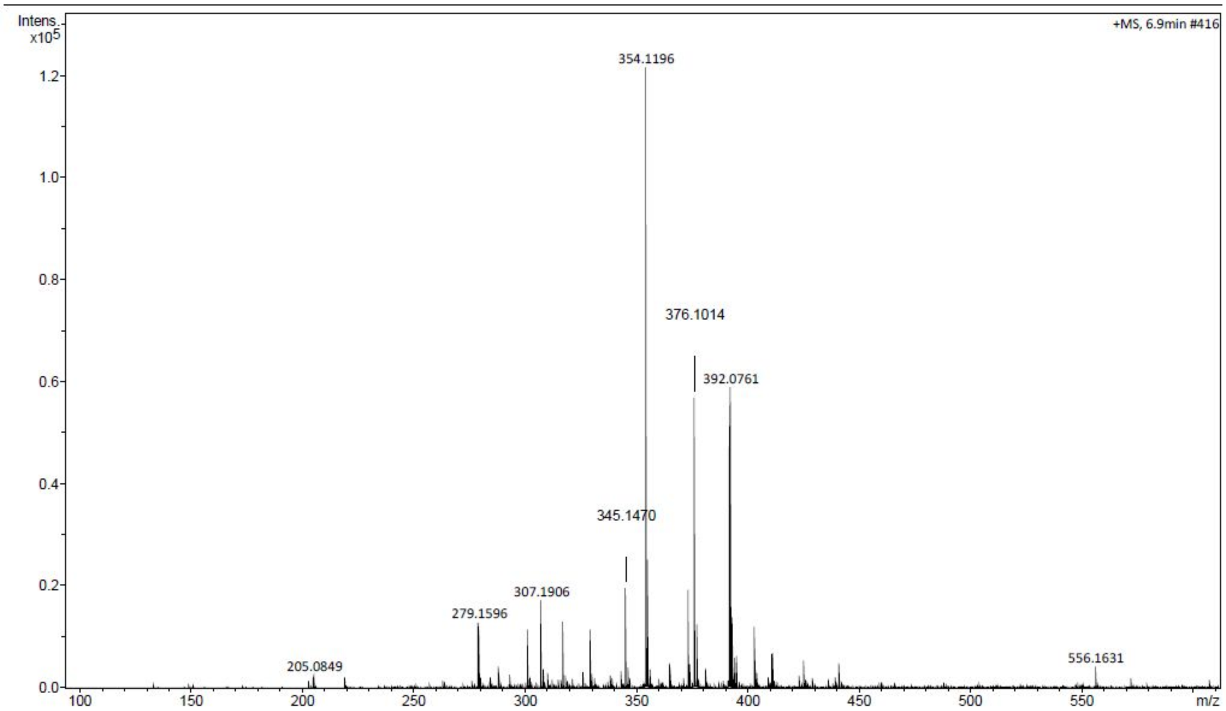

Fig S7. Chromatographic purity analysis of compound **12**

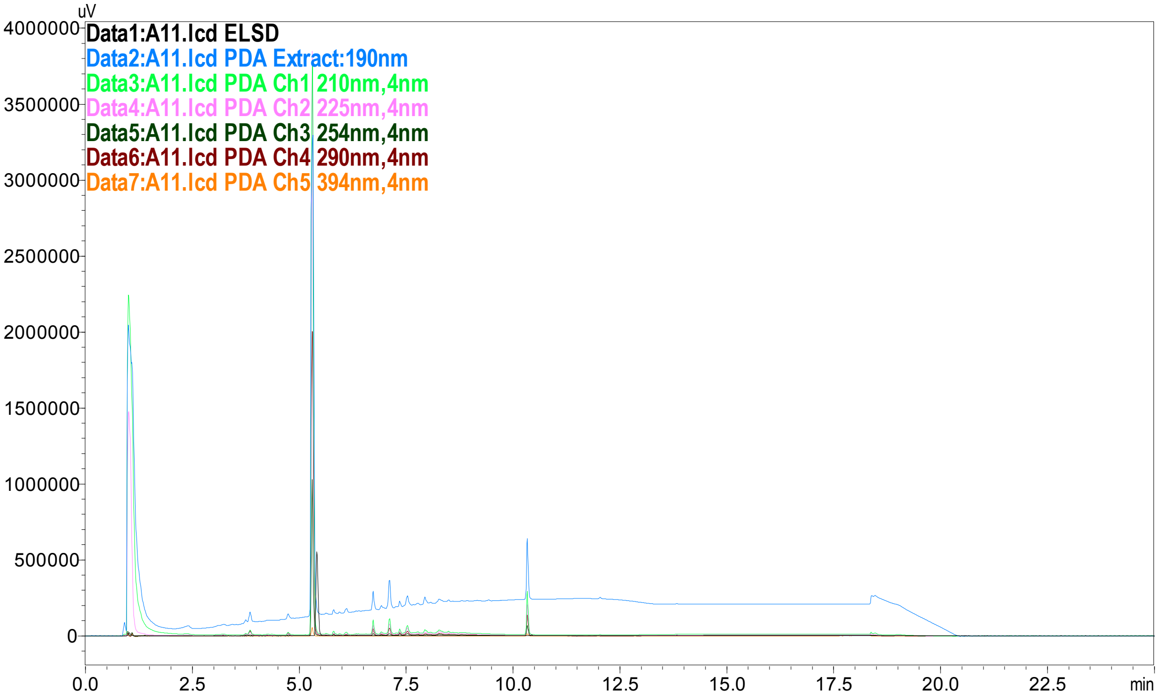

Fig S8. ^{1}H NMR of compound **3** (DMSO-d6, 300 MHz)

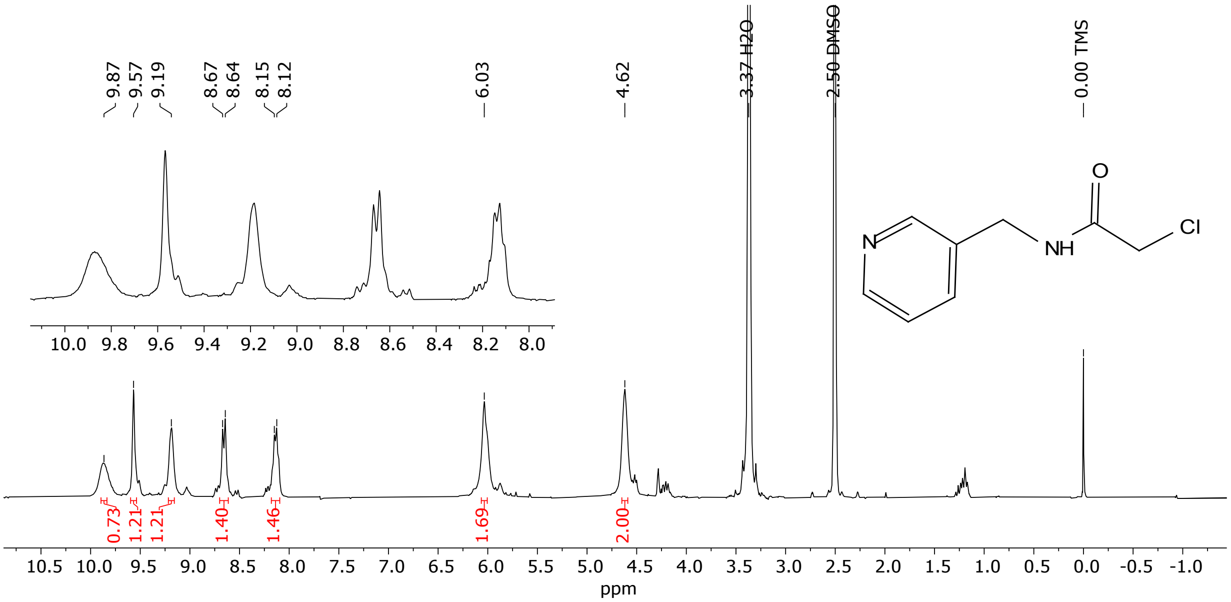

Fig S9. ^{1}H NMR of compound **7** ($CDCl_3$, 300 MHz)

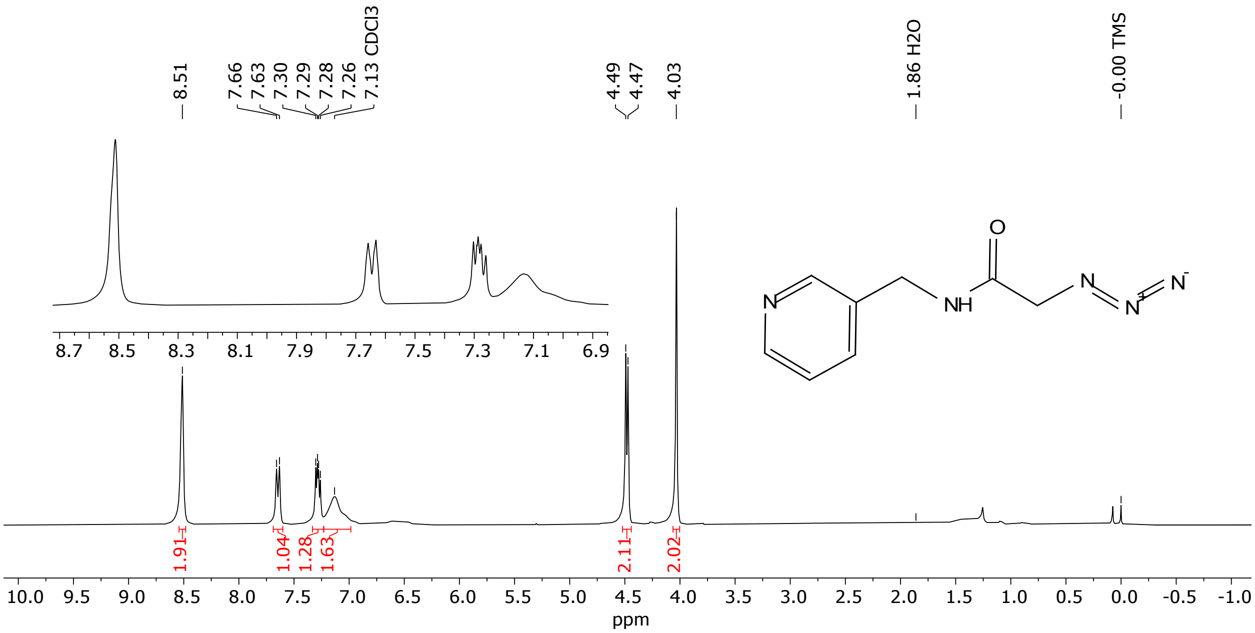

Fig S10. ^{1}H NMR of compound **11** (DMSO-d6, 300 MHz)

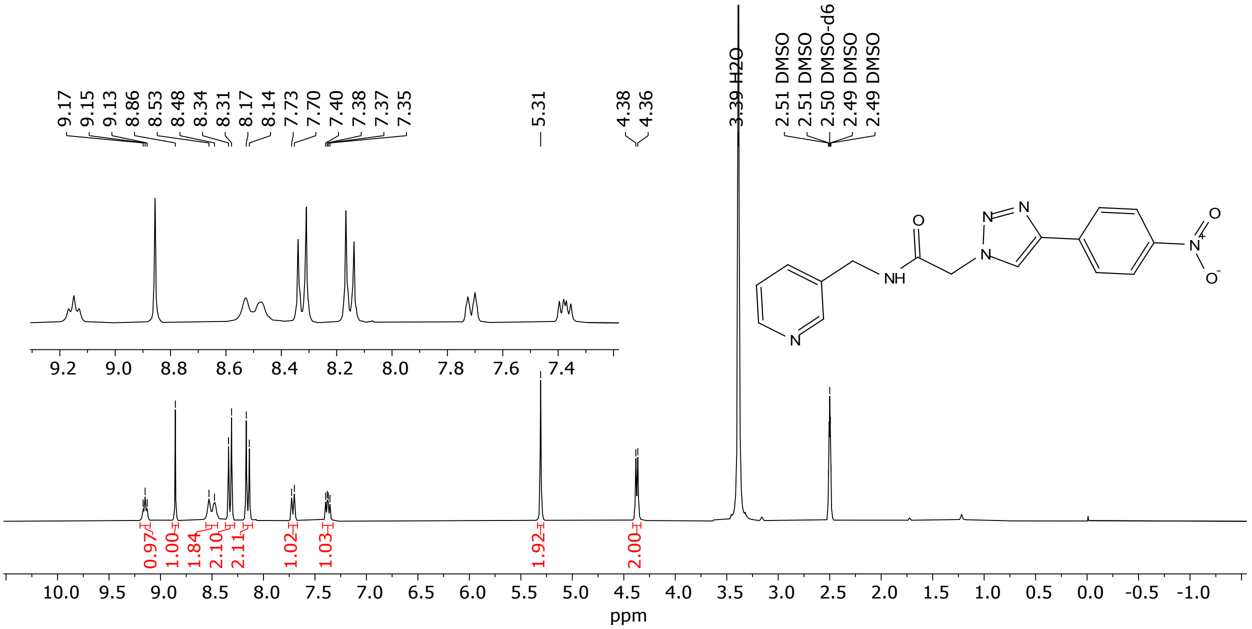

Fig S11. ^{13}C NMR and DEPT 135 of compound **11** (75 MHz, DMSO-d6)

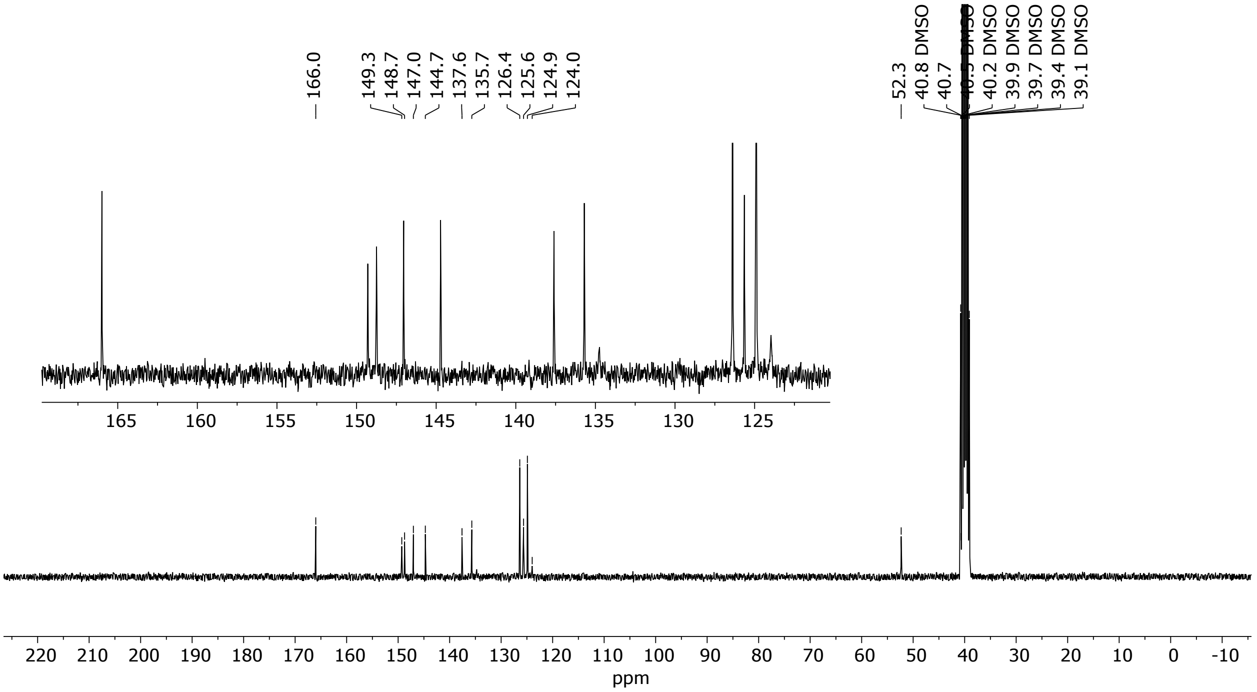

Fig S12. DEPT 135 of compound **11** (75 MHz, DMSO-d6)

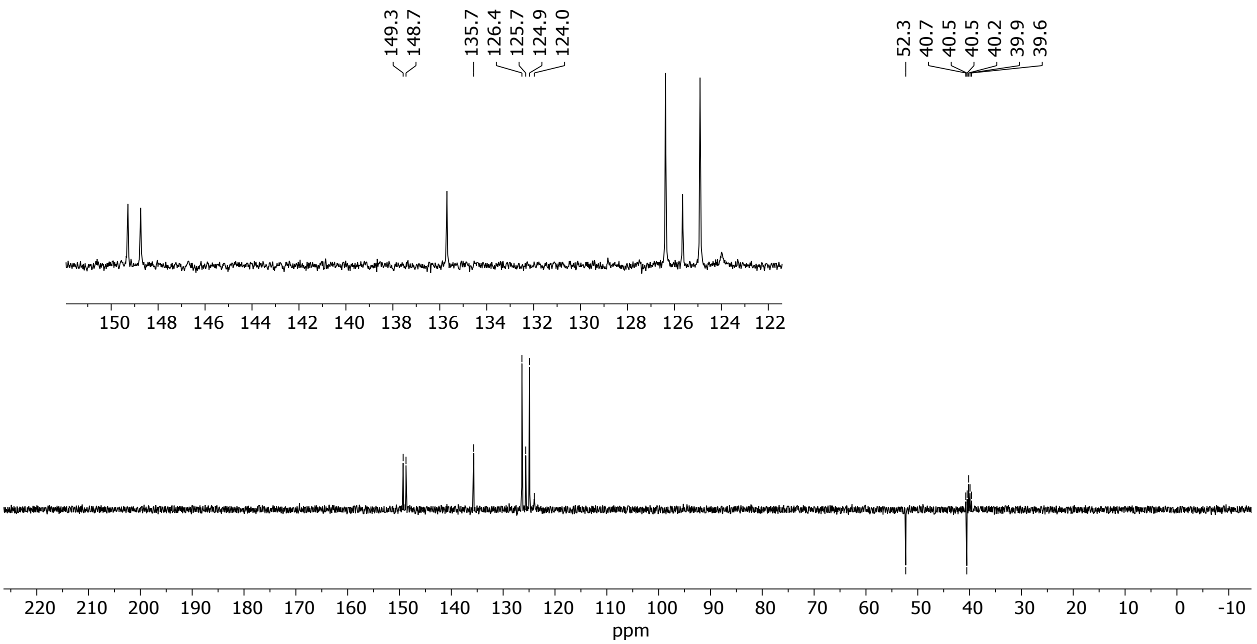

Fig S13. HRMS of compound **11**

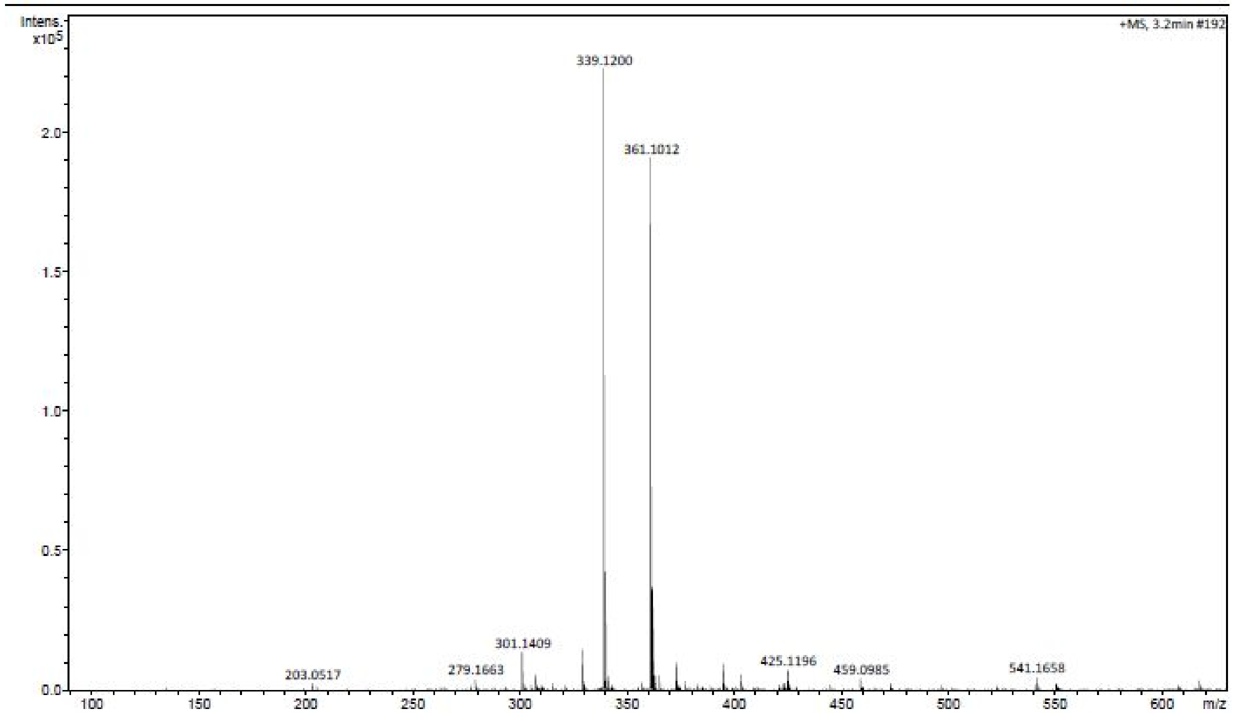

Fig S14. Chromatographic purity analysis of compound **11**

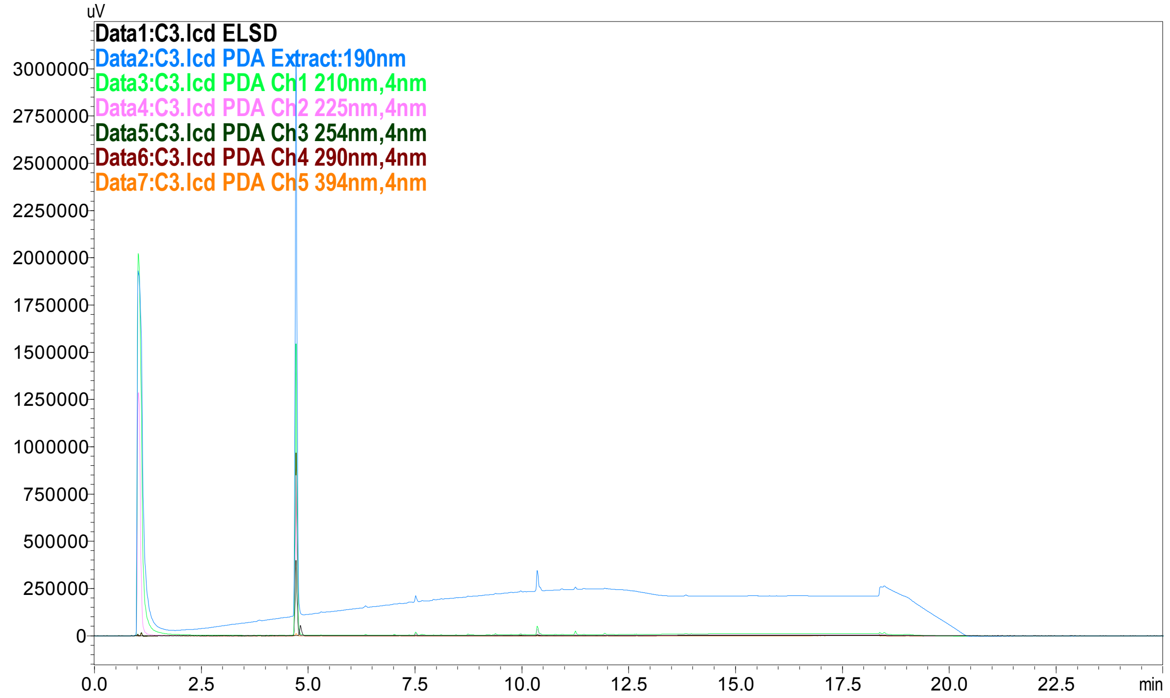

Fig S15. ^{1}H NMR of compound **2** ($CDCl_3$, 300 MHz)

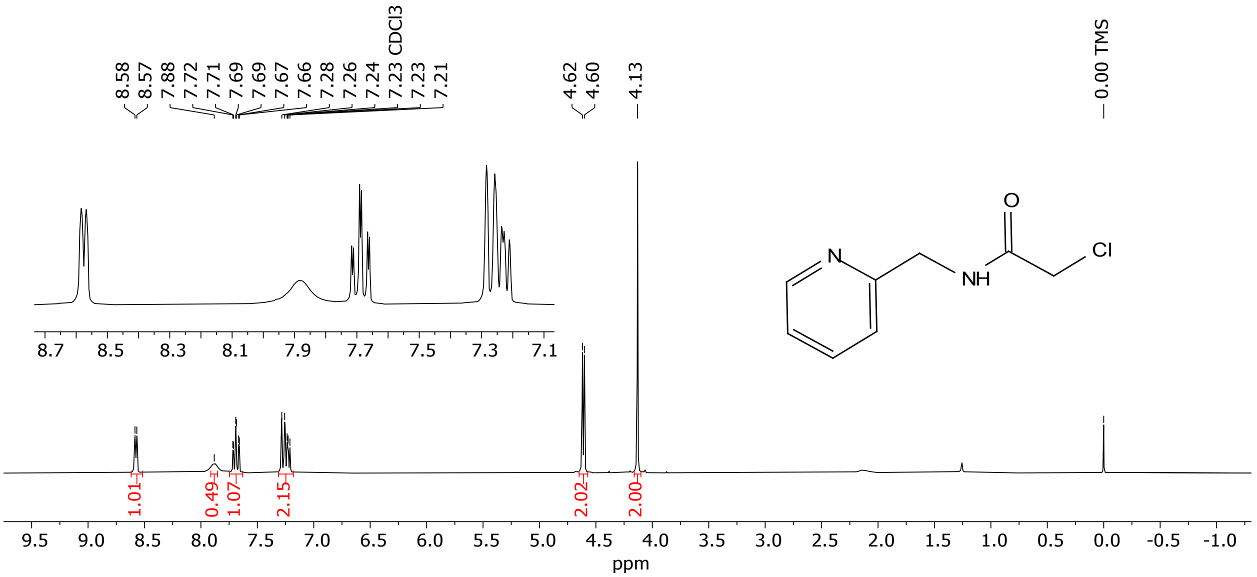

Fig S16. Infrared spectrum of compound **6**

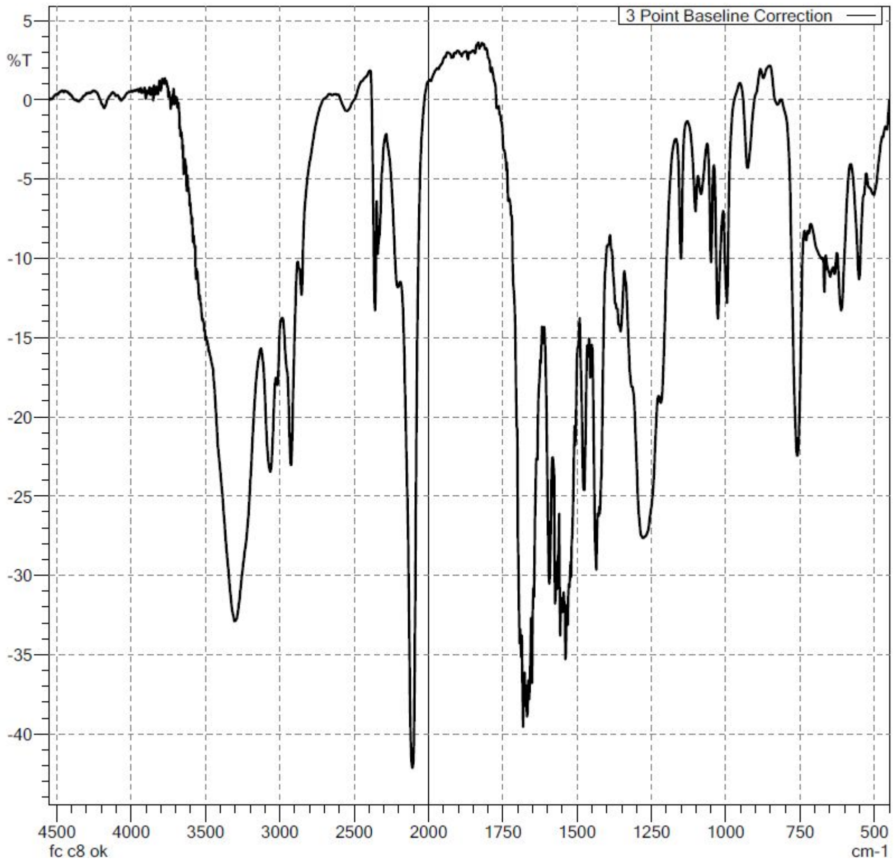

Fig S17. ^{1}H NMR of compound **10** (DMSO-d6, 300 MHz)

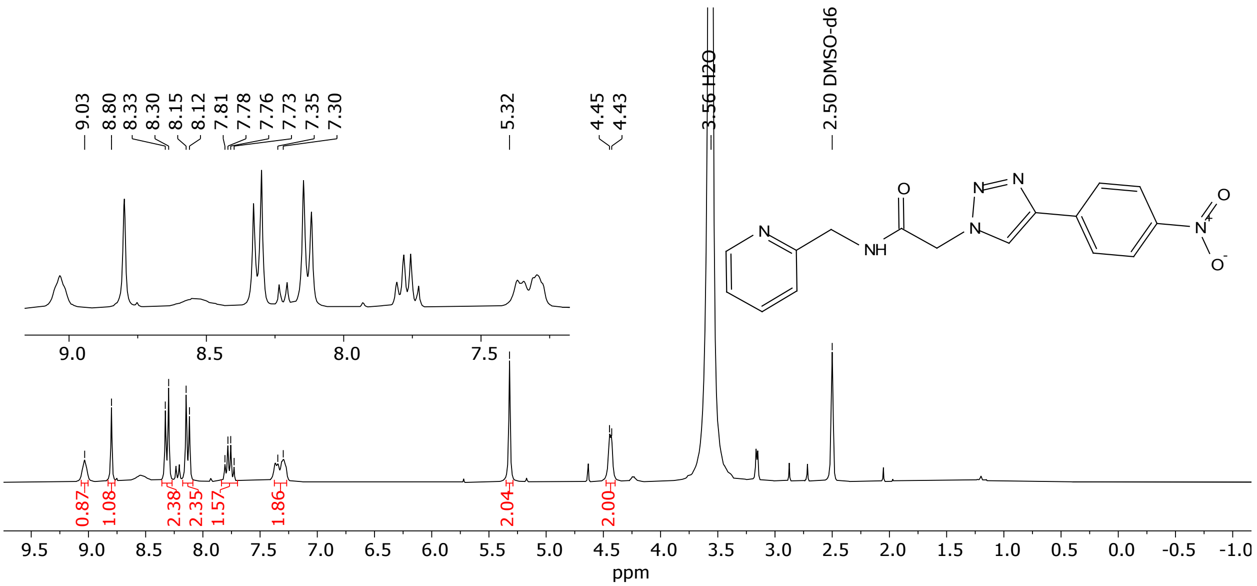

Fig S18. ^{13}C NMR of compound **10** (151 MHz, DMSO)

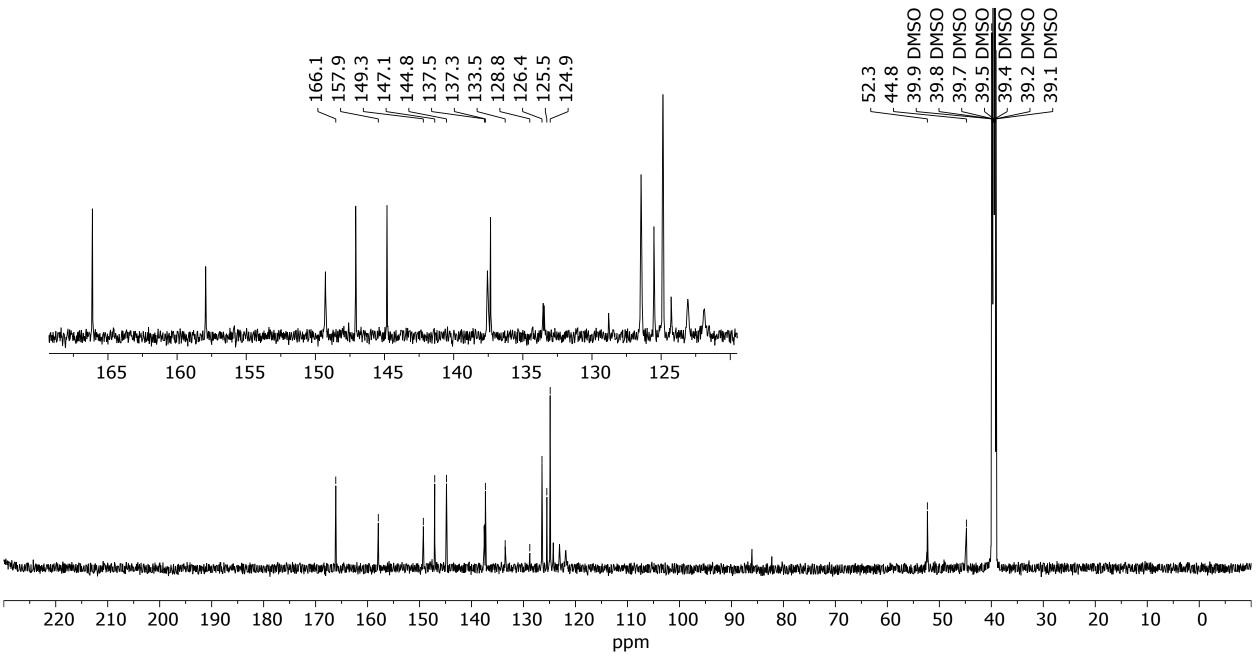

Fig S19. HRMS of compound **10**

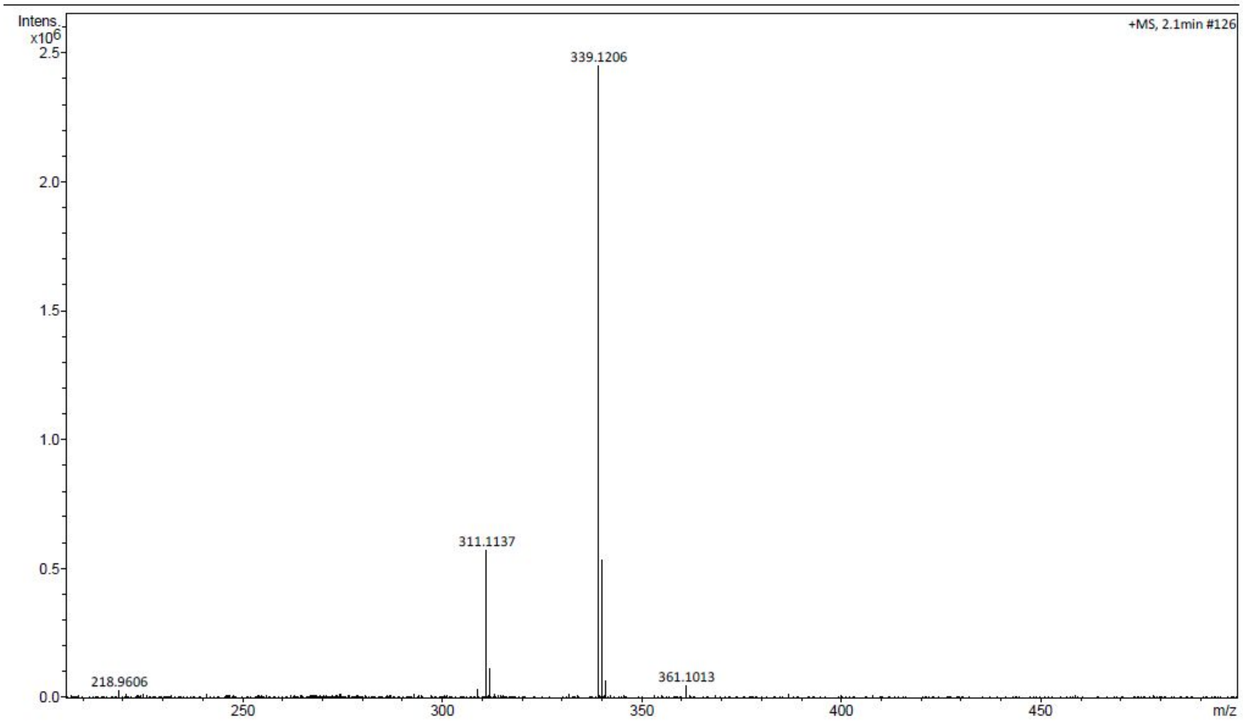

Fig S20. Chromatographic purity analysis of compound **10**

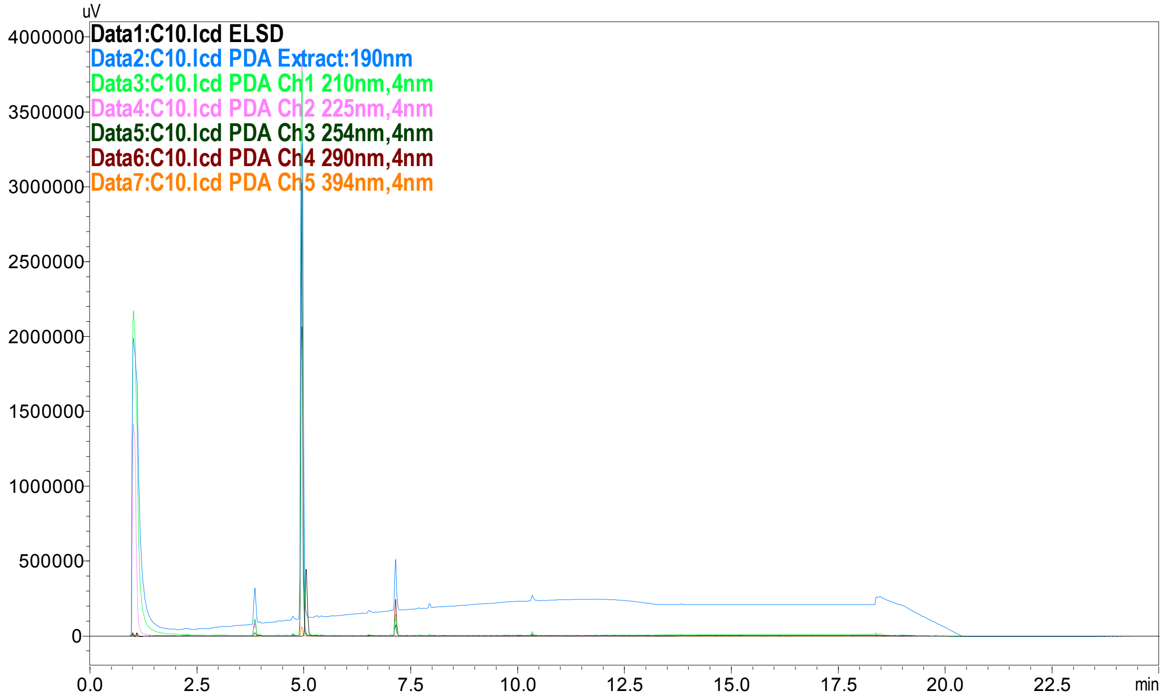

Fig S21. ^{1}H NMR of compound **5** (300 MHz, DMSO-d6)

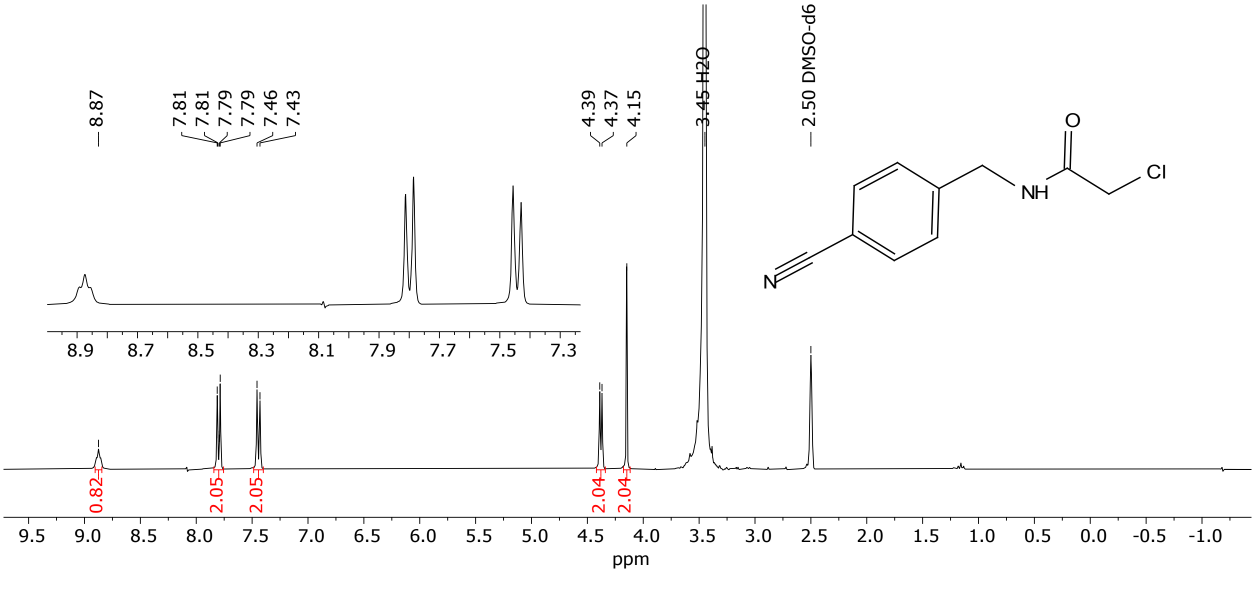

Fig S22. ^{1}H NMR of compound **9** (300 MHz, DMSO-d6)

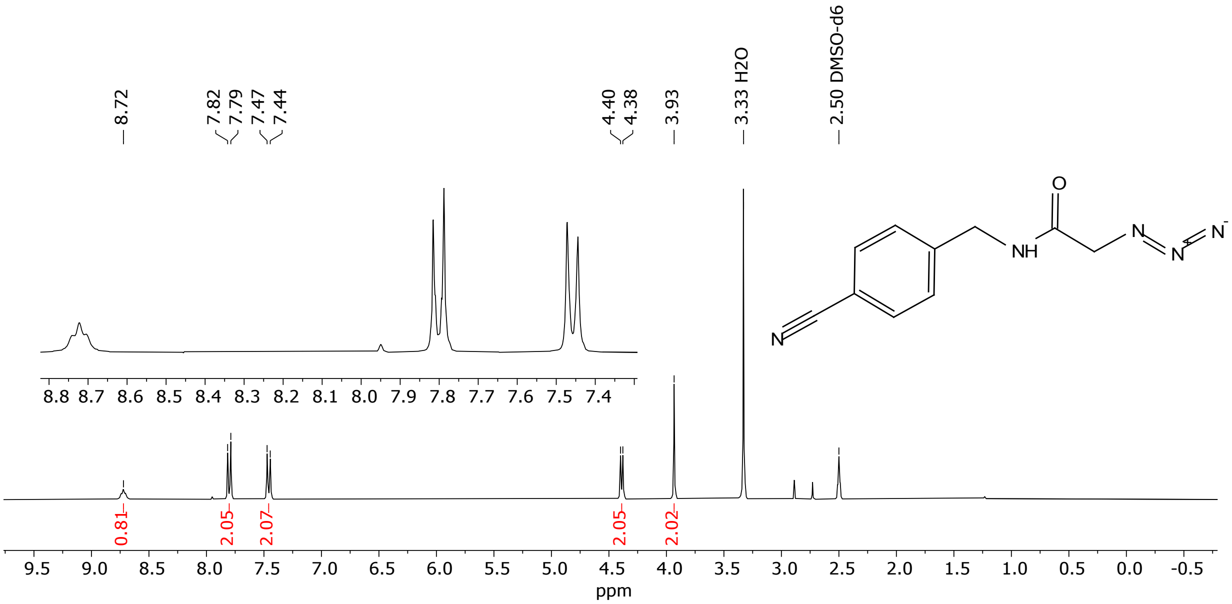

Fig S23. ^{1}H NMR of compound **13** (300 MHz, DMSO-d6)

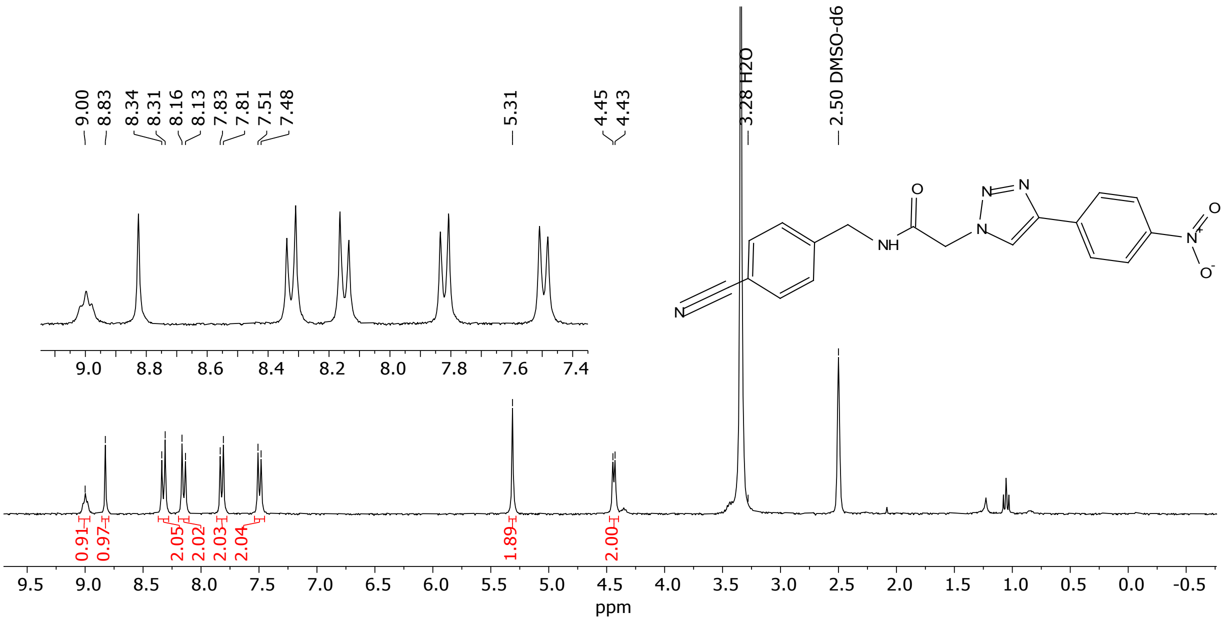

Fig S24. ^{13}C NMR of compound **13** (151 MHz, DMSO)

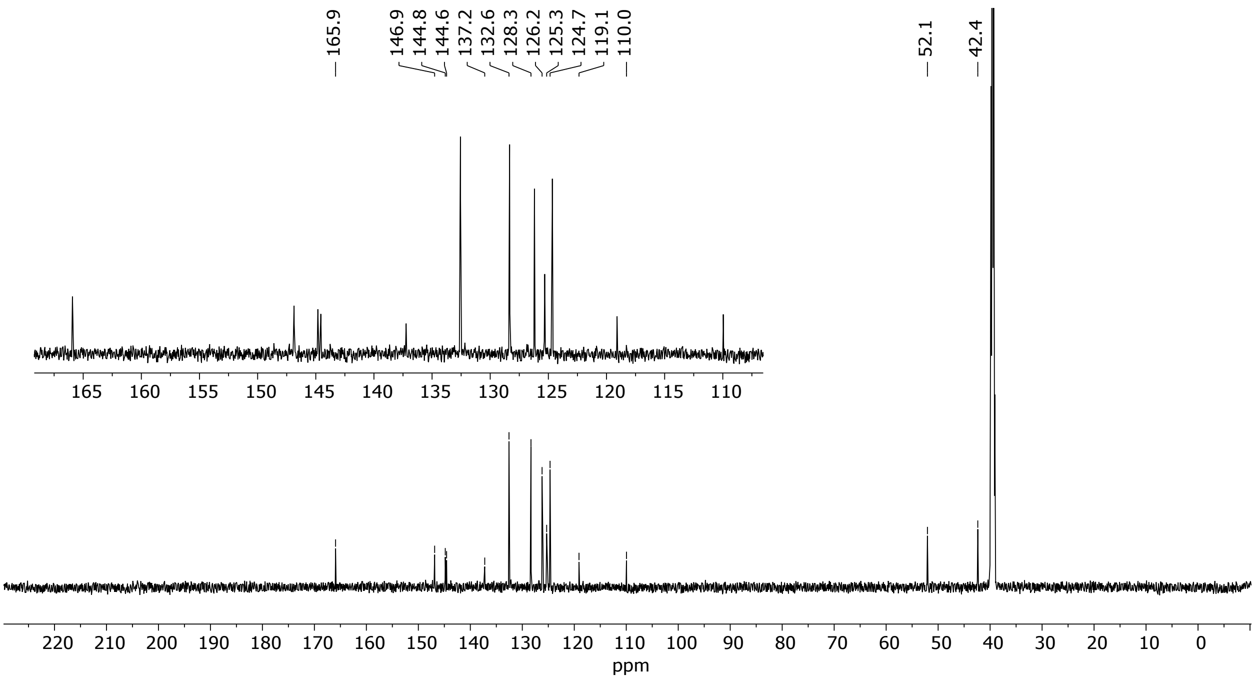

Fig S25. HRMS of compound **13**

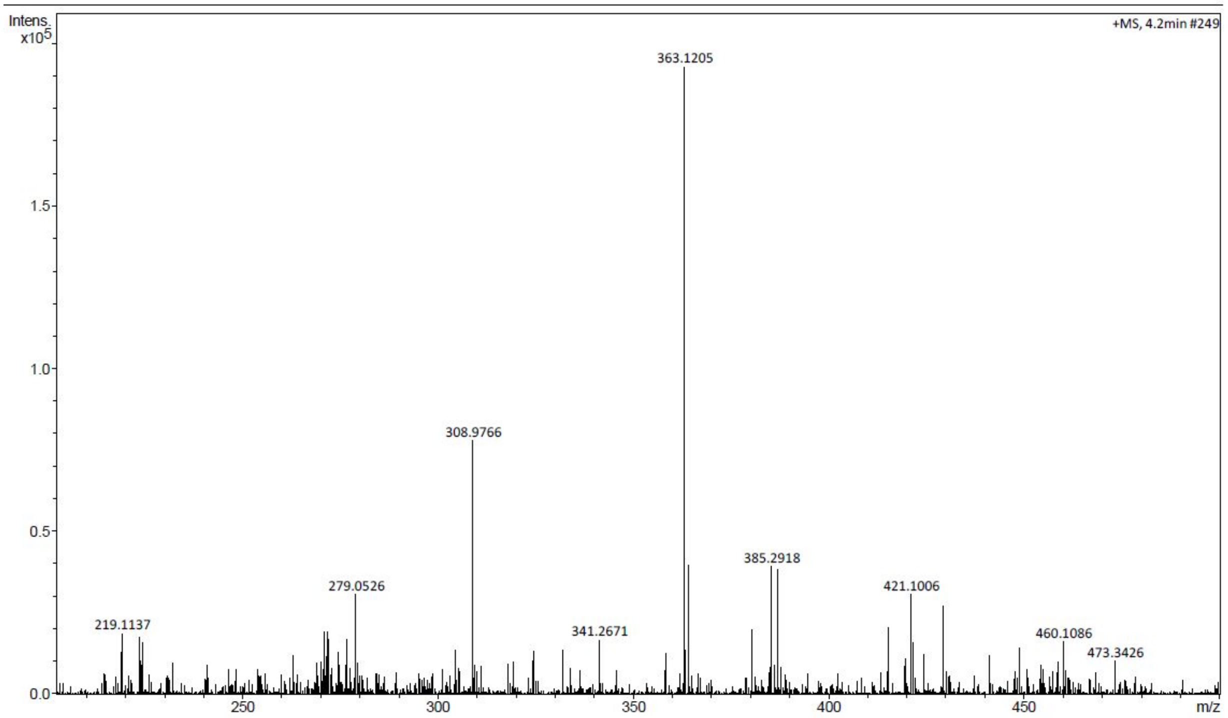

Fig S26. Chromatographic purity analysis of compound **13**

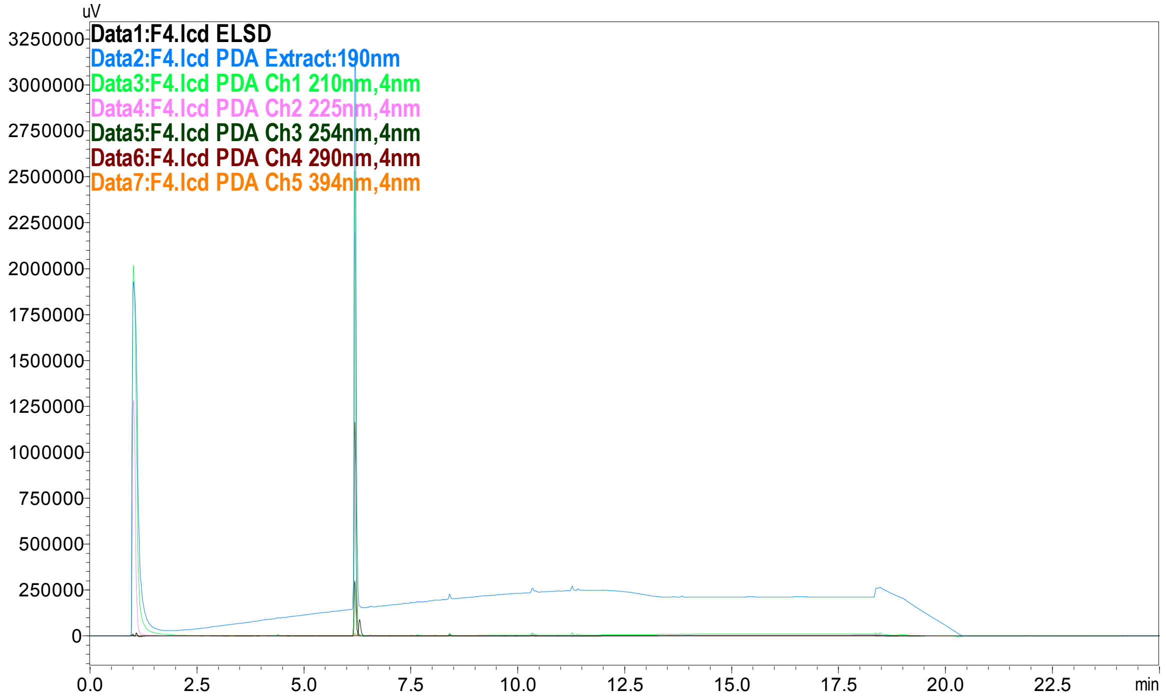

Characterization and purification of the second group of analogs

Fig S27. ^{1}H NMR of compound **15** (300 MHz, DMSO-d6)

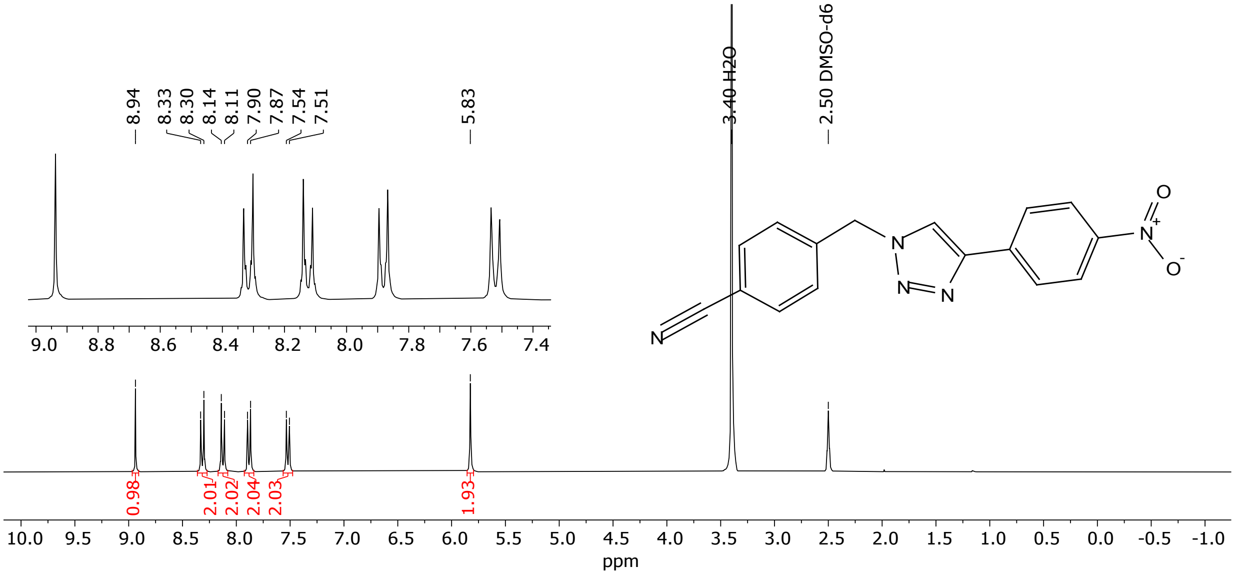

Fig S28. Infrared spectrum of compound **15**

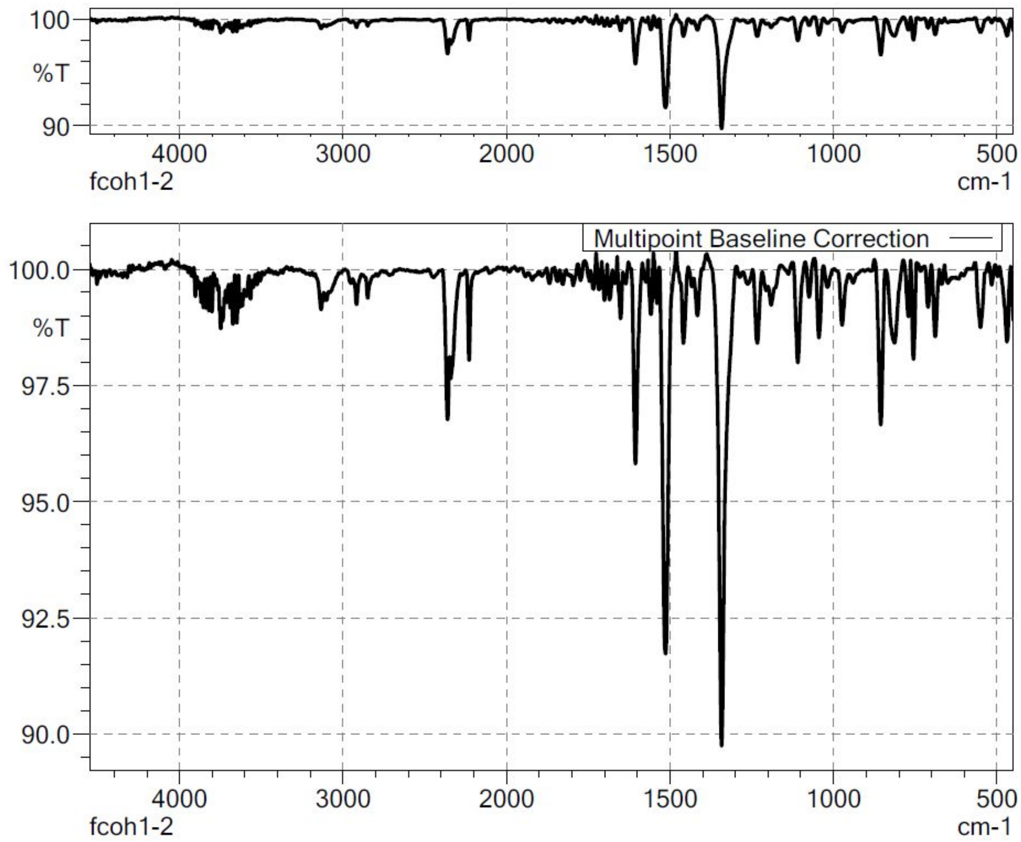

Fig S29. Chromatographic purity analysis of compound **15**

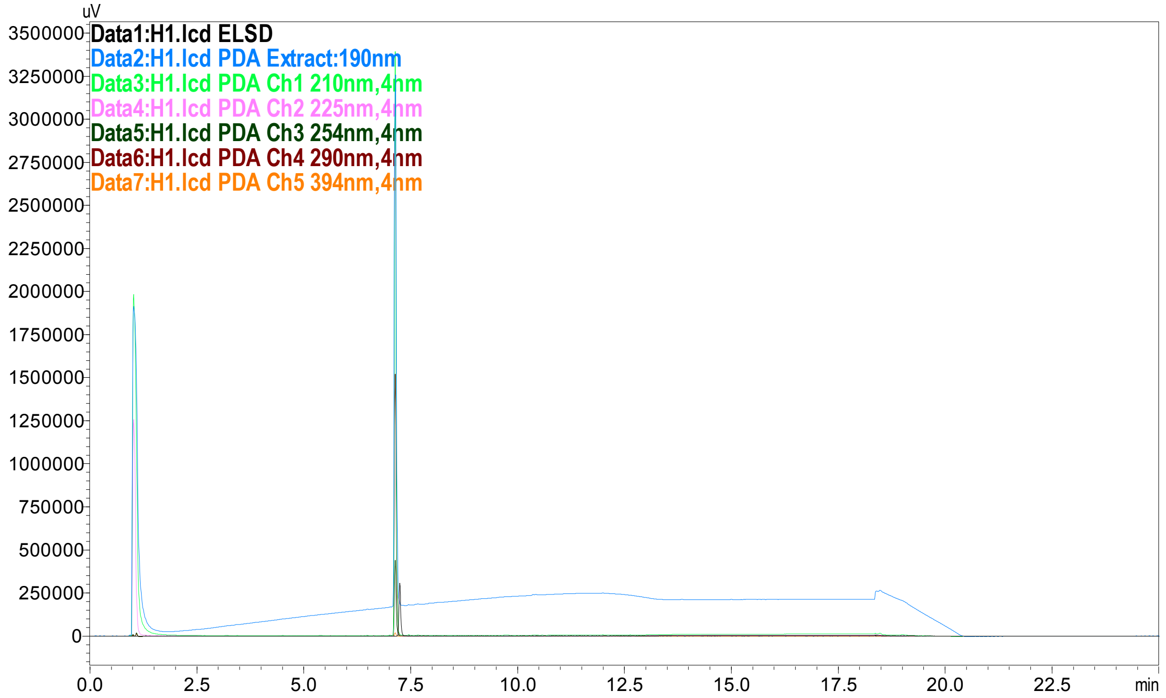

Fig S30. ^{1}H NMR of compound **18** (300 MHz, DMSO-d6)

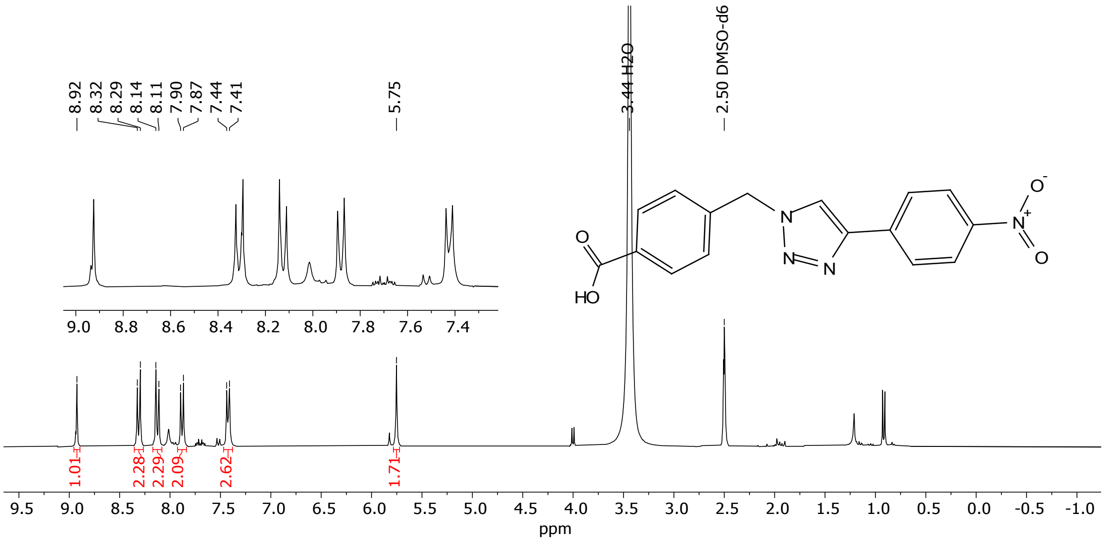

Fig S31. ^{13}C NMR of compound **18** (151 MHz, DMSO)

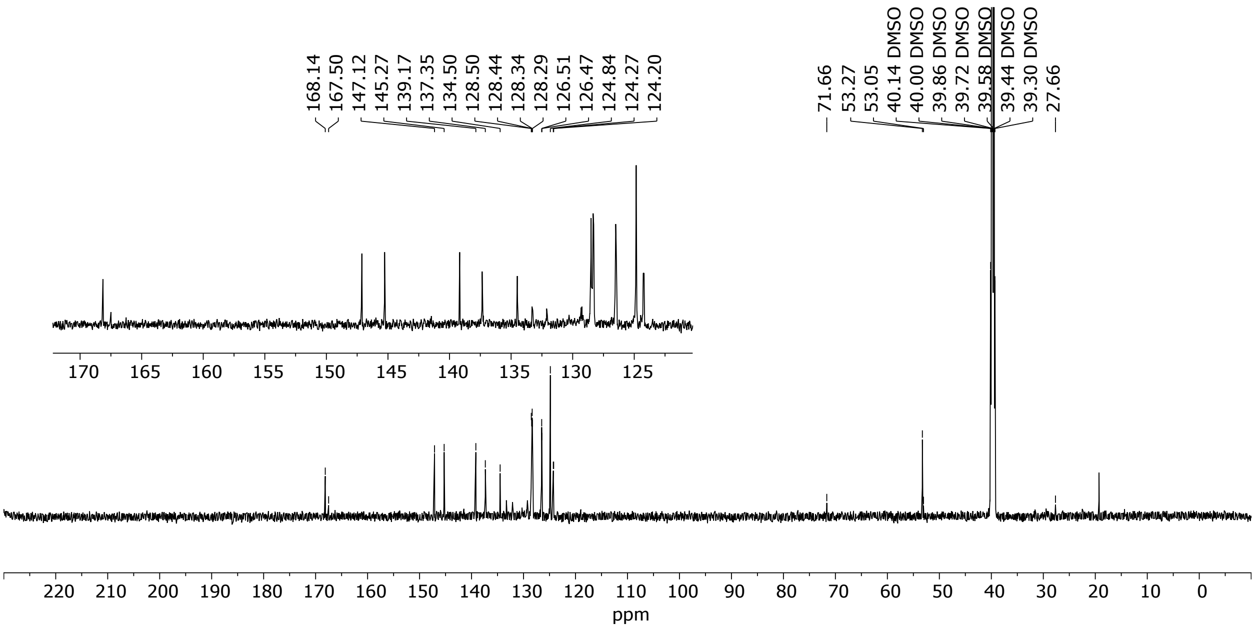

Fig S32. Infrared spectrum of compound **18**

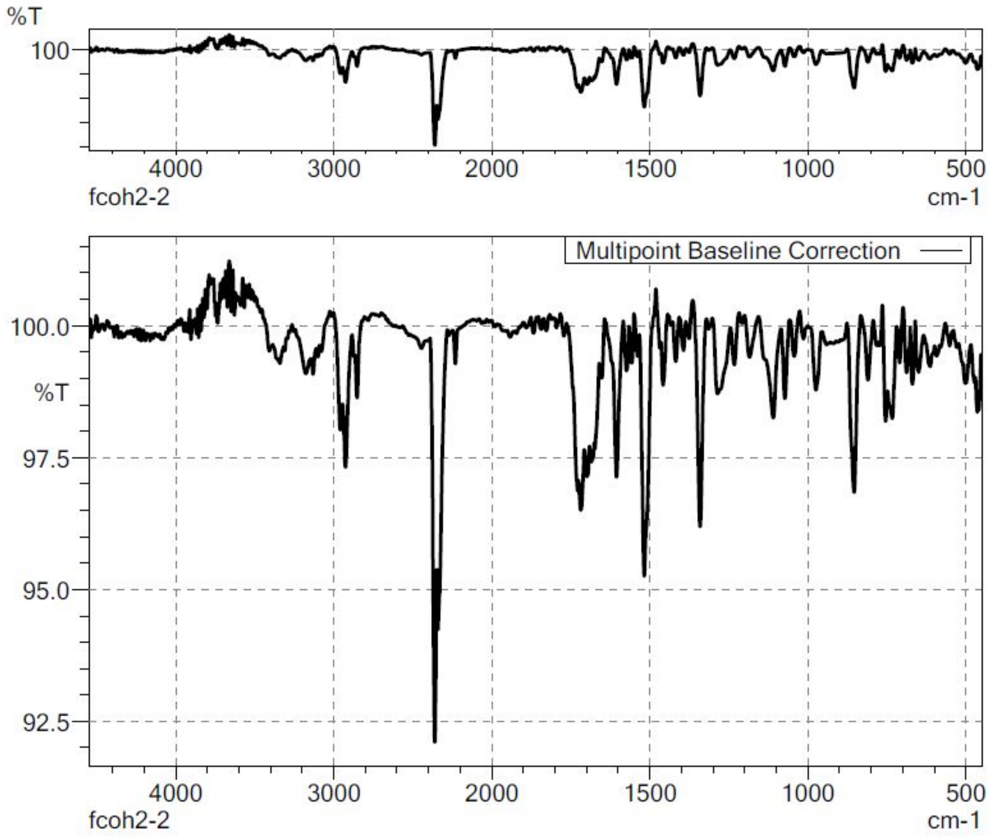

Fig S33. HRMS of compound **18**

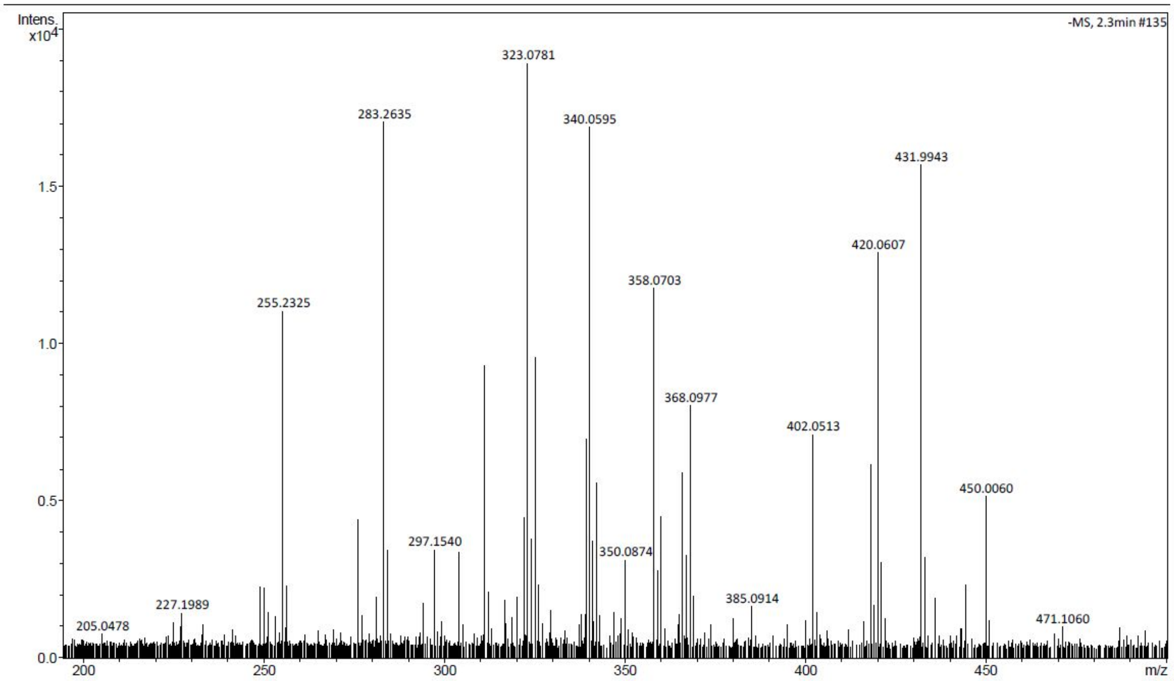

Fig S34. Chromatographic purity analysis of compound **18**

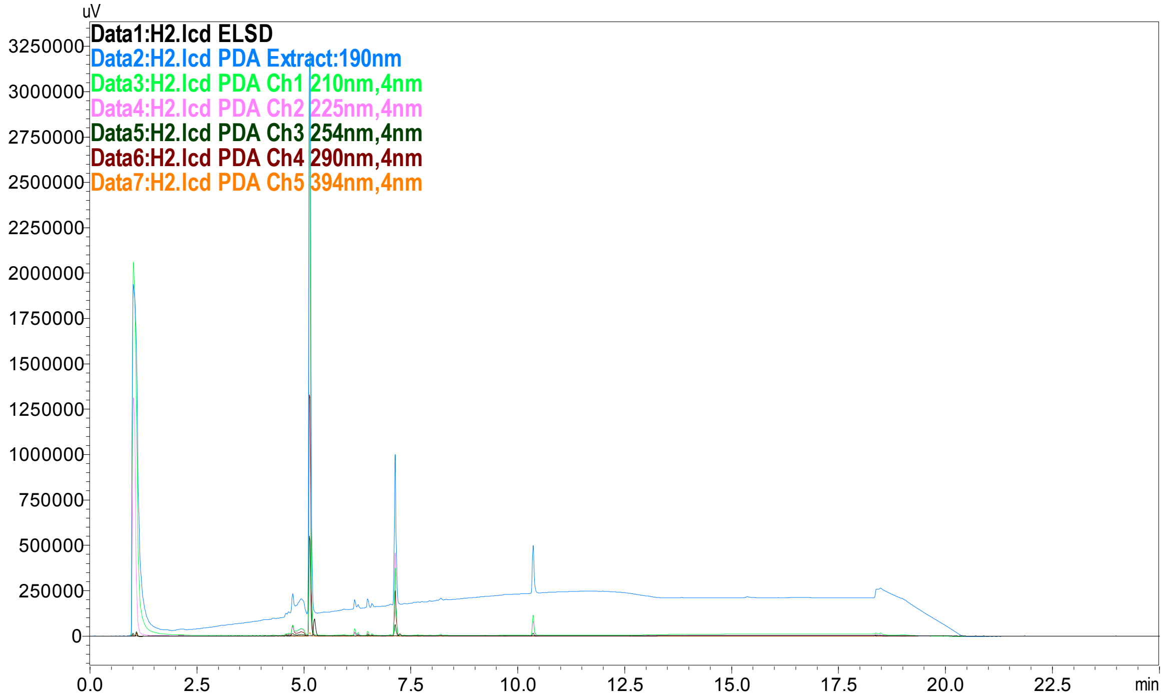

Fig S35. ^{1}H NMR of compound **16** (300 MHz, DMSO-d6)

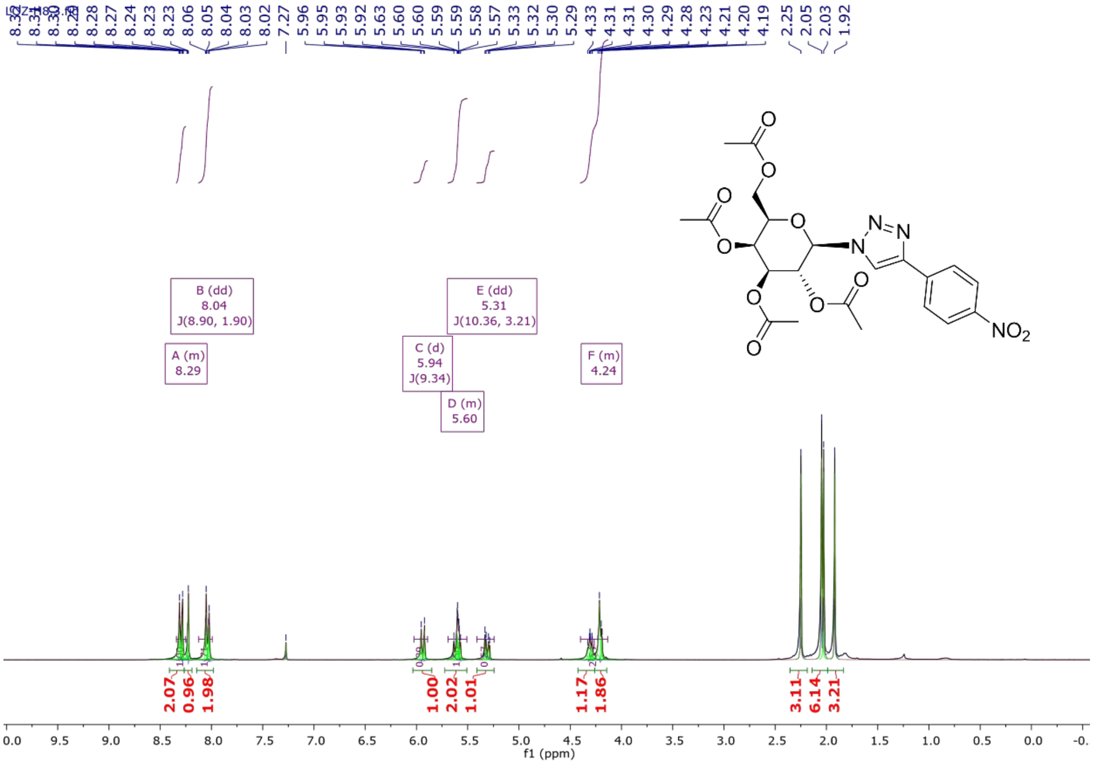

Fig S36. ^{13}C NMR of compound **16** (151 MHz, DMSO)

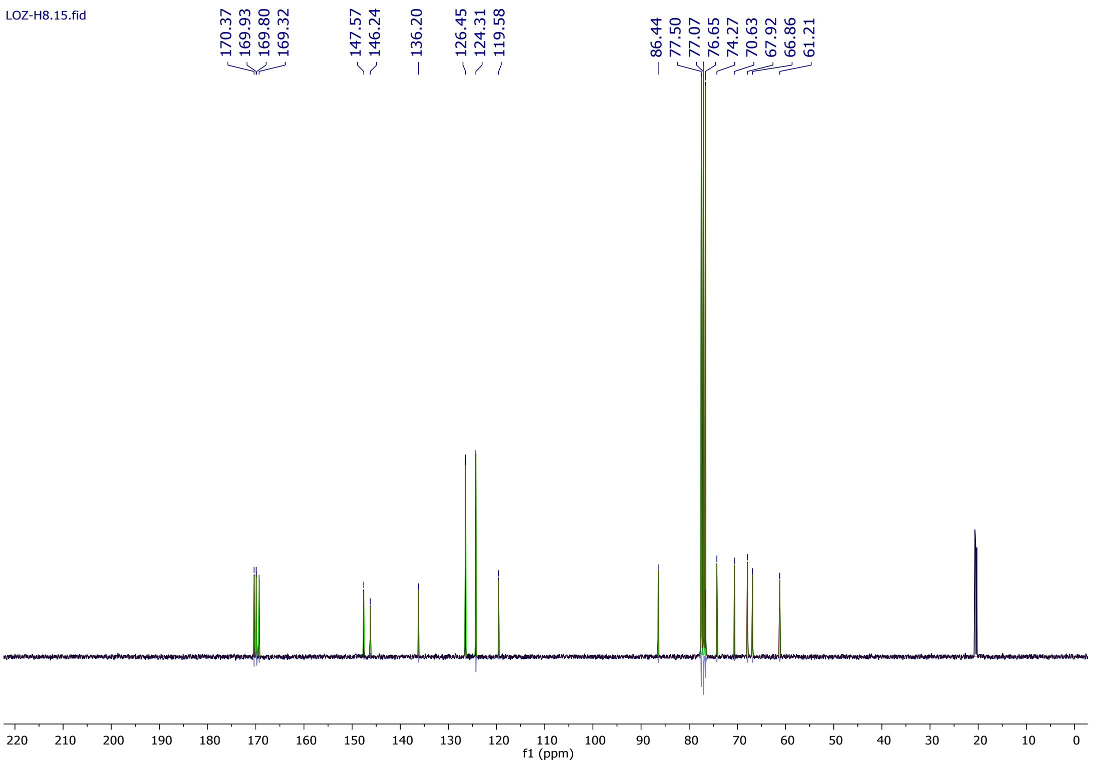

Fig S37. DEPT 135 of compound **16**

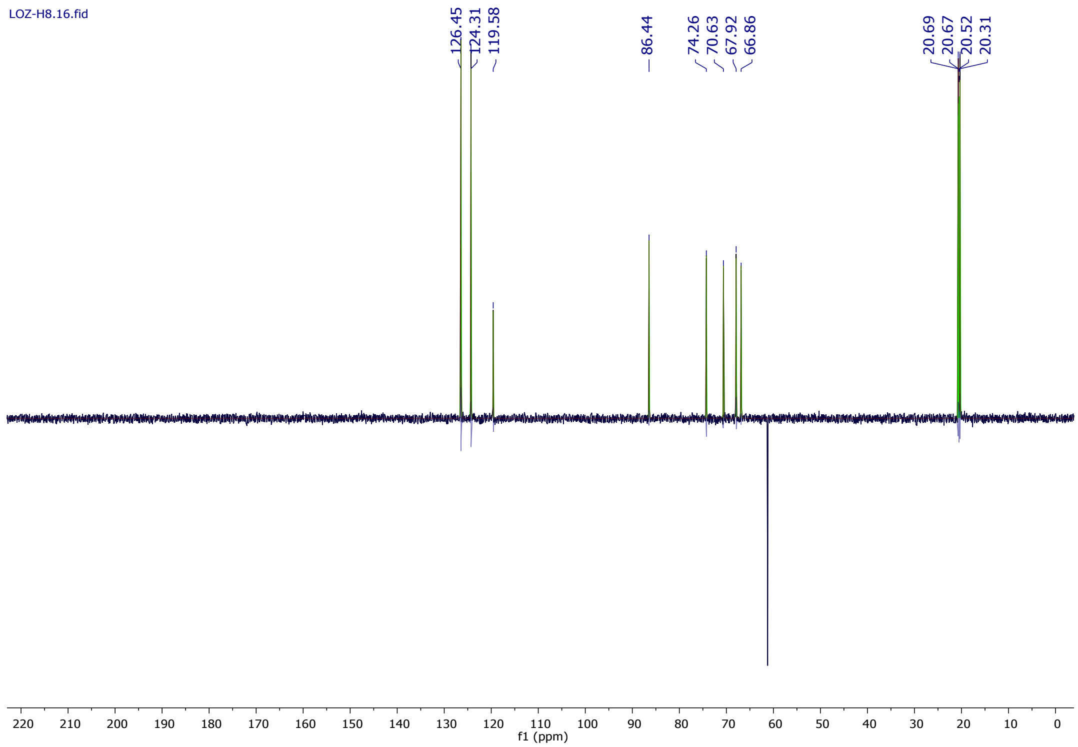

Fig S38. COSY of compound **16** ($CDCl_3$)

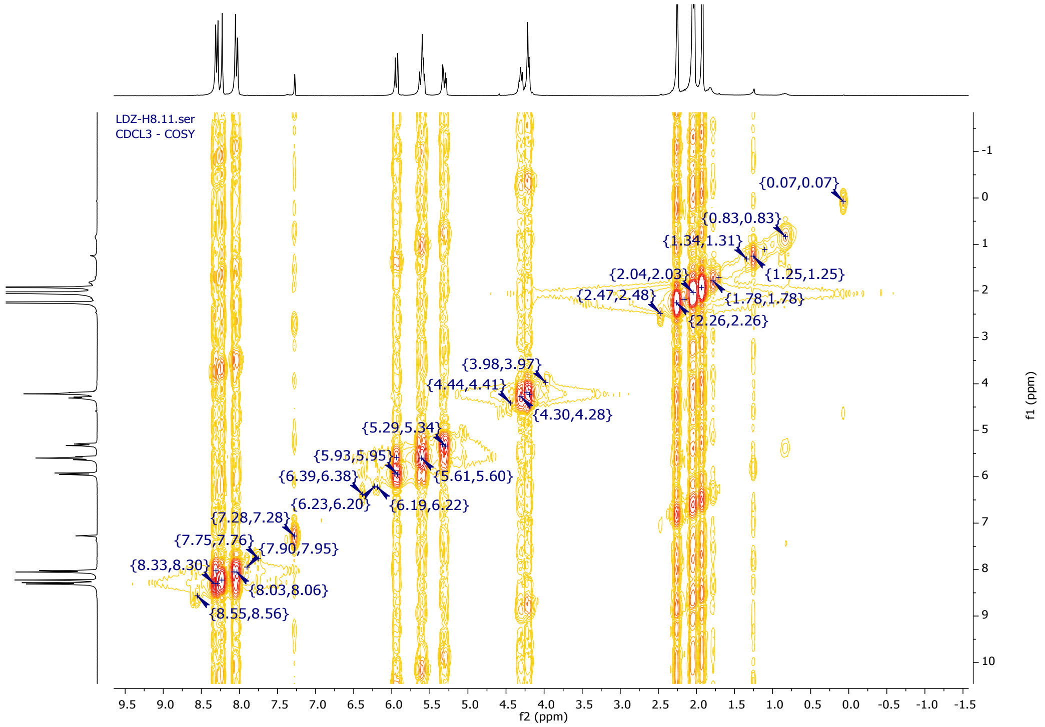

Fig S39. HSQC of compound **16** ($CDCl_3$)

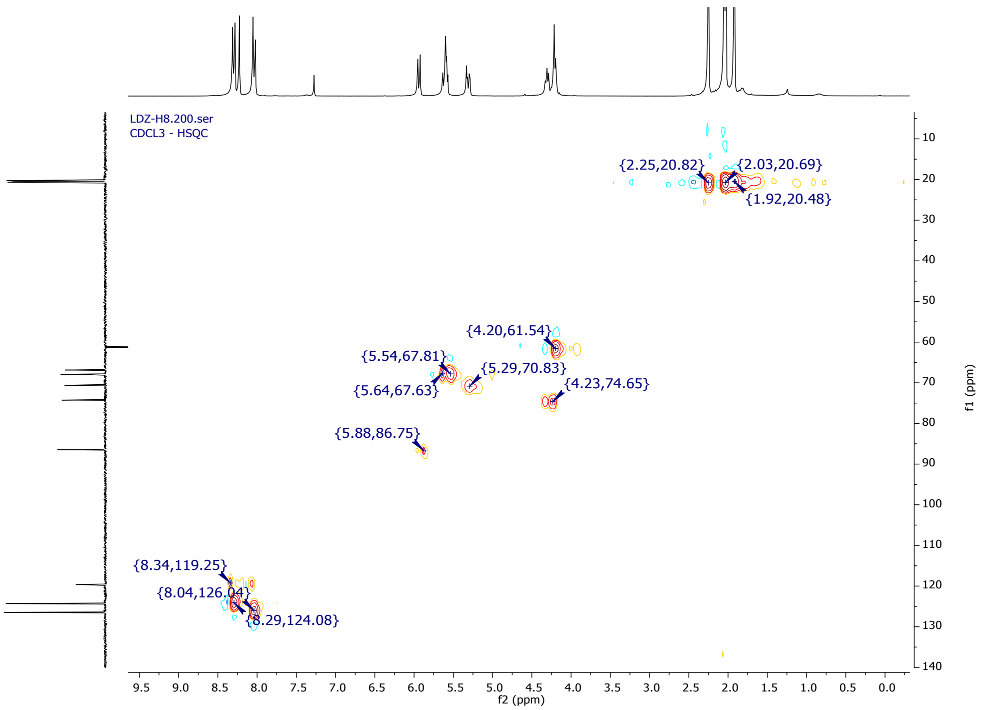

Fig S40. HRMS of compound **16**

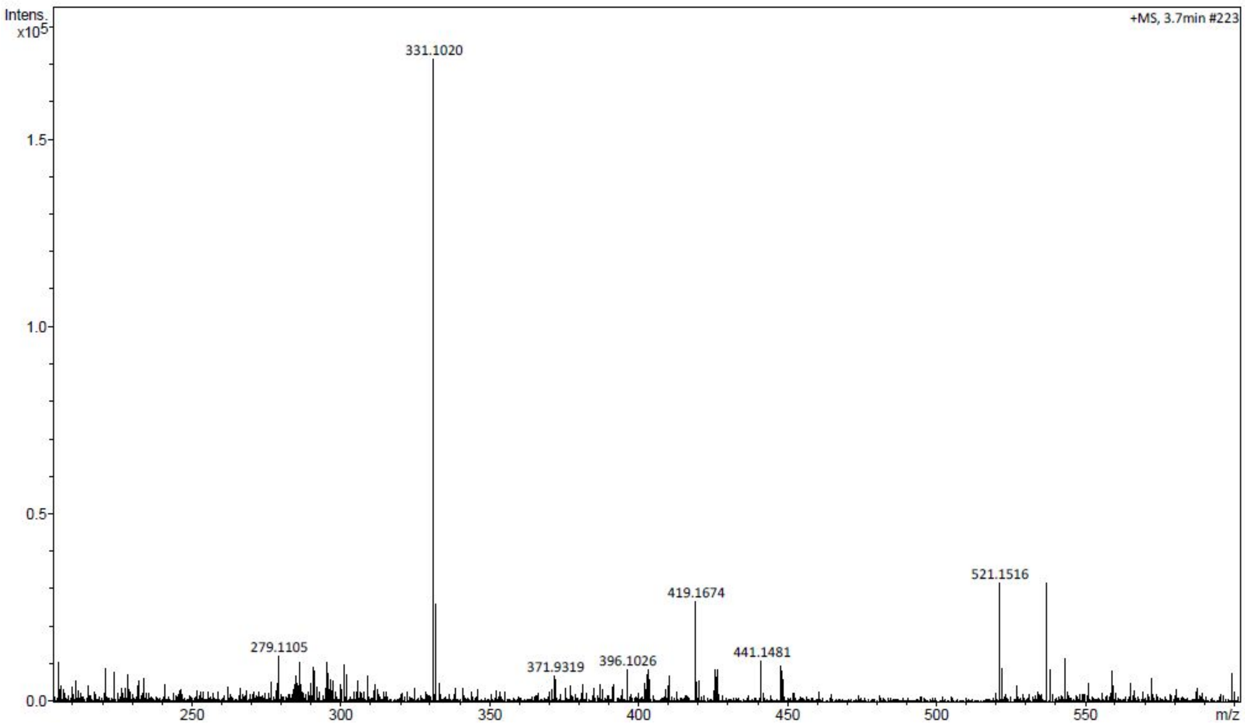

Fig S41. Chromatographic purity analysis of compound **16**

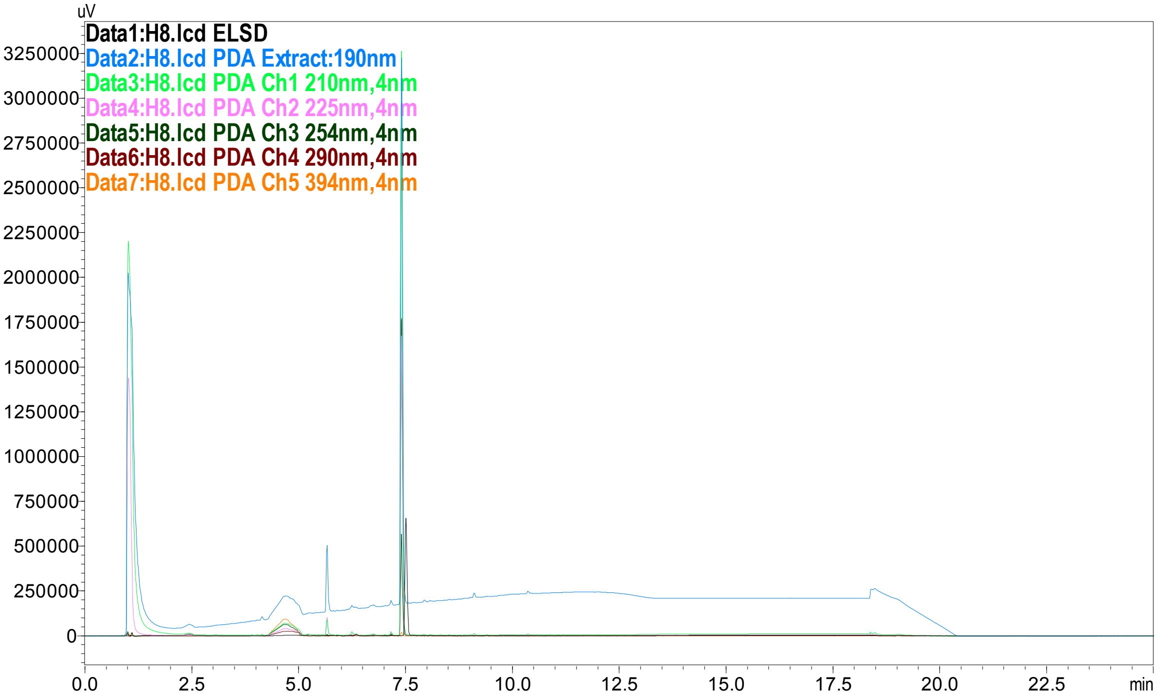

Fig S42. ^{1}H NMR of compound **14** (300 MHz, DMSO-d6)

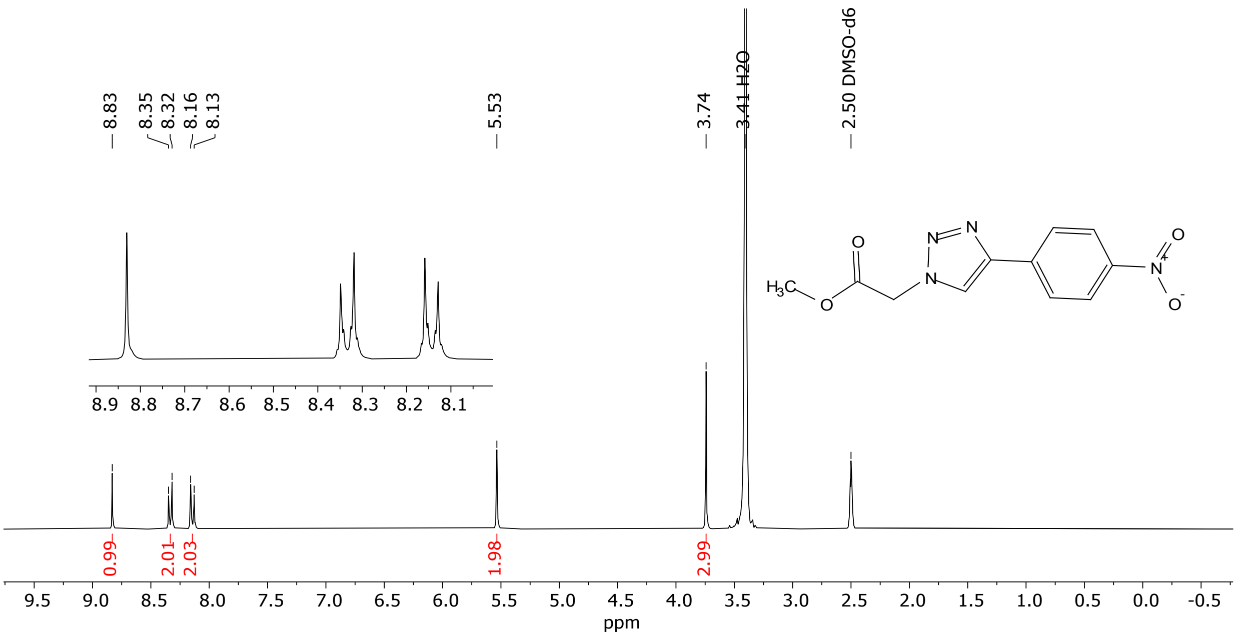

Fig S43. ^{1}H NMR of compound **17** (300 MHz, DMSO-d6)

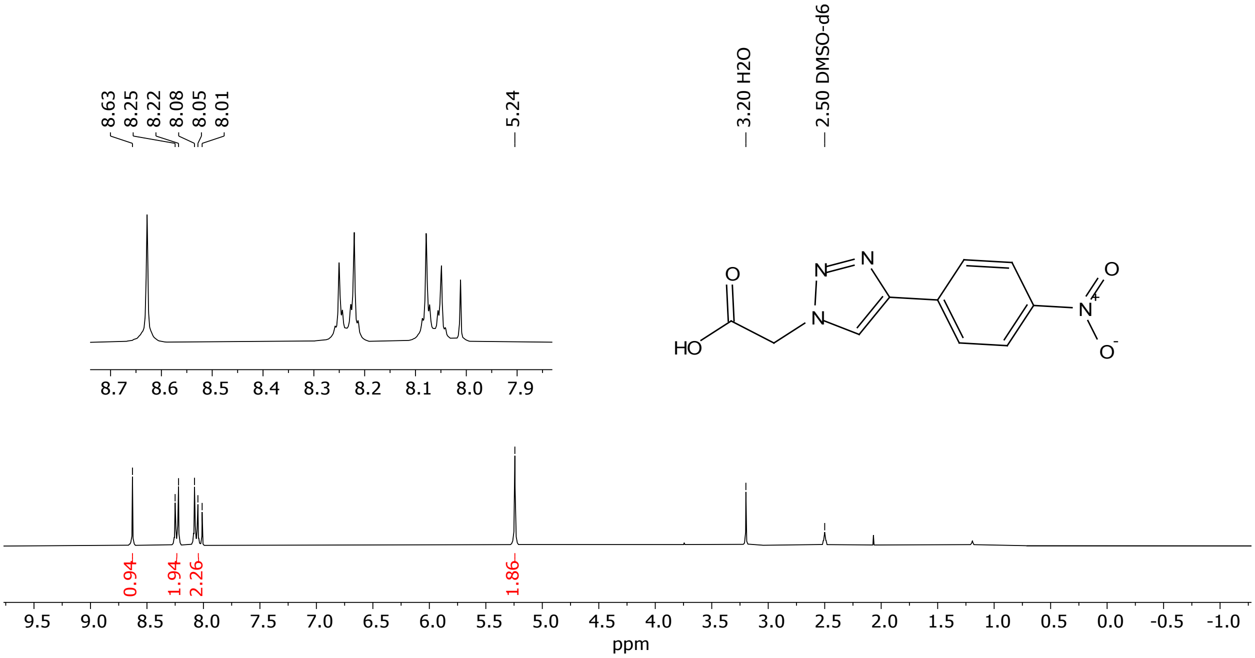

Fig S44. ^{13}C NMR of compound **17** (75 MHz, DMSO)

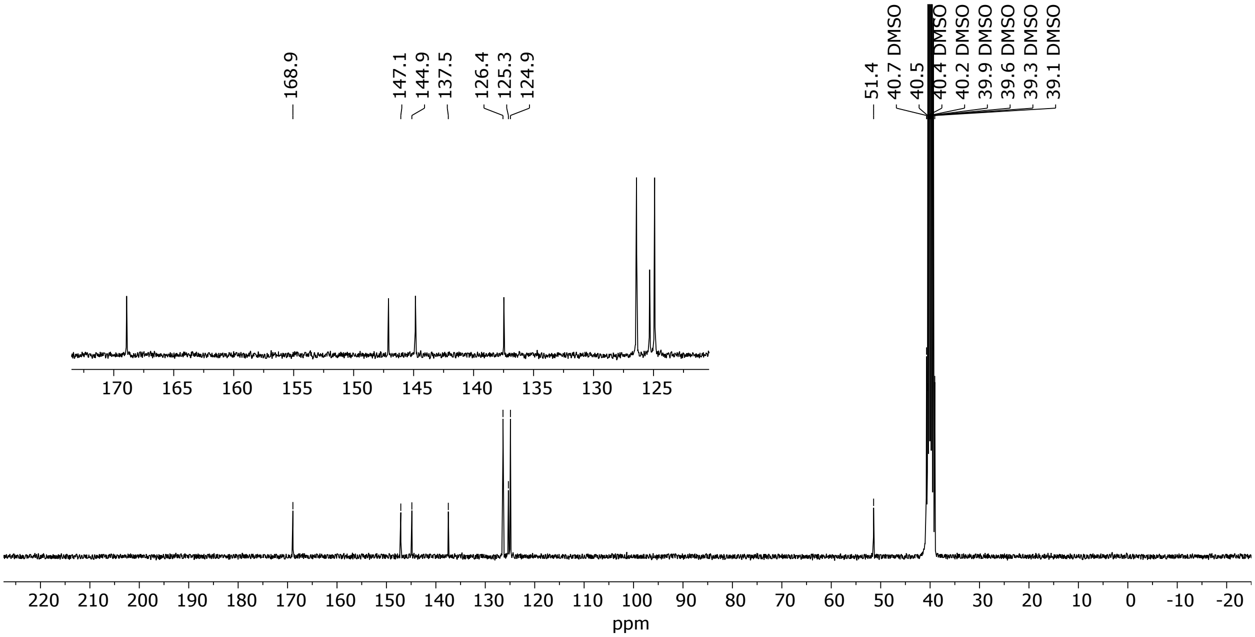

Fig S45. ^{1}H NMR of compound **19** (300 MHz, DMSO-d6)

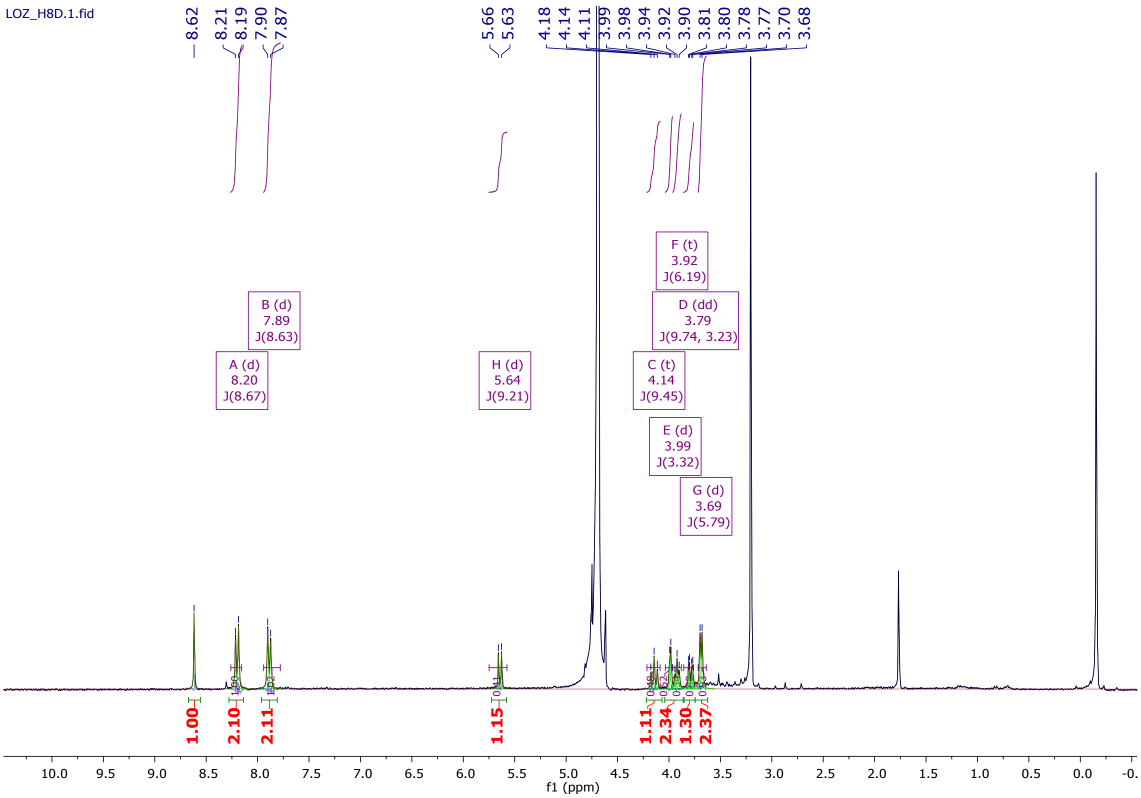

Fig S46. COSY of compound **19** ($CDCl_3$)

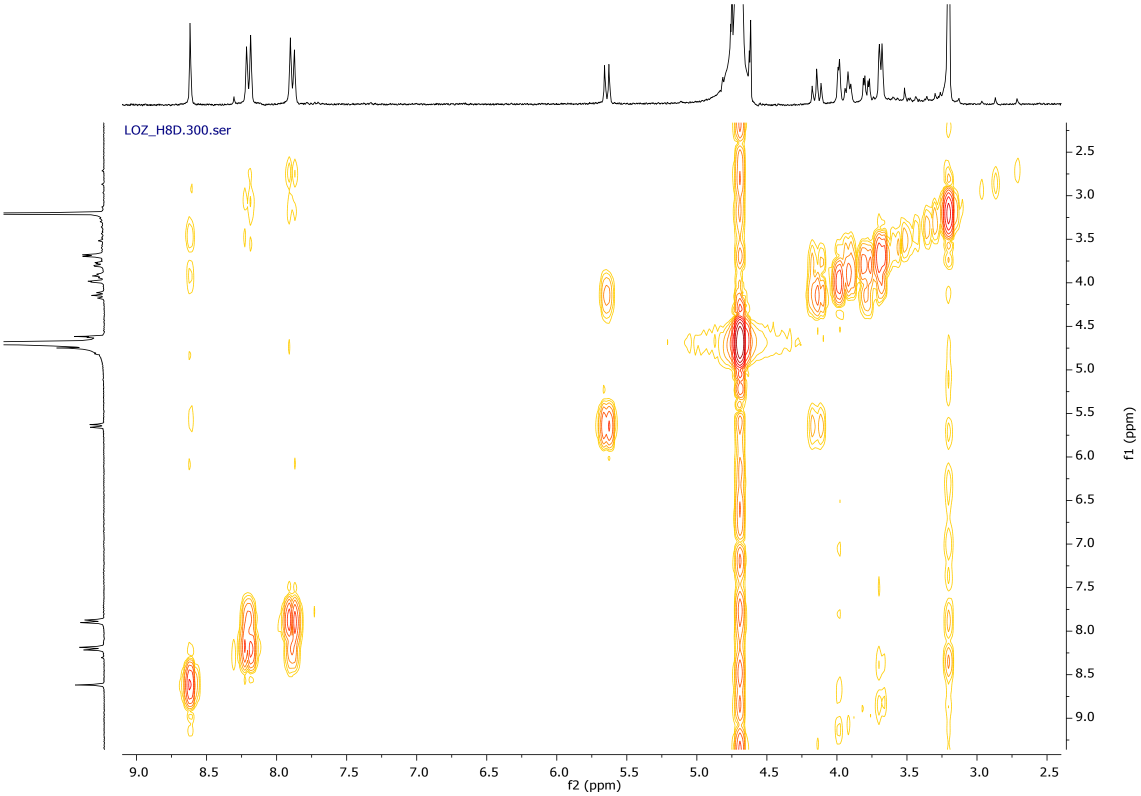

Fig S47. Chromatographic purity analysis of compound **19**

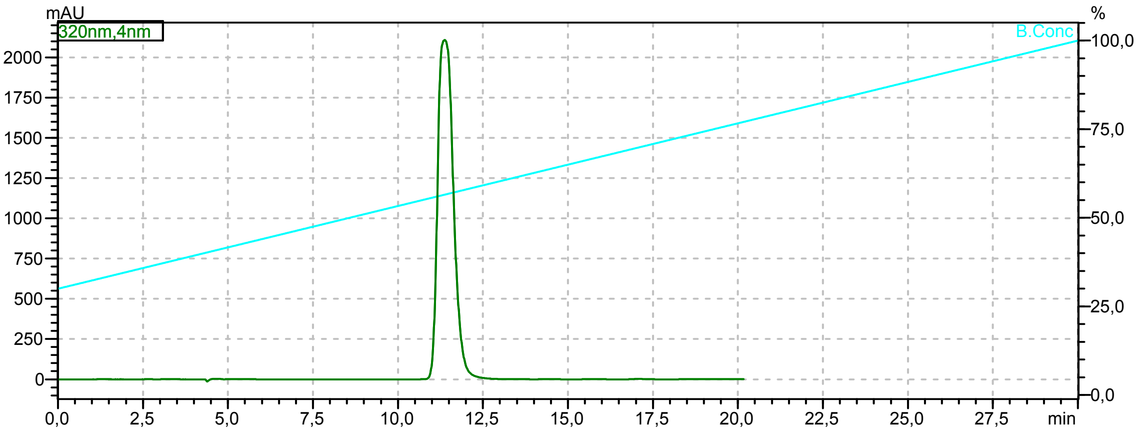

Characterization and purification of the third group of analogs

Fig S48. ^{1}H NMR of compound **25** (300 MHz, DMSO-d6)

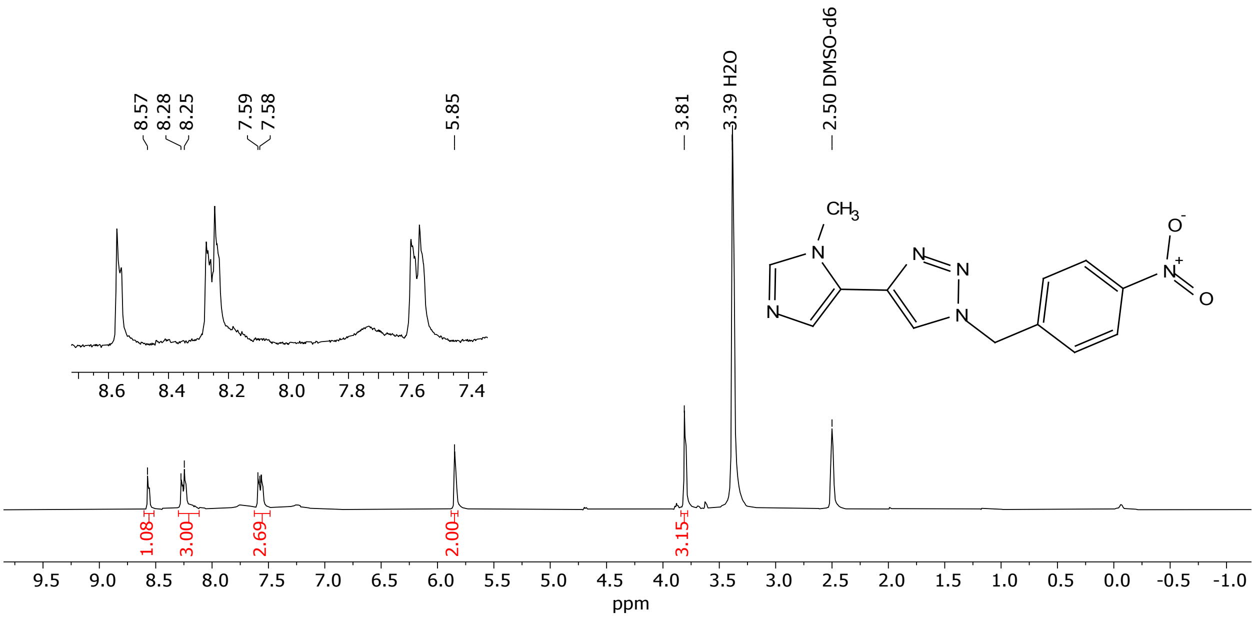

Fig S49. ^{13}C NMR of compound **25** (75 MHz, DMSO)

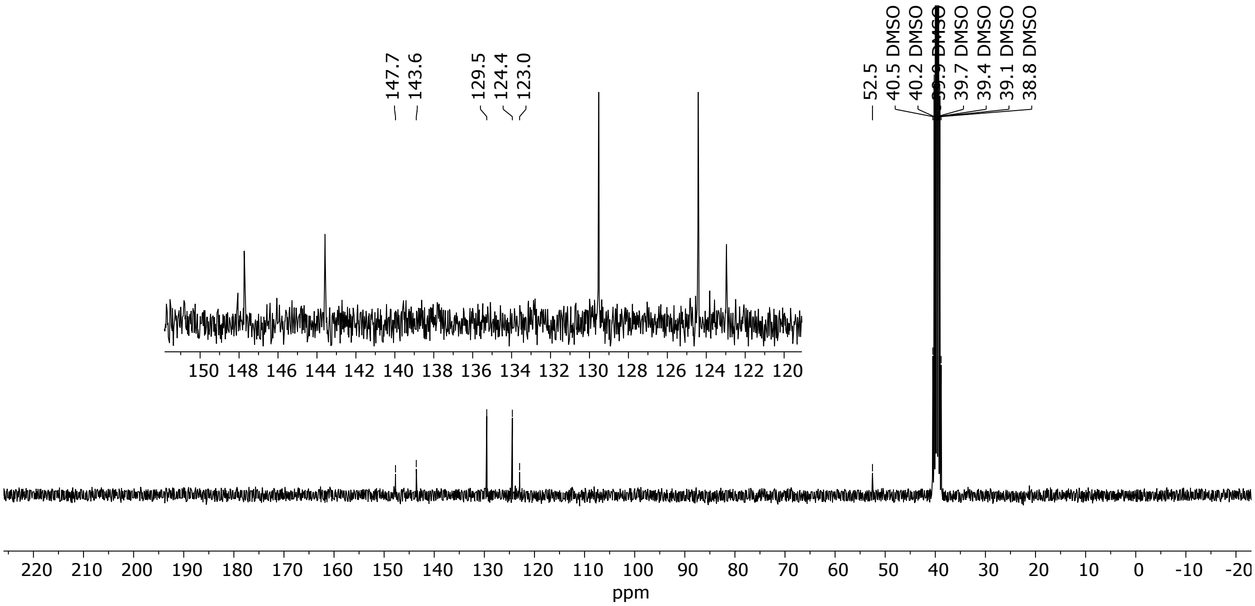

Fig S50. HRMS of compound **25**

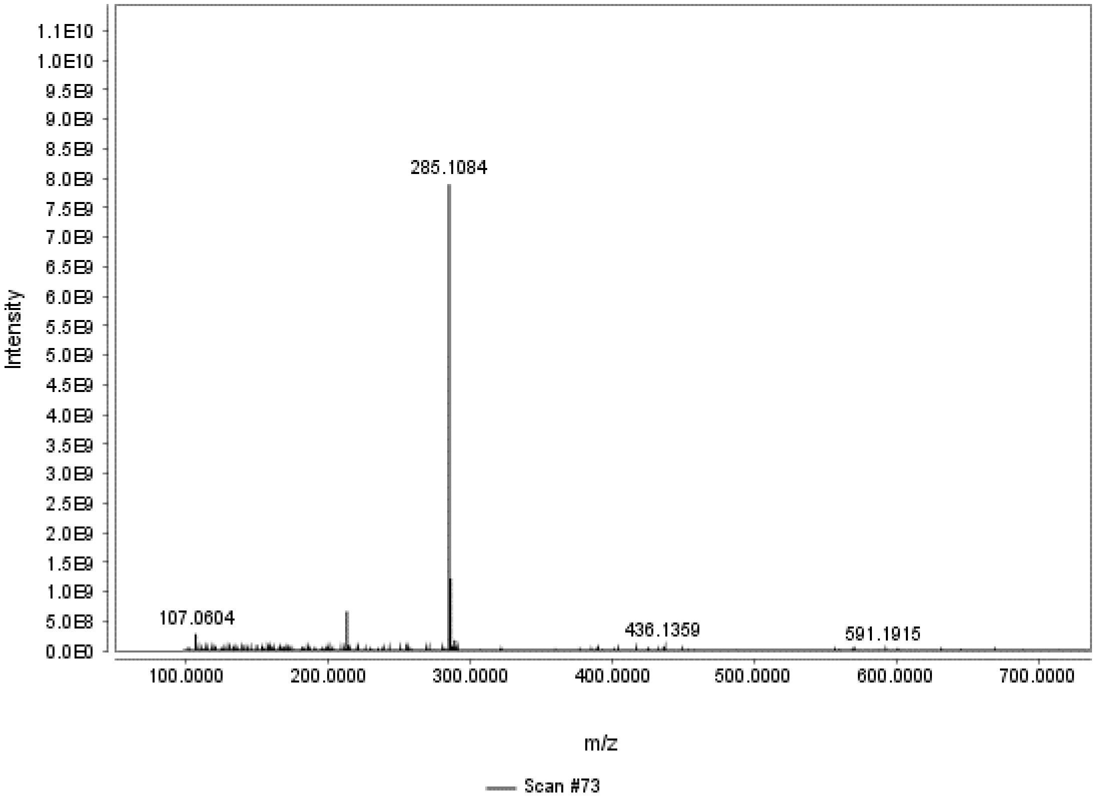

Fig S51. Chromatographic purity analysis of compound **25**

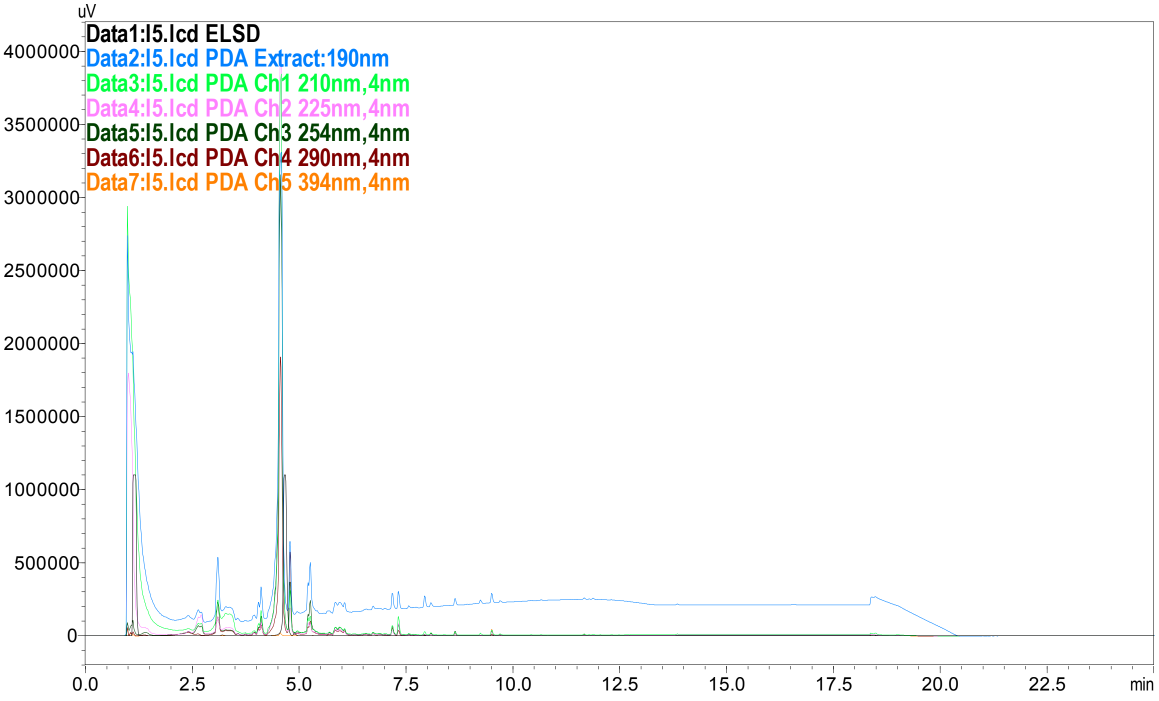

Fig S52. ^{1}H NMR of compound **22** (300 MHz, DMSO-d6)

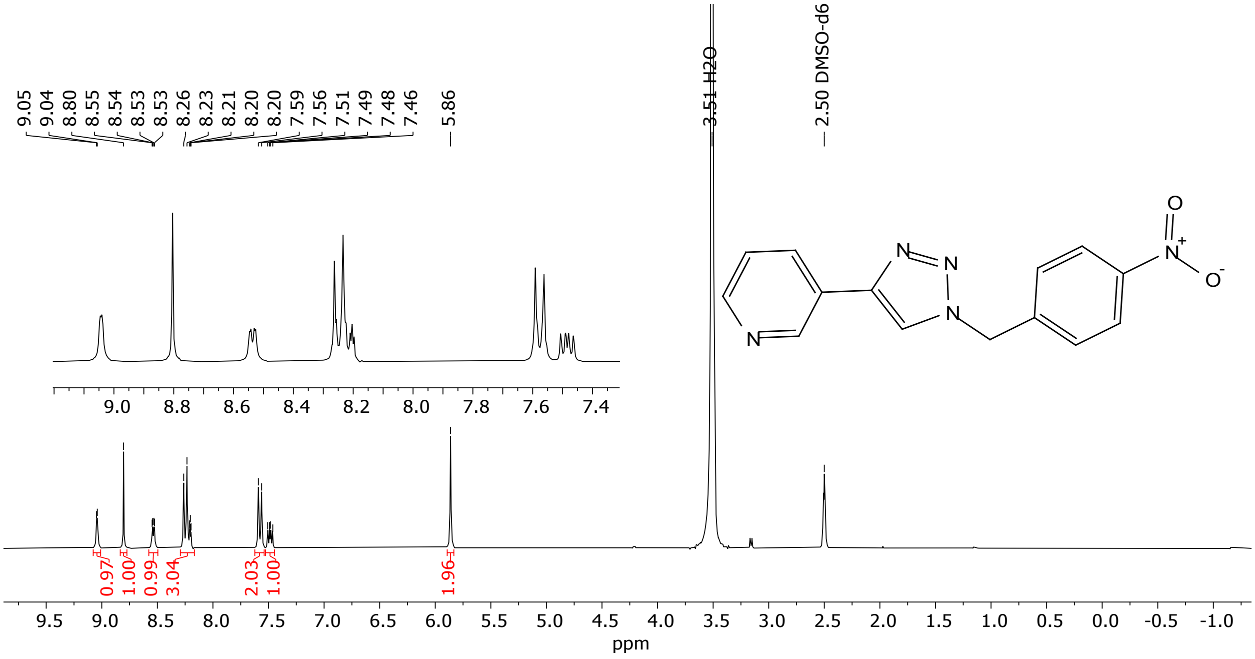

Fig S53. ^{13}C NMR of compound **22** (75 MHz, DMSO)

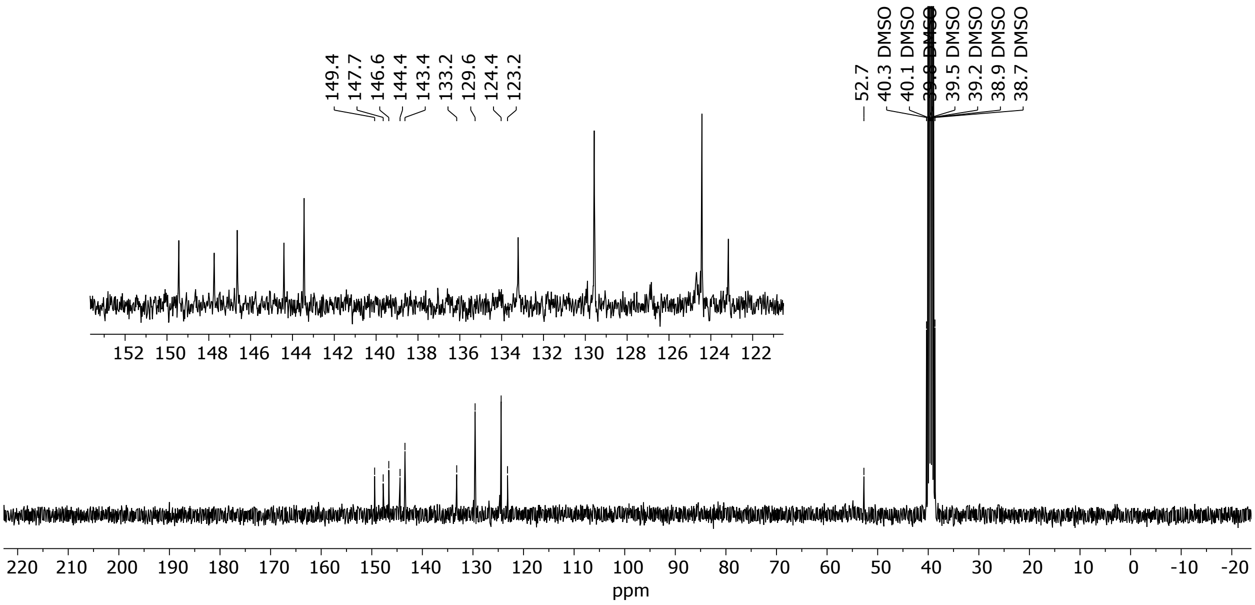

Fig S54. HRMS of compound **22**

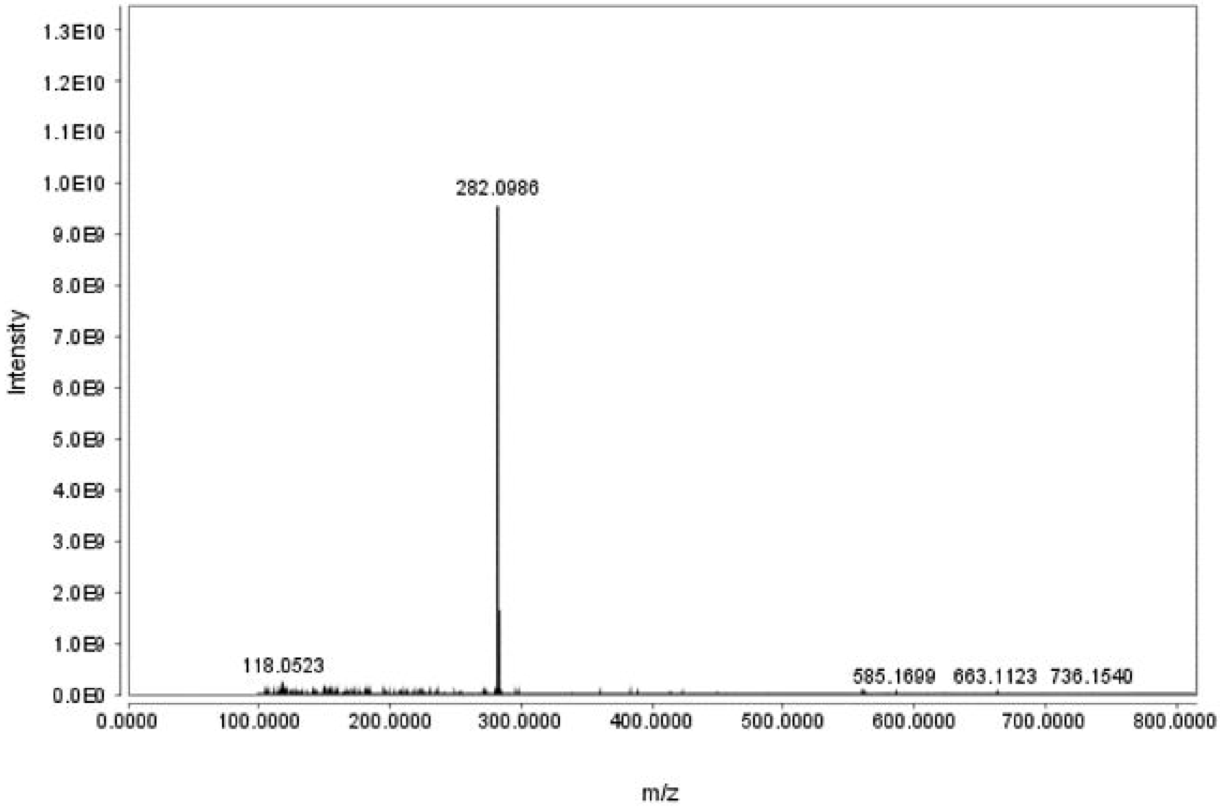

Fig S55. Chromatographic purity analysis of compound **22**

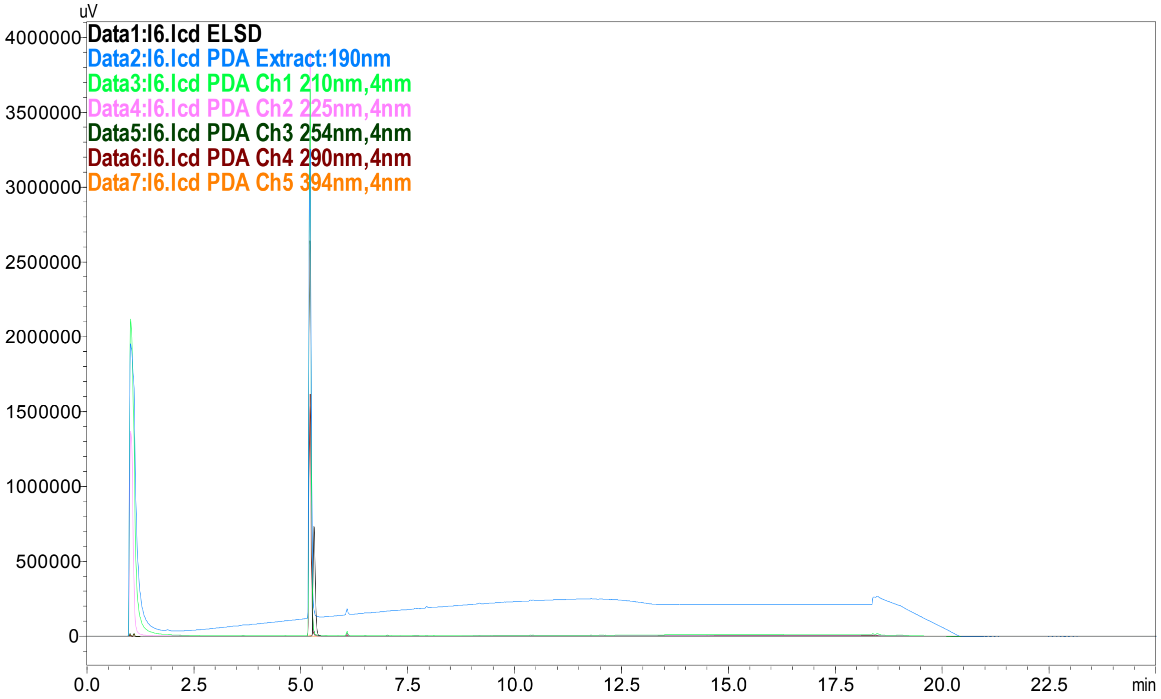

Fig S56. ^{1}H NMR of compound **21** (300 MHz, DMSO-d6)

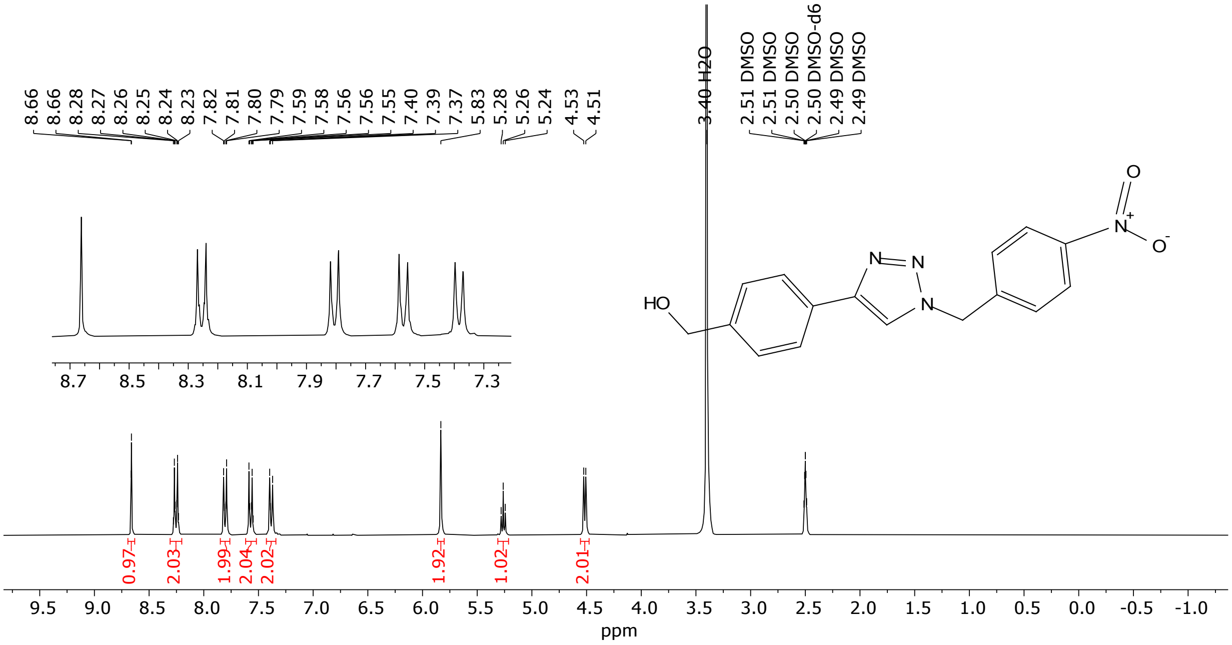

Fig S57. Chromatographic purity analysis of compound **21**

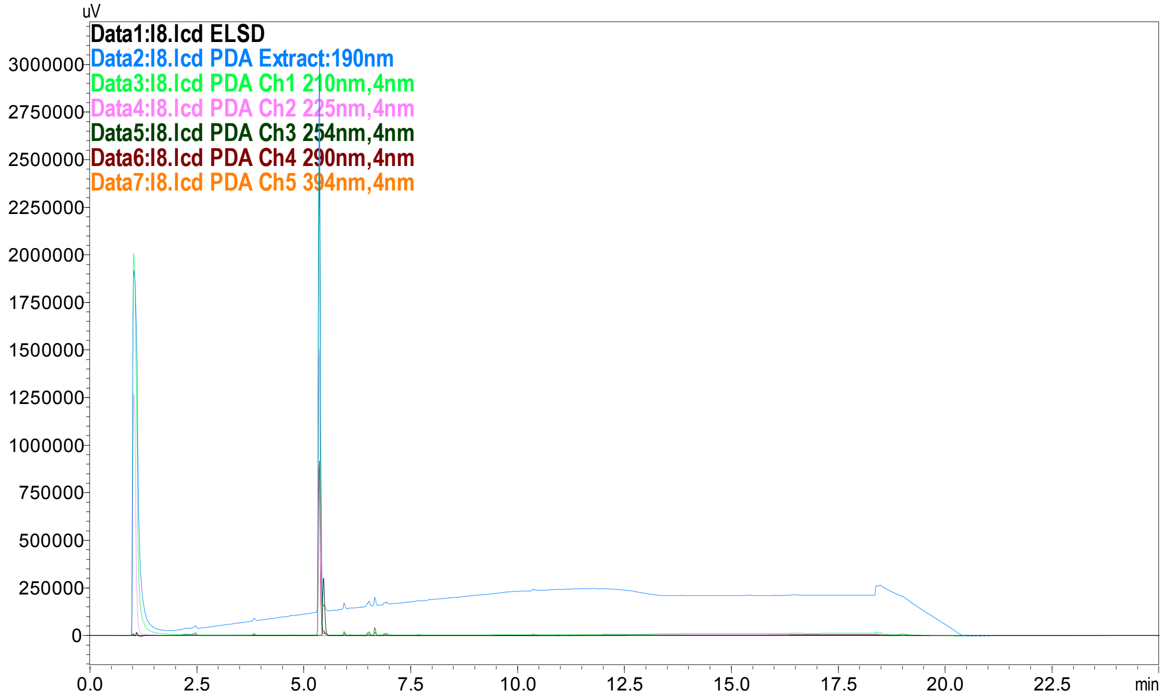

Fig S58. ^{1}H NMR of compound **27** (300 MHz, DMSO-d6)

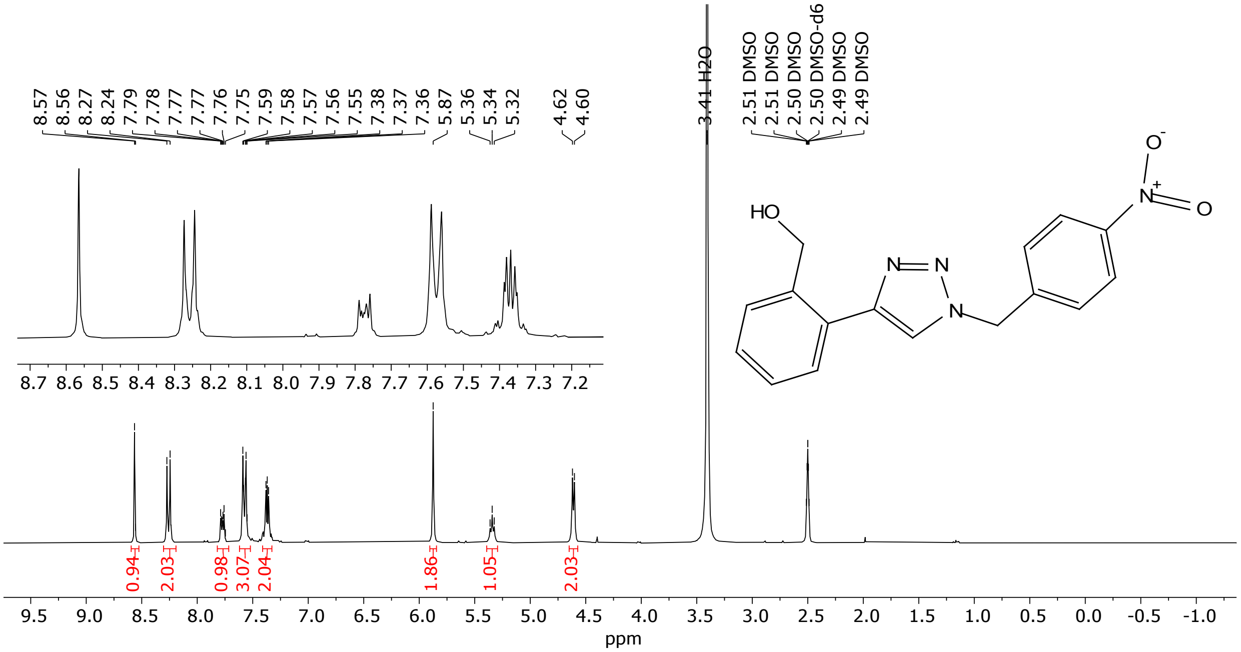

Fig S59. ^{13}C NMR of compound **27** (75 MHz, DMSO)

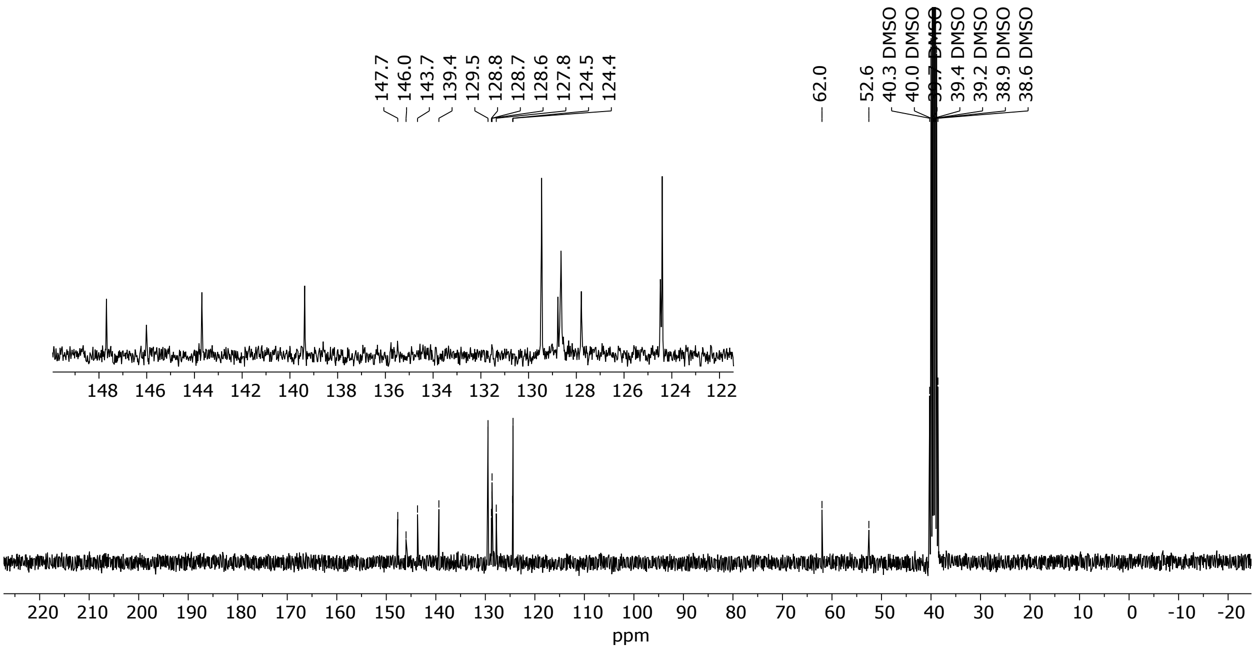

Fig S60. HRMS of compound **27**

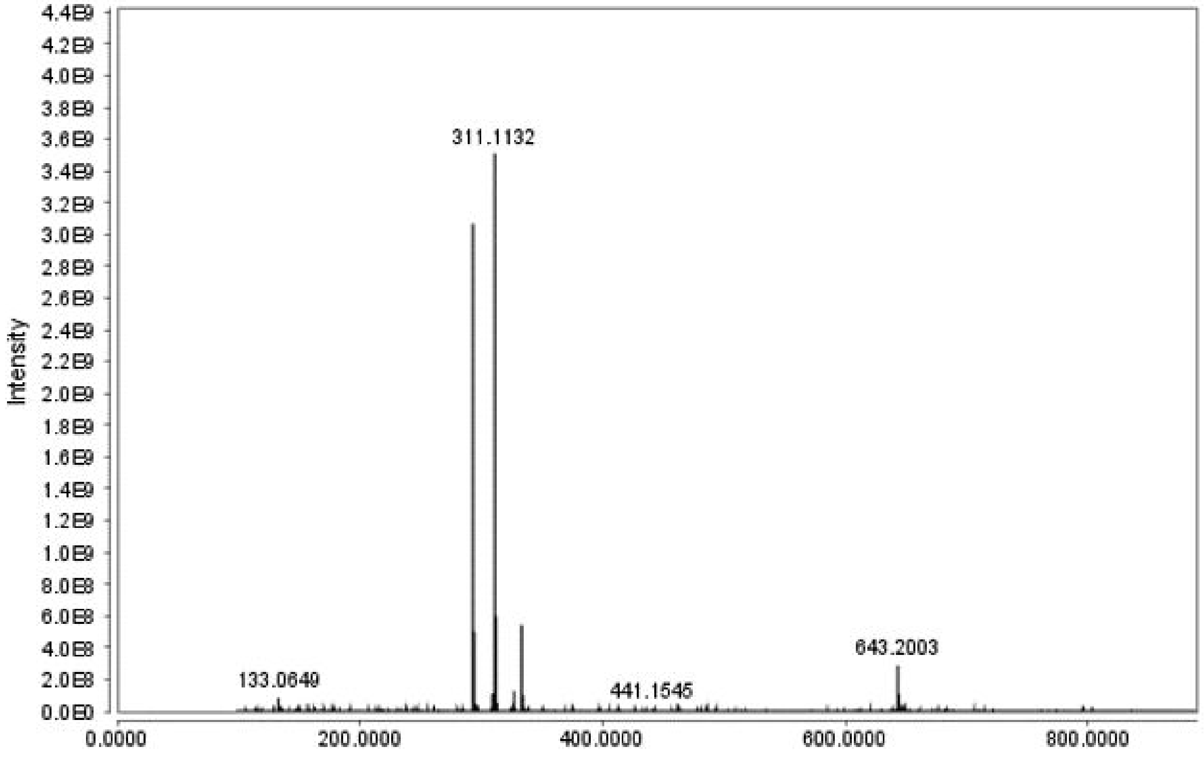

Fig S61. Chromatographic purity analysis of compound **27**

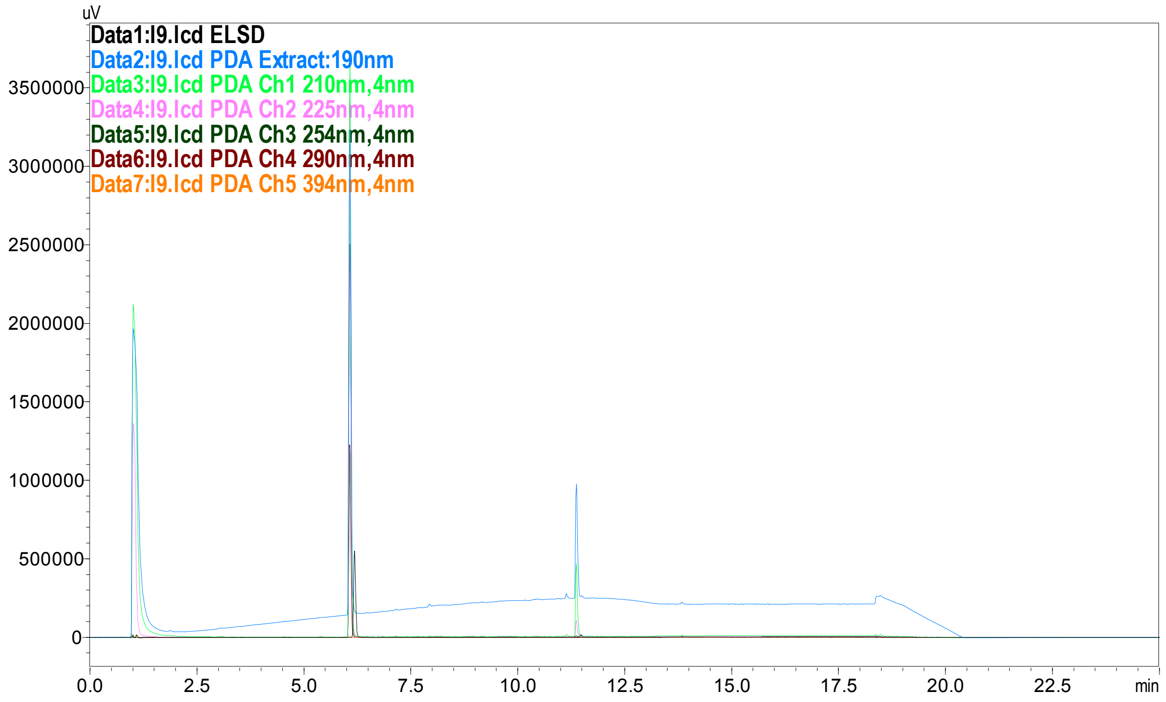

Fig S62. ^{1}H NMR of compound **26** (300 MHz, DMSO-d6)

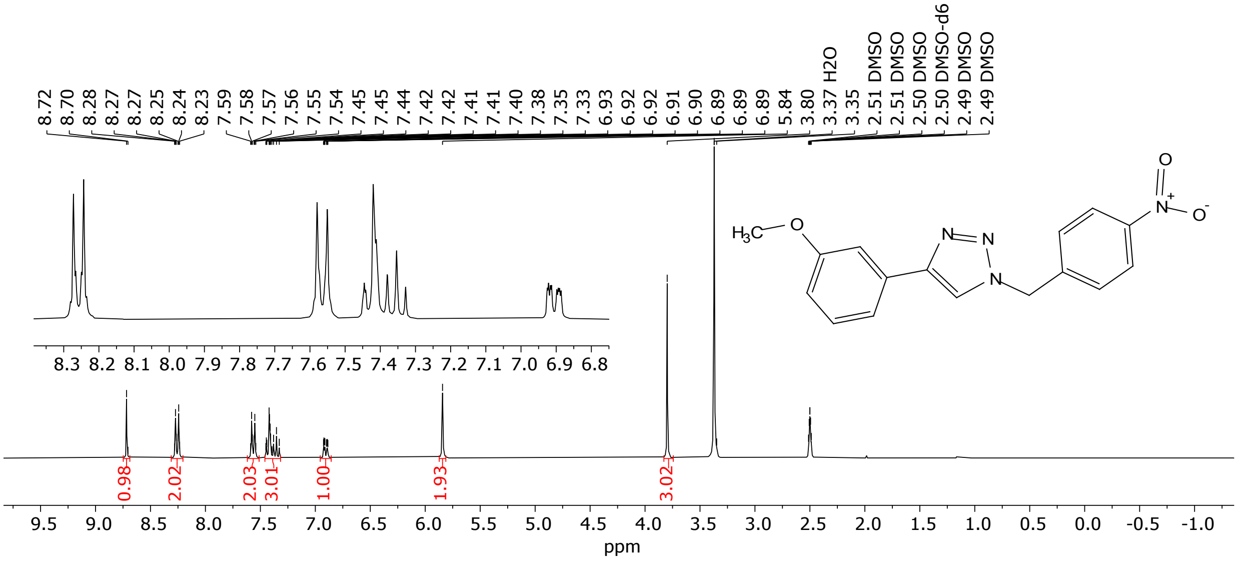

Fig S63. Chromatographic purity analysis of compound **26**

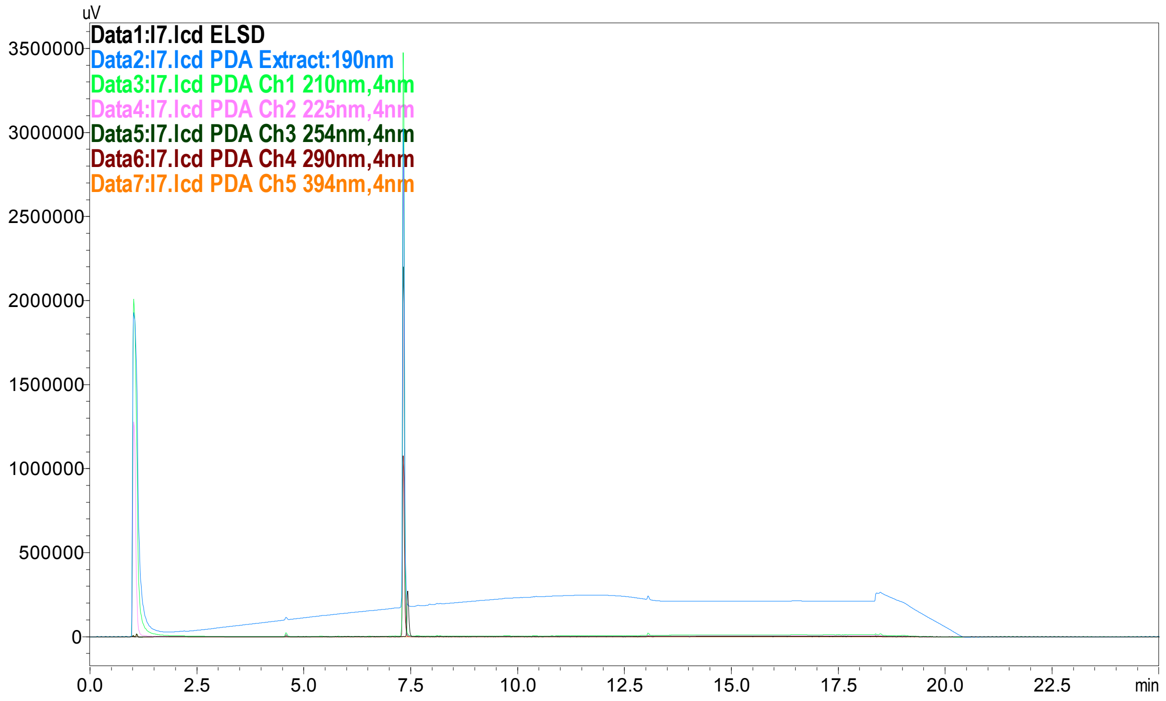

Fig S64. ^{1}H NMR of compound **28** (300 MHz, DMSO-d6)

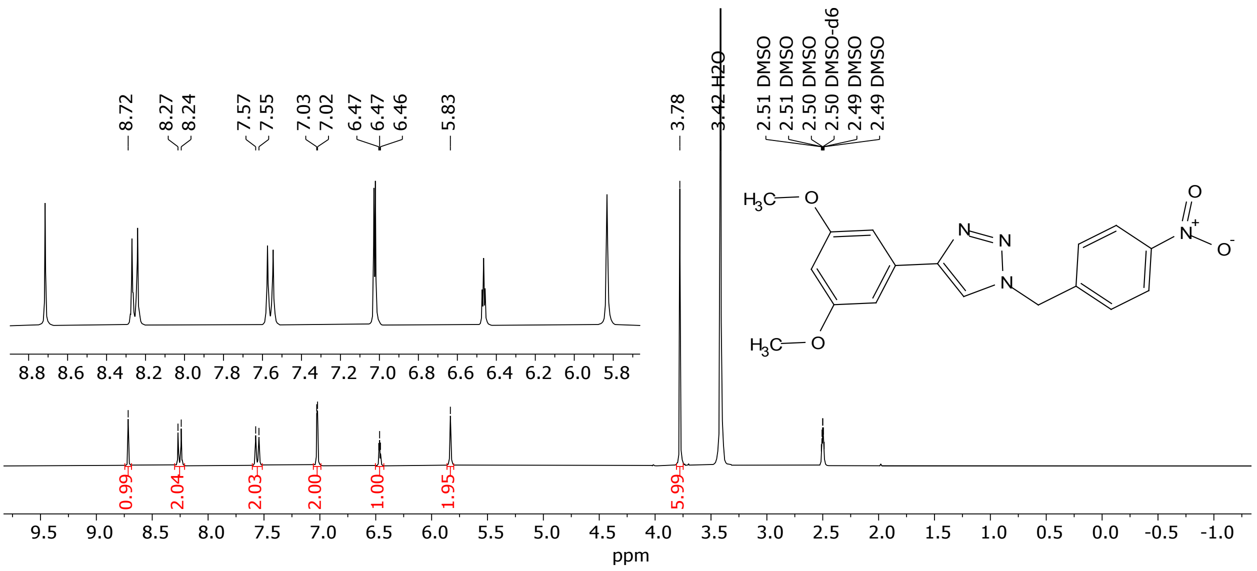

Fig S65. ^{13}C NMR of compound **28** (75 MHz, DMSO)

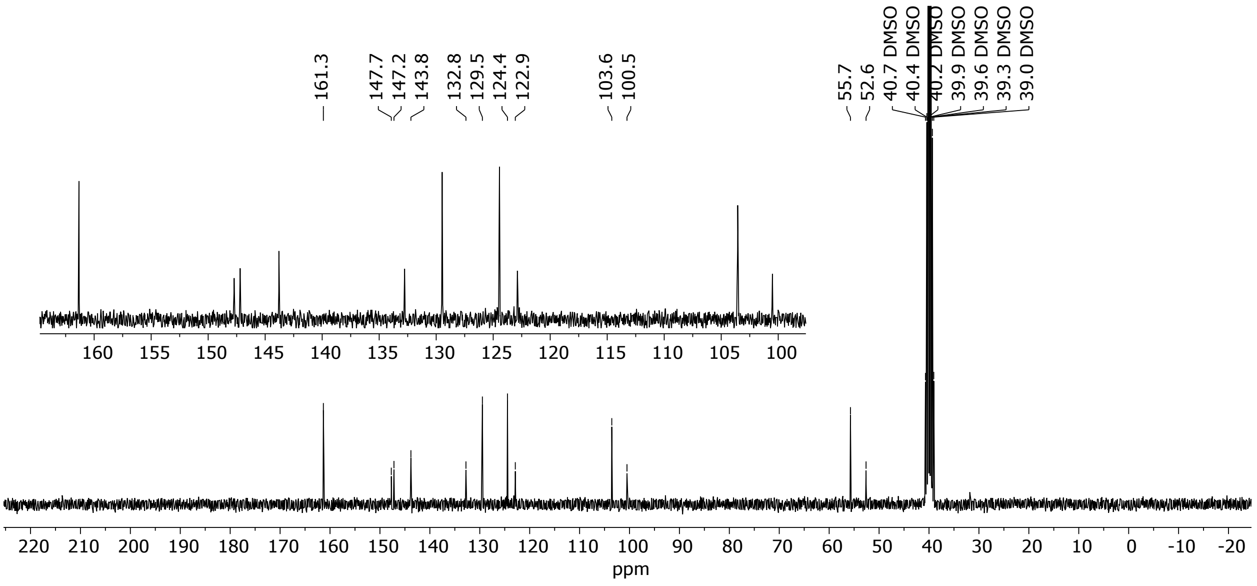

Fig S66. HRMS of compound **28**

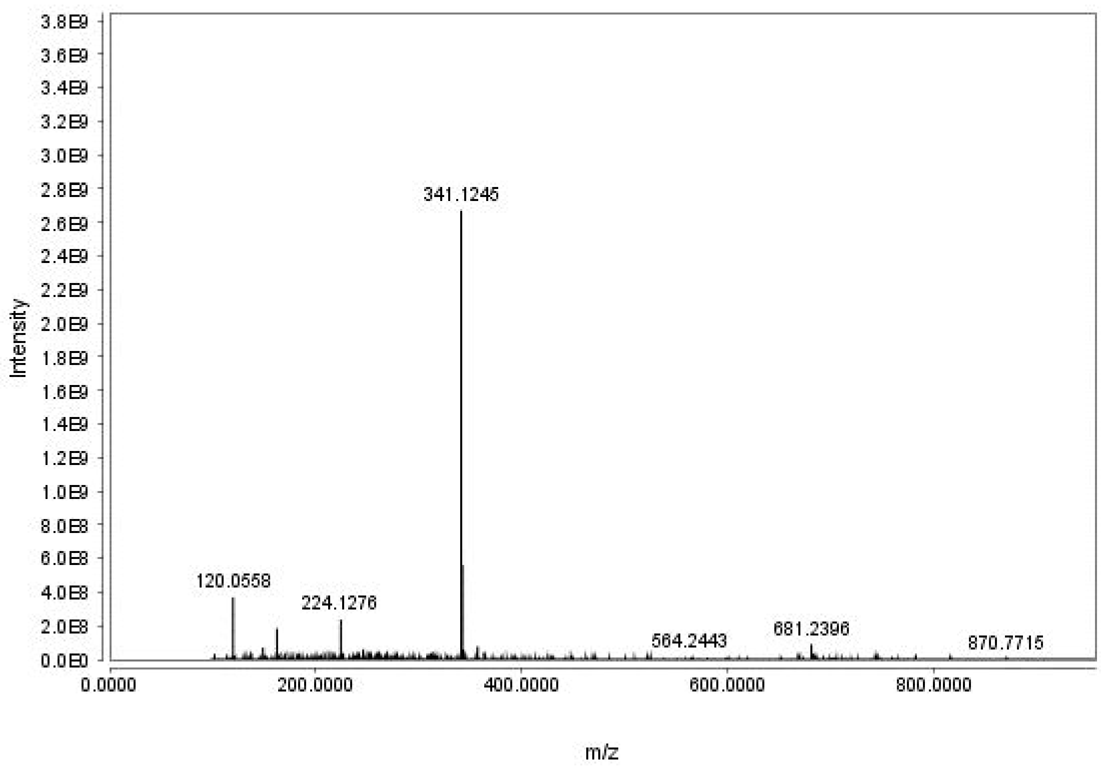

Fig S67. Chromatographic purity analysis of compound **28**

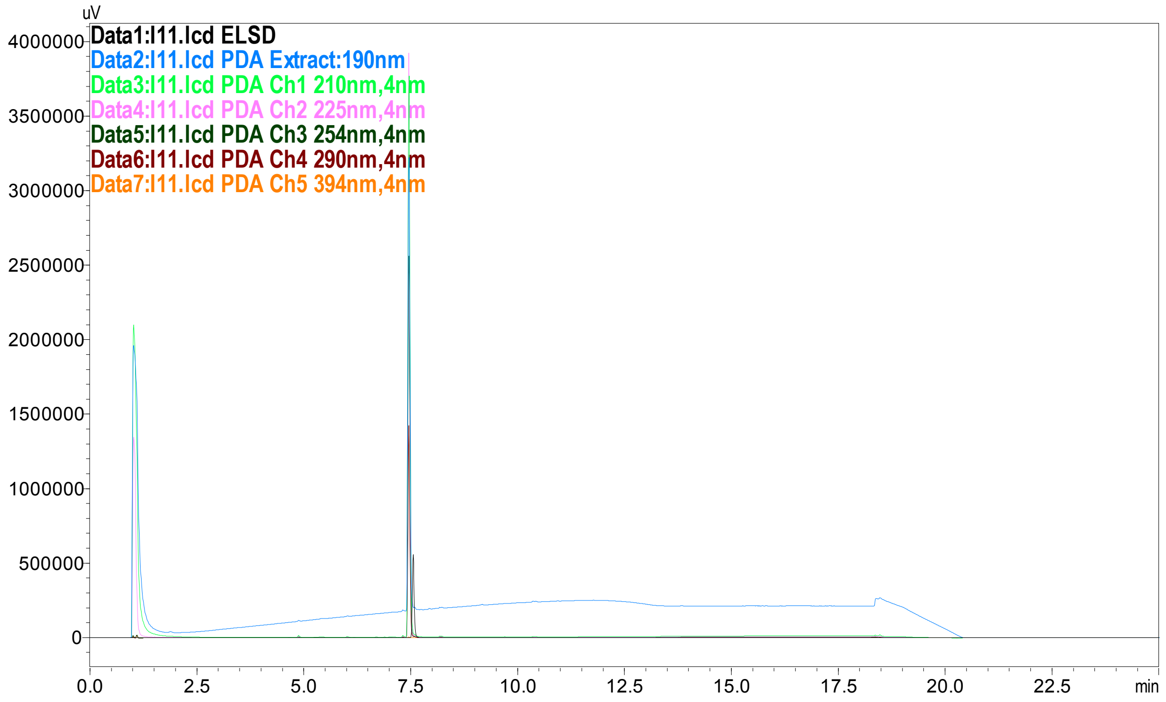

Fig S68. ^{1}H NMR of compound **24** (300 MHz, DMSO-d6)

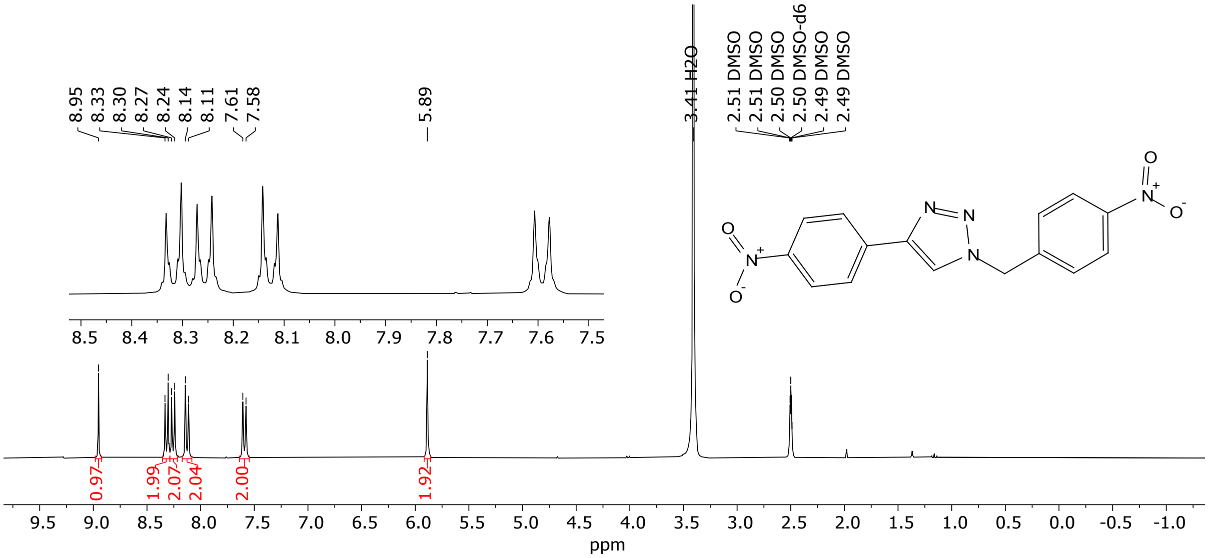

Fig S69. Chromatographic purity analysis of compound **24**

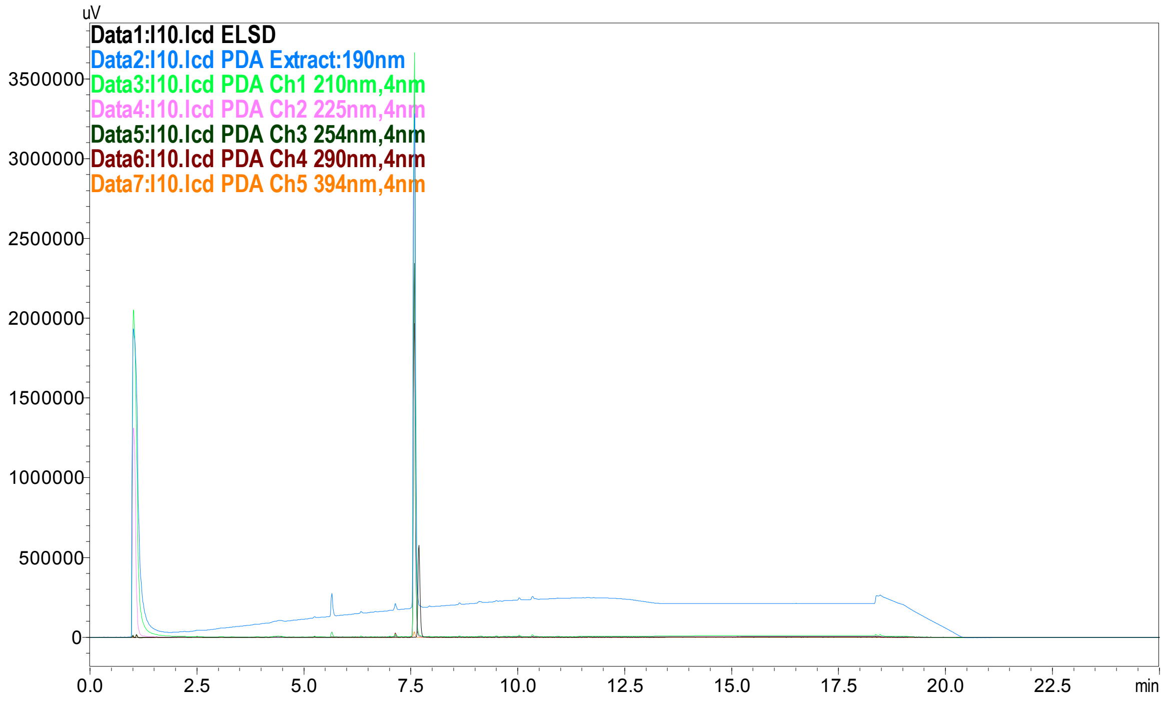

Fig S70. ^{1}H NMR of compound **23** (300 MHz, DMSO-d6)

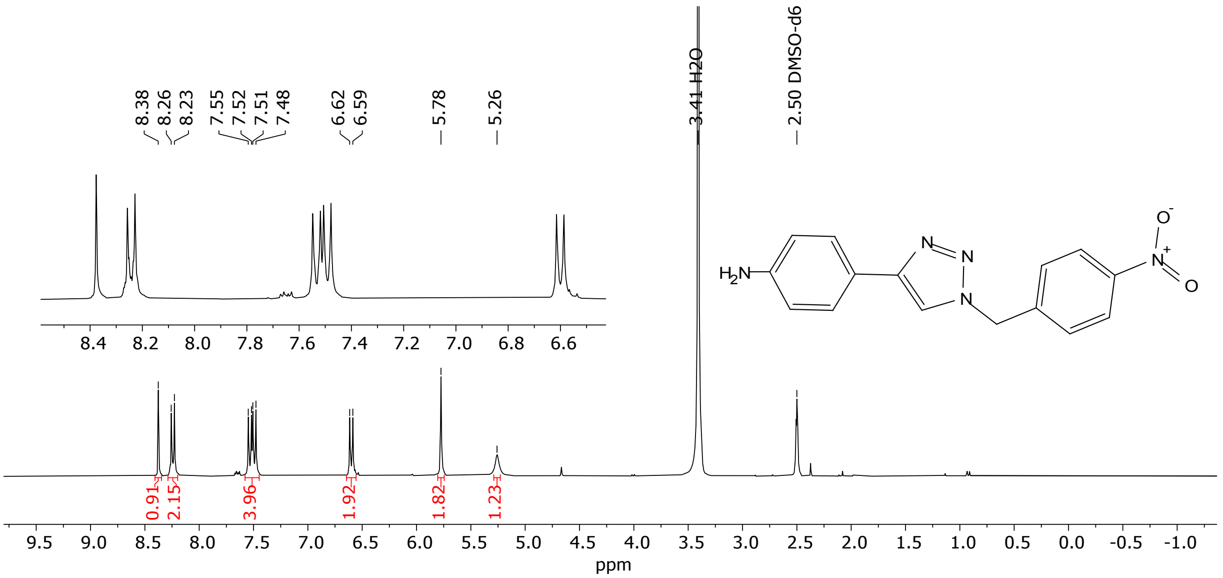

Fig S71. HRMS of compound **23**

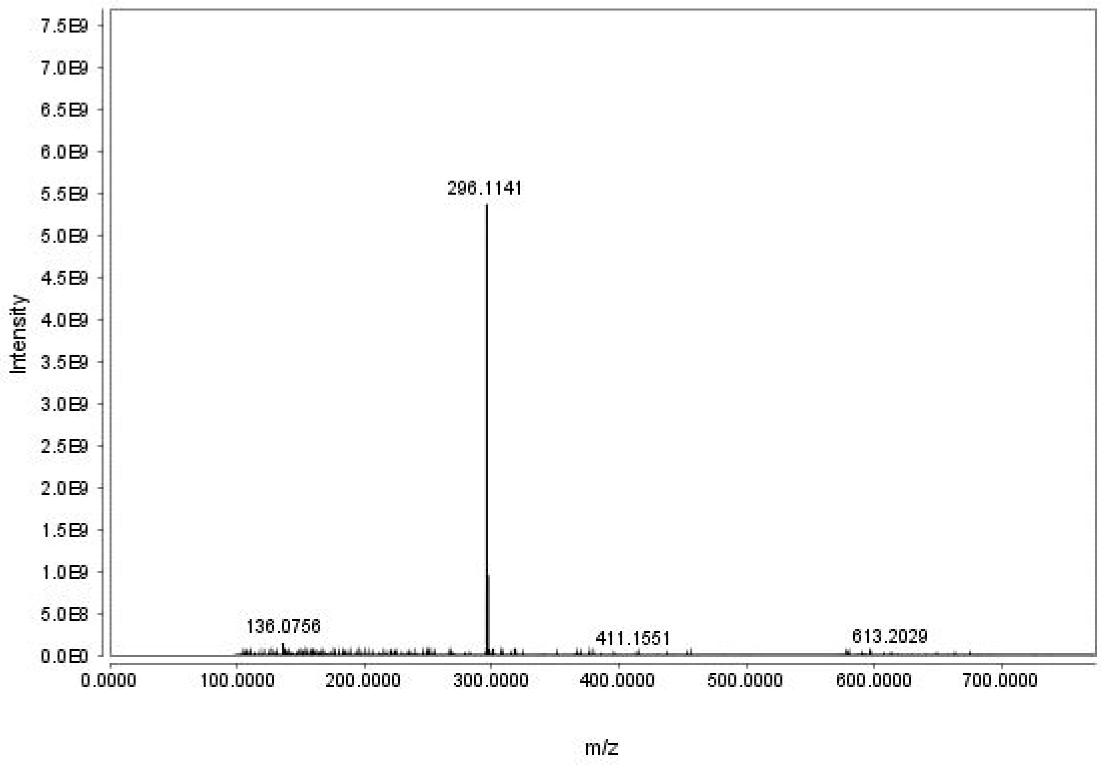

Fig S72. Chromatographic purity analysis of compound **23**

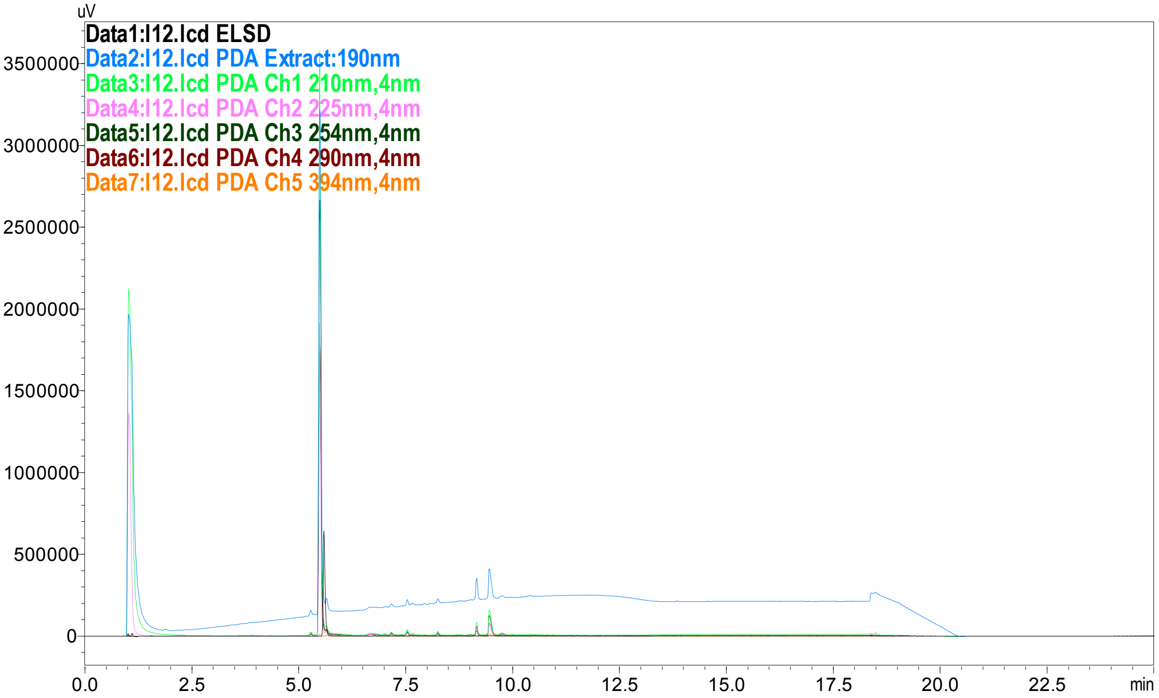

Supplement: Supplementary file 1 [file ao4c11645_si_001.pdf]
